# Assessing hypochlorite selectivity of corrosion resistance catalysts for alkaline seawater splitting

**DOI:** 10.1038/s41467-026-75110-9

**Published:** 2026-07-08

**Authors:** Jack Corbin, David Trudgeon, Cheng Lyu, Mikey Jones, Adeline Loh, Tom Mackay, Zhenyu Zhang, Xiaohong Li

**Affiliations:** 1 Department of Engineering, Renewable Energy Group, Faculty of Environment, Science and Economy, University of Exeter, Penryn Campus, Cornwall, UK; 2 Department of Earth and Environmental Science, Camborne School of Mines, Faculty of Environment, Science and Economy, University of Exeter, Penryn Campus, Cornwall, UK

**Keywords:** Hydrogen energy, Electrocatalysis, Hydrogen energy, Electrocatalysis

## Abstract

Using seawater for hydrogen production via electrolysis is increasingly attractive for cost savings. The main challenge is the competition at the anode between hydroxide oxidation and chloride oxidation, which must be minimised in both acidic and alkaline environments. Strategies such as electrostatic repulsion and ion selectivity have been used to reduce chloride oxidation in seawater, but no direct comparison has determined which is more effective. Here, we show two strategies using a rotating ring disc electrode setup to detect hypochlorite formation at the disc in an alkaline saline solution with different catalysts. We show that adding chromium to an oxygen evolution reaction catalyst reduces hypochlorite formation from 2.362 mM to 0.670 mM over 5 minutes. The electrostatic repulsion strategy (using sulphide doping) lowers the concentration to 0.966 mM under the same conditions, indicating that the ion selectivity strategy is more effective at reducing hypochlorite in alkaline seawater. However, the electrostatic repulsion strategy causes only a minor loss in electrochemical performance but improves selectivity by reducing hypochlorite formation. This research could offer insights into streamlining the design of oxygen evolution reaction catalysts and enhancing the efficiency of seawater electrolysis.

## Abstract (web summary)

Seawater electrolysis offers a cost-effective route to hydrogen production but is limited by competing chloride oxidation. Here, the authors report an in-situ method to detect chloride species and show that ion selectivity more effectively suppresses hypochlorite formation than electrostatic repulsion.

## Introduction

Hydrogen is gaining recognition for its environmental benefits and high energy density as an energy carrier and storage option. However, creating sustainable hydrogen remains expensive, as most production methods currently rely on fossil fuels, and a significant portion of the levelized cost of hydrogen (LCOH) stems from the purchase of electricity, rather than from catalyst materials and membrane electrode assemblies^1–3^. As hydrogen is set to become crucial in the transition to clean energy, the application of water electrolysis is expected to grow. Seawater electrolysis has become of particular interest in areas with high potential for renewable energy and space to build green hydrogen facilities, but these areas are also facing increasing water stress due to rising demand or changing climatic conditions. Australia, for example, has a significant renewable energy potential but is among the driest places on earth, and seasonal rainfall is expected to decrease by an additional 15% by 2030^4,5^. Currently, challenges associated with water management and the hydrogen economy are often underestimated. If these issues are not adequately managed, they could face significant opposition^4,6^. Seawater constitutes 96.5% of the global water supply, serving as a natural electrolyte for hydrogen generation and a practically endless resource^2^. Electrolysing seawater initiates an electrochemical reaction that can lead to unwanted side reactions due to complex seawater composition. The high chloride concentration (0.5 mol dm^−3^) can markedly trigger undesirable side reactions at the anode, resulting in the production of either chlorine (Cl_2_) (Eq. 1) or hypochlorite (ClO^-^) (Eq. 2), dependant on whether the environment is acidic or alkaline. These by-products can interfere with the intended anodic OER (Eq. 3) process, reducing electrolyser performance and generating hazardous and corrosive substances^7–9^. As a result, H_2_ production ceases in an electrolyser when chlorine species completely corrode the anode, resulting in electrolysis failure^10^. While alkaline seawater splitting technologies present a promising method for H_2_ production, they still need effective anodic catalysts that can operate actively and reliably in chloride-rich environments^10^.

Chlorine evolution reaction (CER)

1 $${2{Cl}}^{-}\to {{Cl}}_{2}+{2e}^{-}E_{0}=1.36{Vvs}.\,{RHE},\,{pH} < 7.5$$

Chloride oxidation reaction (ClOR)

2 $${{Cl}}^{-}+{2{OH}}^{-}\to {{ClO}}^{-}+{H}_{2}O+{2e}^{-}E_{0}=1.72{Vvs}.\,{RHE},\,{pH} > 7.5$$

Oxygen evolution reaction (OER)

3 $${4{OH}}^{-}\to {O}_{2}+{2H}_{2}O+{4e}^{-}E_{0}=1.23{Vvs}\,{RHE},\,{pH} > 7.5$$

Based on the equations above and previous literature, at pH > 7.5, the OER proceeds favourably due to a thermodynamic advantage of 480 mV; this has long been used as a strategy to reduce chloride oxidation, by developing electrocatalysts that limit the overpotential to below this value within this potential region, denoted as the alkaline design criterion^11,12^. However, this technique becomes less feasible for higher current density testing and scaling up of the technology due to greater iR drops and kinetic limitations of the OER, due to the 4-electron pathway requiring a higher overpotential and the subsequent stability of the electrocatalysts in seawater, which can jeopardise their ability to remain within the alkaline design criterion. Building on this, methods to reduce the adsorption of chloride ions onto the surface of anodes and to selectively adsorb OH^-^ ions has been implemented^13^. The more widely adopted technique, the so-called electrostatic repulsion technique (ESR), incorporates a polyanion or anionic layer as an electrostatic repulsion barrier towards Cl^-^ ions within the active material. This approach serves a dual purpose: it effectively prevents the adsorption of Cl^-^ while ensuring that the availability of active sites remains unaffected; and may enhance the catalytic activity of the electrocatalysts in saline electrolytes^7,13–16^. However, in certain circumstances, increasing electron density around the catalyst layer can also hinder OH^−^ adsorption and thus O_2_ gas evolution, meaning a high activation energy is required to overcome the thermodynamic barrier of the O-O coupling. Anionic (S^2-^ and P^3-^) and polyanionic dopants (SO_4_^2-^ and PO_4_^3-^) are the most widely used for the ESR. The idea of introducing the ion-selective method into the seawater electrolysis research area is a more recent revelation, but was first discovered in the 1960s^17^. Ion selectivity follows the theory that specific ions can be preferentially bound to the electrode surface. Following Pearson’s hard-soft acid-base principle (HSAB), where harder bases attract/bind to harder acids, the same principle is related to softer acids and bases^18,19^. Acids, characterised as electron pair acceptors, typically metal ions, whereas bases are ligands that serve as electron pair donors. Typically, metal ions exhibiting large positive charges and small ionic radii are classified as hard acids^20^.

In prior studies, the selectivity between ClOR and OER was assessed through ex-situ product analysis such as iodometric titration or colourimetry with N,N-dimethyl-p-phenylenediamine to detect chlorine and gas chromatography (GC) to measure gaseous products (O_2_)^21^. However, these ex-situ methods cannot provide multi-point data on selectivity in a single experiment, thus requiring large numbers of experiments and subjecting the data to inaccuracies.

An RRDE is proposed to be a feasible method to identify the selectivity of electrocatalysts during electrochemical testing, providing in situ selectivity data. Vos et al. developed a precise procedure in which an RRDE with IrOx at the disc/ring could detect currents associated with competing OER and CER in acidic media, but the study was limited to Cl^-^ ion concentrations lower than 100 mM, which is not representative of natural seawater (0.5 M)^22^. Later, Fujita et al. described for the first time how the RRDE can reveal beneficial selectivity information in neutral to alkaline conditions (pH 7–12), using IrO_2_ and MnO_2_, which illuminated the selectivity towards the OER in Cl^-^ containing electrolytes of 0.5 M NaCl^21^. Therefore, the RRDE provides insights into catalyst interaction and the evolution of specific species, an aspect that ex-situ methods struggle to determine due to the instability of hypochlorite.

In this study, we employed the RRDE technique to establish an accessible protocol for assessing the selectivity of various catalysts at a highly alkaline pH, commonly used in the literature on seawater electrolysis and utilising a solution containing 1 M KOH and 0.5 M NaCl to simulate seawater, without the need for costly IrO_2_ deposits on the ring. Using this method, we have compared two of the most widely reported corrosion-resistant techniques, namely the electrostatic repulsion and ion-selective strategies, and probed which technique is more effective in mitigating ClO^-^ formation and, in turn, promoting O_2_ during the electrolysis of seawater at high current densities, which is not clear in the literature.

## Results and discussion

### Corrosion-resistant techniques for OER electrocatalysts in seawater

It is well established in seawater electrolysis literature that OER electrocatalysts are required to be highly catalytically active, durable and selective^13^. As a result, research efforts have been directed toward mitigating the corrosive nature of certain anions in seawater during electrolysis, specifically the oxidation of Cl^-^ ions to ClO^-^ (Eq. 2). Here, we investigate the impact of the most established corrosion-resistant techniques derived from an established OER electrocatalyst^23^. This approach directly compares the techniques to uncover the effect of the theory and determine which technique suppresses oxidation of Cl^-^ ions more effectively, since the precursor solution and synthesis are nearly identical.

**Fig. 1:**
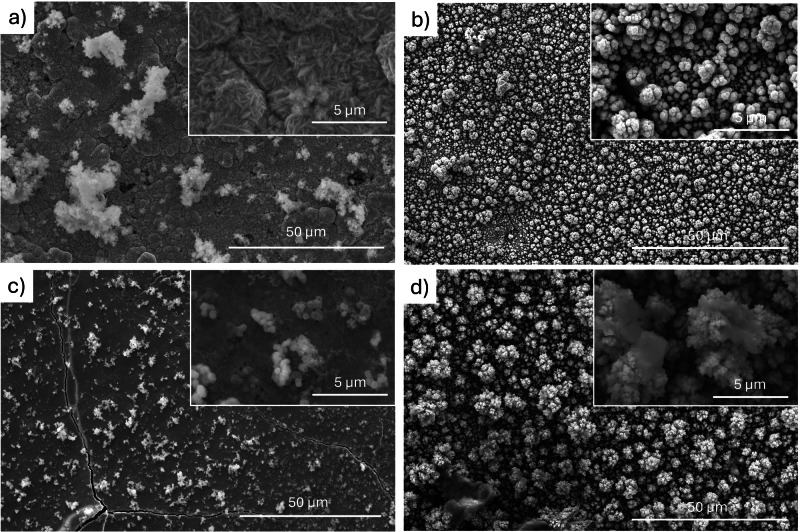
Variations in surface morphology of catalyst materials. SEM pictures of the catalysts coated carbon plate, over the same surface area as the RRDE tip (0.2376 cm^2^) **a** NiFe(OH)_2_ **b** NiFe-S **c** NiFe-Cr **d** Cr-NiFe.

This study involves applying an anionic modification, specifically a sulphide dopant, to a NiFe electrocatalyst, which in this study is denoted as NiFe-S. The presence of the negatively charged sulphide ion (S^2-^) in its structure prevents the adsorption of Cl^-^ ions to the positively charged anode surface^24^. We incorporate the sulphide dopant (using thiourea as a sulphur source) directly into the deposition solution. The SEM as revealed in Fig. 1, shows that the modifications to the precursor solution have varied the morphology of the different catalysts. The NiFe(OH)_2_ (Fig. 1a), shows a relatively dense surface with scattered clusters. The inset reveals platelet-like or needle-shaped nanosheets, with a relatively compact surface and some porosity due to mild hydrogen evolution. The lower current density produces OH^-^ slowly, resulting in moderate supersaturation and controlled nucleation^25^. The morphology of the NiFe-S (Fig. 1b) is broadly consistent with a homogeneous distribution but differs with densely packed spherical nanoparticles; the inset shows well-defined nanosphere clusters, indicating high nucleation density. Thiourea in the precursor solution functions as a complexing agent and a sulphur source. Thiourea influences electrodeposition in several ways, mainly by modifying nucleation behaviour and complexing with metal ions during growth^26^. The complex formation between thiourea and Ni²⁺ is well documented and may lead to NiFe-Sₓ-type phases or S-doped hydroxides, which usually grow as fine-grained, globular clusters due to rapid nucleation and limited crystalline order^26,27^. EDS analysis of NiFe-S (Fig. S1 and S4) revealed a uniform distribution of Ni, Fe and S across the sample. Point analysis revealed an average contribution of 77.62% Ni, 9.19% Fe and 13.18% sulphur across an inset of the sample.

To further understand the ISS, we were concerned with the effect of Cr doping on the evolution of ClO^-^. We compared modification strategies by investigating the impact of incorporating Cr within a single-step deposition during the simultaneous deposition of Ni and Fe and by exclusively depositing Cr to the ready-formed catalyst in a successive deposition step, culminating in a two-step deposition process. SEM analysis (Fig. 1c, d) reveals an interesting effect on the morphology between the addition of the Cr in a second deposition step, Cr-NiFe (Fig. 1d) and in a single deposition, NiFe-Cr (Fig. 1c). The surface of NiFe-Cr appears rougher and more cracked, with small agglomerates visible throughout, and the inset shows small spherical clusters rather than platelets. The greater HER influence compared to NiFe(OH)_2_, resulting from the higher current density, produces microcracks and microporosity due to bubble detachment^28^. The addition of Cr to the precursor solution may also compete for incorporation into the catalyst layers, disrupting the lattice. The introduction of Cr into NiFe through electrodeposition has been shown to disturb the nanosheet morphology and produce ‘holey’ structures, consistent with our observation of cracked or disrupted films in NiFe-Cr^29^.

In a two-step deposition (Fig. 1d), the morphology varies with a far rougher topography stemming from more heterogeneous clusters. The inset shows agglomerated stacked structures, with some smoother particles and secondary overgrowth. The initial NiFe layer provides a porous, conductive scaffold for the second Cr-only deposition, which leads to localised Cr(OH)_3_ precipitation on top of existing NiFe features rather than a homogenous distribution (Fig. S2). This is because Cr^3+^ has a strong tendency to hydrolyse; as a result, its deposition mainly occurs on pre-existing hydroxide nucleation sites, leading to particle aggregation^30^.

Additionally, repeated HER can further roughen and texture the surface. XPS analysis confirms the presence of Cr in both NiFe-Cr and Cr-NiFe (Fig. S7), the spectrum is split into 2 prominent peaks, centred at 586.2 eV and 576.8 eV and are assigned to Cr 2p_1/2_ and 2p_3/2_, respectively. The 2p_3/2_ spectrum is fitted with two main peaks, attributed to Cr oxide and hydroxides, centred at 576.5 and 577.5 eV, respectively^2,31–34^. This confirms that Cr is in the +3 oxidation state for both catalyst materials. Cr was chosen at the hard Lewis acid layer because Cr3+ is the hardest Lewis acid among transition metals, with a very low pKa value (2.05)^2^. Following the HSAB, the incorporation of Cr^3+^ into the catalyst will preferentially adsorb OH^-^ ions (a hard Lewis base) over Cl^-^ ions (an intermediate base). Based on this, some of those OH^-^ will participate in the OER, while the surplus will theoretically form an electrical double layer (EDL) within the Helmholtz layer and reduce the amount of Cl^-^ attracted to the electrode surface (Fig. S8)^2,45^.

EDS analysis further confirms the contribution of Cr into the NiFe. Figure S1 confirms a uniform distribution of Ni, Fe and Cr for both NiFe-Cr and Cr-NiFe. Figure S2 reveals insight into the precipitation of Cr over the top of NiFe sites, which is confirmed by EDS mapping. Point analysis showed a lower overall Cr content in the NiFe-Cr (Fig. S5), averaging 81.22% Ni, 11.86% Fe and 6.91% Cr over the sample. In comparison, Cr-NiFe (Fig. S6) averaged 74.58% Ni, 5.27 % Fe and 20.14% Cr, as a result of the areas of densely coated Cr.

### Determining RRDE collection efficiency

Before conducting RRDE experiments using catalysts, it is standard practice to estimate the collection efficiency of the ring electrode to determine the proportion of product produced at the disc that is reduced or oxidised at the ring. The collection efficiency can be determined theoretically and/or experimentally. To ensure the accuracy of the experimental setup for the tests, it is recommended to determine this parameter experimentally using a well-established reversible single-electron redox couple, such as ferricyanide-ferrocyanide (Fe(CN)_6_^3-^/Fe(CN)_6_^4-^)^21,35–37^. This redox couple operates quickly, reversibly, and is diffusion-controlled, emphasising the importance of the RRDE’s geometry and hydrodynamics over its chemistry. Subsequently, the reversibility of the redox couple means the disc and ring currents directly correlate with the fluxes of species, enabling comparison between the measured collection efficiency and the theoretical value dictated solely by electrode design. Sweeping the voltage at the disc negatively, the starting species ferricyanide is reduced to ferrocyanide, which then reaches the ring (the ring potential is held constant) and is oxidised back to ferricyanide, as shown by Eq. 4 and Eq. 5^45^.

Reduction reaction

4 $${Fe}({CN})_{6}^{3-}+{e}^{-}\to {Fe}({CN})_{6}^{4-}$$

Oxidation reaction

5 $${Fe}({CN})_{6}^{4-}\to {Fe}({CN})_{6}^{3-}+{e}^{-}$$

Quantitively, the ratio between the ring and disc diffusion-limited currents determines the collection efficiency^45^.

Collection efficiency

6 $$N=\frac{{Ring}\,{limiting}\,{current}}{{Disk}\,{limiting}\,{current}}\times 100$$

**Fig. 2:**
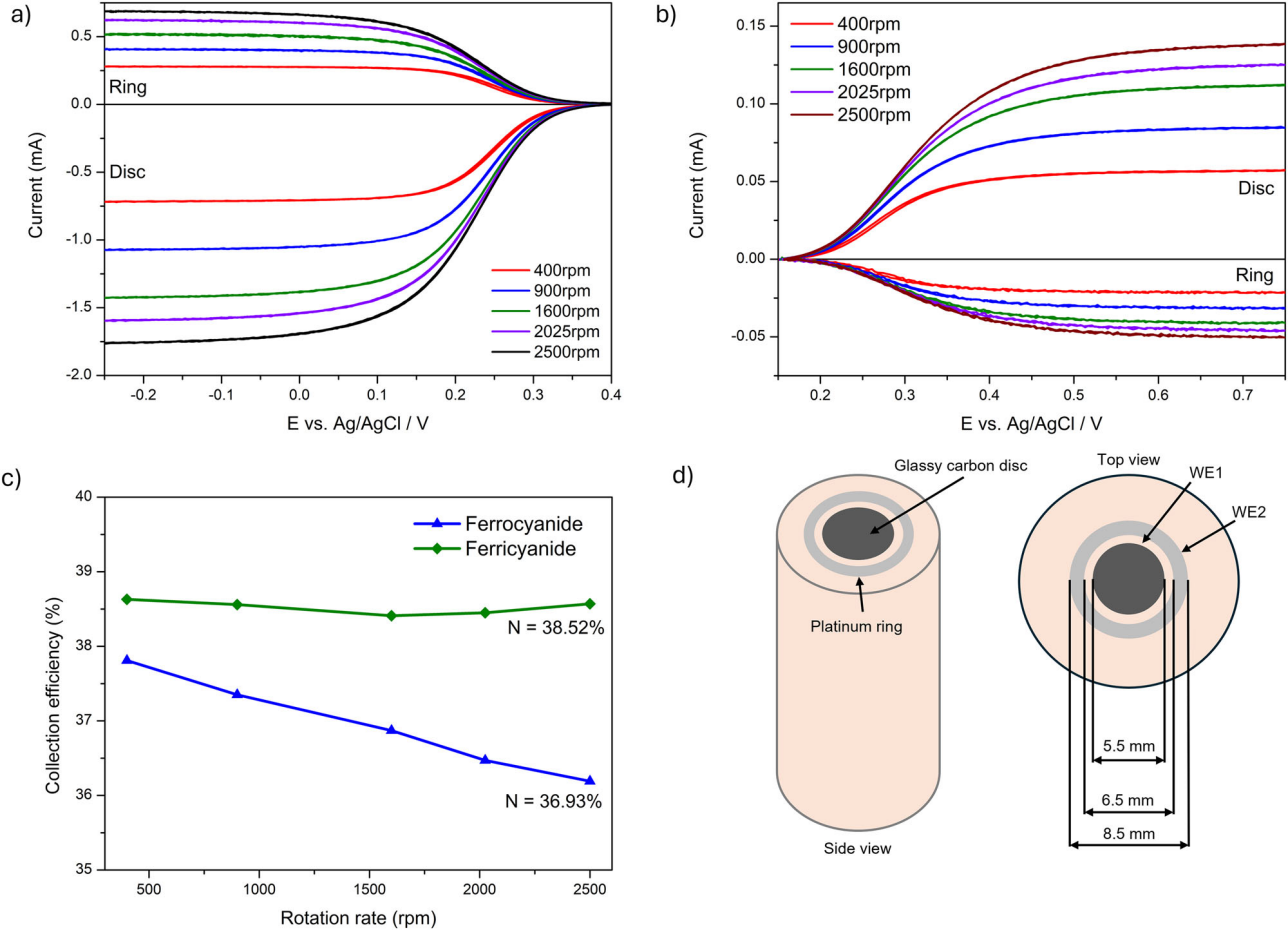
Determination of the collection efficiency for the RRDE setup. Voltammograms of RRDE with glassy carbon disc and Pt ring using a scan rate of 10 mV s^−1^ with rotation rates of 400, 900, 1600, 2025 and 2500 rpm; **a** ferri/ferrocyanide in 0.5 M KCl with 10 mM Fe(CN)_6_^3-^/ **b** ferro/ferricyanide in 0.5 M KCl and 10 mM Fe(CN)_6_^4-^. **c** Resulting collection efficiencies from the experimental RRDE solutions in both test solutions. **d** RRDE working electrode geometry; adapted from ref. ^55^ Note: E vs Ag/AgCl refers to potentials versus silver/silver chloride reference electrode, and WE1 and WE2 refer to working electrode 1 and 2. Potential obtained within these figures is left uncompensated. Disc surface area is 0.2376 cm^2^ and ring surface area is 0.2356 cm^2^. Source data are provided as a Source Data file.

While this measurement is typically achieved by starting with ferricyanide, the reverse reaction is also applicable. Therefore, we have applied both methods to ensure accuracy and reproducibility (Fig. 2a, b). According to the instrument manufacturer, a theoretical efficiency of 38.3% is stated, which is based solely on the geometry of the RRDE. From our theoretical calculations (Eqs. S1–S8), we determine the theoretical collection efficiency to be 38.2%, with the slight discrepancy related to rounding during the calculation. Experimentally (Fig. 2c) the collection efficiency ranged from 36.93% in ferrocyanide to 38.52% in ferricyanide, further confirming close agreement and reinforcing the accuracy and reliability of the electrochemical setup. Based on the setup, we have used 38.52% as the collection efficiency going forward.

### Determining the hypochlorite sensitive region using RRDE voltammetry

Here, by applying a fixed reducing potential at the ring, known to induce hypochlorite reduction, we use a RRDE to quantify the formation of ClO^-^ during seawater electrolysis in alkaline solution.

7 $${{ClO}}^{-}+{H}_{2}O\,+\,{2e}^{-}\,\leftrightarrow \,{{Cl}}^{-}+2{{OH}}^{-}$$

**Fig. 3:**
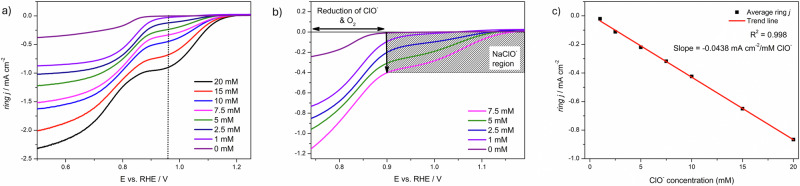
Identification of isolated hypochlorite reduction region in order to establish a constant ring potential for alkaline simulated seawater electrolytes. RRDE in 1 M KOH, pH 14 and varying concentrations of sodium hypochlorite to determine a region where hypochlorite can be detected exclusively without contribution from ORR at a rotation rate of 900 rpm **a** Concentrations of ClO^-^ from 0 mM to 20 mM **b** Concentrations of ClO^-^ from 0 mM to 7.5 mM. **c** Calibration curve using the ring current densities associated with the known concentrations of ClO^- ^as observed in graphs a and b. Note: ring j denotes the ring current density, and where NaClO^-^/ClO^-^ indicates sodium hypochlorite and hypochlorite ion. Pearson’s correlation coefficient (R^2^). mM corresponds to millimolar. Source data are provided as a Source Data file.

Figure 3 shows the RRDE tests conducted to identify a potential region where ClO^-^ can be solely detected independently from oxygen reduction reaction (ORR) influence. The RRDE was rotated at 900 rpm, whilst the rotation rate that achieved the highest collection efficiency (500 rpm) is ideal; the rotation rate must be sufficient to ensure bubble disturbance when used in the OER (Fig. 2c). The ring potential was swept cathodically between 1.3 and 0.5 V vs. RHE at a scan rate of 10 mV s^−1^ in varying known concentrations of NaClO^-^ added to 1 M KOH solutions, simulating ClO^-^ formation in a seawater electrolyte. In solutions without ClO^-^, no ring current was detected between 1.3 and 0.9 V vs RHE, as seen in Fig. 3a, b. However, with the addition of known ClO^-^ concentrations, a distinguishable ring current attributed to the reduction of ClO^-^ (Eq. 7) is observed at potentials more negative than 1.15 V vs. RHE until approximately 0.9 V vs. RHE, where the current plateaus due to diffusion limitations of ClO^-^ ions to the ring surface^21^. This provides a 0.250 V potential window, where ClO^-^ can be detected exclusively by the Pt ring.

Sweeping the potential more negatively (<0.9 V vs RHE) introduces ring currents associated with the reduction of oxygen (ORR). Therefore, a constant ring potential of 0.974 V vs RHE was selected for the detection of ClO^-^ in further experiments (dotted line Fig. 3a), as any current associated with the ring at this potential is exclusively related to the reduction of ClO^-^ to Cl^-^ based on experimental findings in Fig. 3a, b (Eq. 7). Remarkably, ring currents can detect ClO^-^ in the solution down to concentrations as little as 1 mM, highlighting the sensitivity of the setup.

Based on Fig. 3, it is possible to analyse the quantitative relationship between ClO^-^ concentration and the RRDE ring current density, of which the magnitude increases linearly with ClO^-^ concentration. A calibration curve was constructed by plotting the RRDE ring current density where the current reached diffusion limitations (0.974 V vs RHE) against known concentrations of ClO^-^. Linear regression was subsequently applied to this relationship to identify a Pearson’s correlation coefficient R^2^ value of 0.998 (Fig. 3c), indicating a strong linear relationship between ClO^-^ concentration and ring current response. This strong correlation illustrates the reliability of the RRDE technique for the quantitative detection of ClO^-^. The gradient of the line relates how much the RRDE ring current density changes per unit increase in ClO^-^ concentration. Specifically, for every 1 unit increase in ClO^-^ concentration (in this context mM), the average ring current density increases by 43.8 µA cm^−2^; ultimately, this quantifies the sensitivity of the RRDE ring current to ClO^-^.

One caveat to discuss regarding the calibration curve is that the known concentration of ClO^-^ is homogeneous throughout the solution and is assumed also to be the initial local concentrations at the RRDE, between the disc and ring. Whereas the ClO^-^ formed in situ at the disc is not homogeneously distributed in the solution, it is a localised time-dependent concentration at the ring based on the specific conditions at the disc surface, as the initial bulk concentration of ClO^-^ at the start of the test is equal to zero. Therefore, using the curve to estimate unknown ClO^-^ concentrations in subsequent electrochemical measurements should be done so cautiously, with this in mind. However, since the comparison between the ring currents of the catalysts is kept consistent with the identical starting electrolyte and parameters applied throughout, it is not affected by this and serves as a useful insight.

### Experiment methodology

**Fig. 4:**
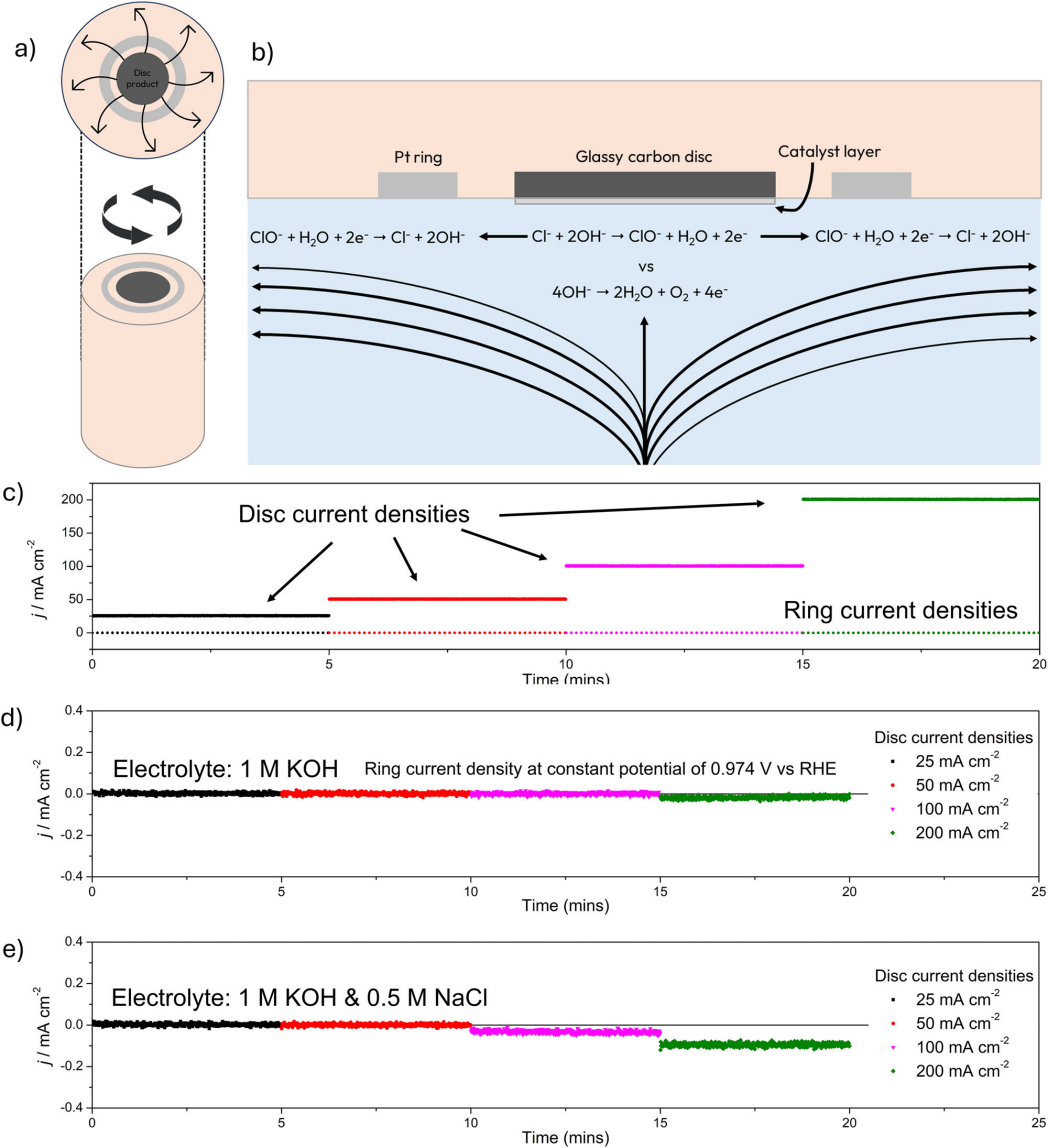
Testing of NiFe(OH)_2_ using isolated hypochlorite reduction region in alkaline and alkaline saline solutions. **a** RRDE schematic of how the disc product is transported to the ring; adapted from ref. ^55^. **b** RRDE schematic of the competing reactions occurring at the disc and the reduction of the hypochlorite at the ring **c** NiFe(OH)_2_ coated disc on RRDE in 1 M KOH with the ring potential held at 0.974 V vs RHE (0.05 V vs Hg/HgO) at a rotation rate of 900 rpm **d** ring response from increasing disc current densities in 1 M KOH **e** ring response from increasing disc current densities in 1 M KOH & 0.5 M NaCl, pH 14. Note: ring j denotes the ring current density. Source data are provided as a Source Data file.

Based on the findings above (Fig. 4), we apply the methodology to the OER experiments in alkaline and alkaline saline electrolytes (Fig. 4a, b). To evaluate its selectivity in saline electrolytes, we deposited NiFe(OH)_2_ on the disc^24^. We investigated the responses in initially 1 M KOH (Fig. 4c, d), followed by a simulated seawater electrolyte (1 M KOH and 0.5 M NaCl) (Fig. 4e), which is an established electrolyte commonly used in alkaline seawater electrolysis literature, considering that seawater is primarily water with approximately 3.5% salts by weight^13,38^. While other ions found in seawater can interfere with reactions at either electrode, the effect of Cl^-^, which is the primary focus here due to its high concentration, causes competing reactions to the desired OER^13^. The method uses CP at the disc, applying increasing current densities from 25 to 200 mA cm^−2^, while CA is applied at the ring, with the potential held at 0.974 V vs RHE. As a result, any ring current response is solely attributable to the reduction of ClO^-^ formed at the disc electrode, allowing in-situ detection of ClO^-^. Based on this, the observed ring current can be used to determine the occurrence of ClOR and resulting ClO^-^ concentration in the solution.

Figure 4 shows the implementation of the methodology on an established catalyst, NiFe(OH)_2_^23^. Fig. 4d confirms that no significant ring current response is observed while increasing the disc current density anodically in 1 M KOH, confirming that the deposited catalyst has no effect on the ring current during the experiment. However, in a saline electrolyte of 1 M KOH & 0.5 M NaCl Fig. 4e show that as disc current density increases, particularly at higher OER current densities (>100 mA cm^−2^), a noticeable ring current is observed, indicating the formation of ClO^-^ from the oxidation of Cl^-^ ions at the disc and the subsequent partial reduction of ClO^-^ at the ring (Eq. 7).

An average ring current density is taken across the 5-minute period of each disc current density. At 100 and 200 mA cm^−2^, ring current densities of −32.5 and −94.8 µA cm^−2^ are observed, respectively, with NiFe(OH)_2_ applied to the disc.

Our approach to determining the formation of ClO^-^ differs from typical methods of identifying disc products at the ring, as we utilise the respective CP and CA rather than sweeping the potential at the disc using cyclic voltammetry^21,22^. Our reasoning is that sweeping the potential at scan rates of 1 to 10 mV s^−1^ does not provide sufficient time for evolved ClO^-^ to reach a concentration that is detectable. This method, with constant current/potential, further aligns with which a catalyst would be applicable under full electrolyser cell conditions.

### Determining the OER selectivity of corrosion-resistant OER electrocatalysts

**Fig. 5:**
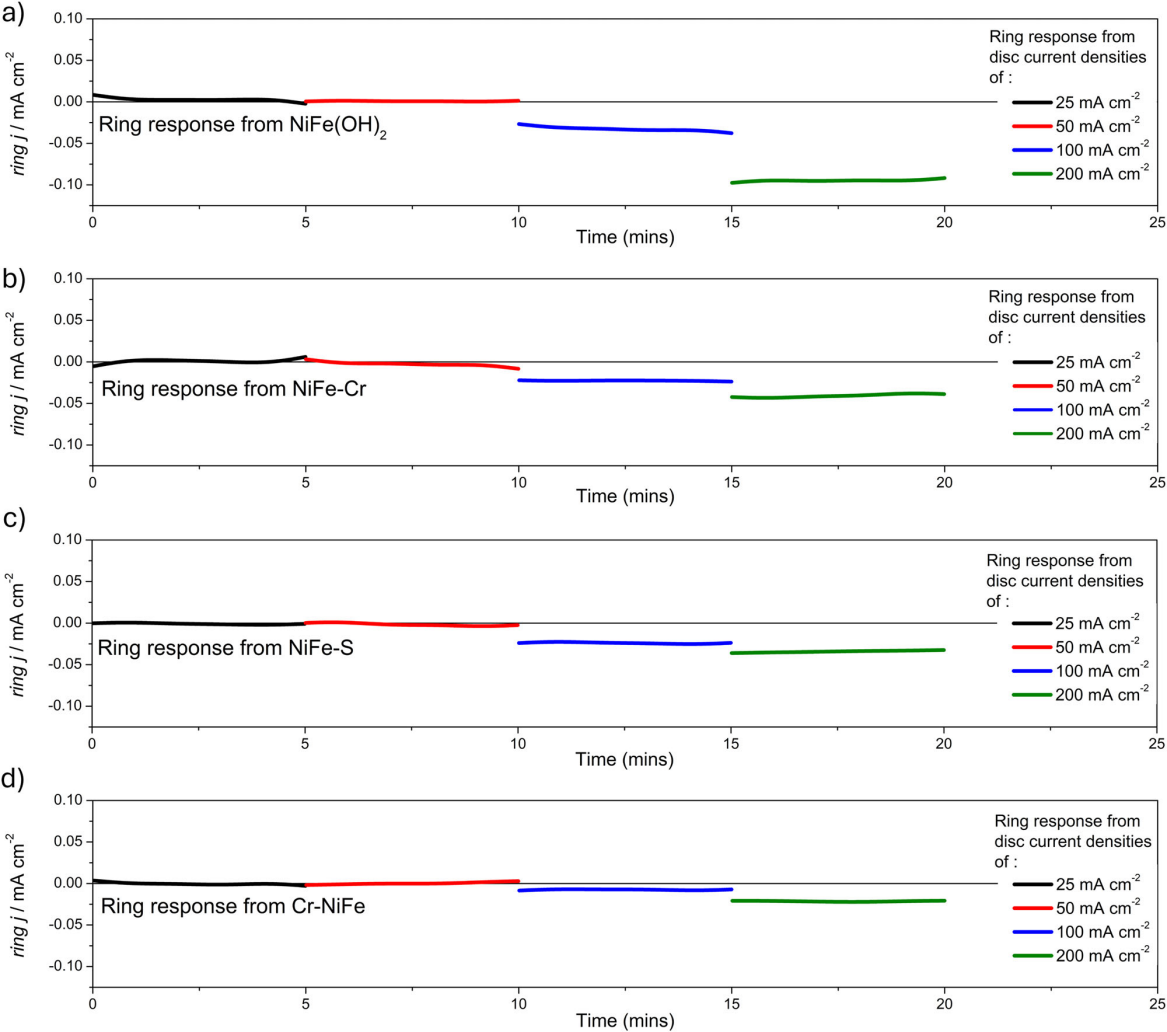
Identification of ring current density variations from catalyst materials in alkaline saline solution. RRDE in 1 M KOH & 0.5 M NaCl, pH 14, with the ring potential held at 0.974 V vs RHE (0.05 V vs Hg/HgO) at a rotation rate of 900 rpm at 298 K, with catalyst coated on the disc **a** NiFe(OH)_2_ **b** NiFe-Cr **c** NiFeS **d** Cr-NiFe. Note: ring j denotes the ring current density. Source data are provided as a Source Data file.

To thoroughly assess the selectivity of the catalysts, we applied the same methodology as above to each catalyst individually in 1 M KOH & 0.5 M NaCl. Figure 5 reveals a consistent trend that shows applying a steadily increasing disc current density results in a noticeable ring current density at disc current densities of 100 mA cm^−2^ or higher in simulated seawater for all catalysts. However, a noticeable difference is observed between the catalysts of corrosion-resistant techniques. Figure 5 shows that the unmodified NiFe(OH)_2_, produces the largest ring current densities of −32.5 and −94.8 µA cm^−2^ at 100 and 200 mA cm^−2^ disc current densities, respectively. Linking it to the evolution of the largest amount of ClO^-^ generation based on the above theory. Interestingly, the addition of Cr in a single deposition reduces the ring current by 31.07% at 100 mA cm^−2^ to −22.4 µA cm^−2^ and by 57.79% at 200 mA cm^−2^ to −40.01 µA cm^−2^ relative to the unmodified NiFe(OH)_2_ (Fig. 5b). Remarkably, applying the Cr layer in a secondary deposition step, to overlay the Cr layer on the surface of the NiFe(OH)_2_ demonstrates an interesting effect (Fig. 5d), ring current densities of -6.9 µA cm^−2^ and −20.7 µA cm^−2^ are observed at 100 and 200 mA cm^−2^, highlighting a 69.64% and 48.26% reduction compared to NiFe-Cr, respectively, and a 78.76% and 78.16% reduction compared to NiFe(OH)_2_. It is clear that applying Cr to the surface in a second electrodeposition step has a direct effect on the amount of ClO^-^ evolved at the disc, which supports the theory that specific ions (i.e. OH^-^) can preferentially attract to the electrode surface (Fig. S8).

Furthermore, the ESR strategy (Fig. 5c), also demonstrates an effective reduction in ring current at high disc current densities, with a 26.76% reduction in ring current at 100 mA cm^−2^ and a 64.45% reduction in ring current at 200 mA cm^−2^, compared to NiFe(OH)_2_. Interestingly, comparing the ESR to the ISS, it appears that the ISS (applying a hard Lewis acid into the catalyst and more specifically to the surface) is a more effective method within these materials to reduce the formation of ClO^-^ in simulated seawater. At 100 mA cm^−2^, the secondary deposition modification to the ISS produces 71% less ring current (which is attributable to ClO^-^ reduction at the ring), than the ESR and at 200 mA cm^−2^, it produces 38.57% less than the ESR.

Further to the focus on the ring current, the observation of disc potential at the set current densities using chronopotentiometry is also an important metric, providing additional information about the selectivity of each catalyst. Since the formation of ClO^-^ is mainly driven by the disc potential, it is essential to correlate the disc potential to the ring currents, to ensure that it is not the activity of the catalysts that is influencing the ring current densities, but rather the modification techniques (ISS or ESR)^39^. The Pourbaix diagram has largely dictated the literature on the 480 mV overpotential gap between the OER and COR, where if electrocatalytic oxidation can be conducted at potentials below 1.72 V vs. RHE for complete seawater electrolysis and with an overpotential for the oxygen evolution reaction OER of less than 480 mV in alkaline electrolytes, then the formation of hypochlorite is theoretically prevented, as it is thermodynamically suppressed within this potential range, thereby resulting in almost 100% selectivity for OER^40,41^.

**Fig. 6:**
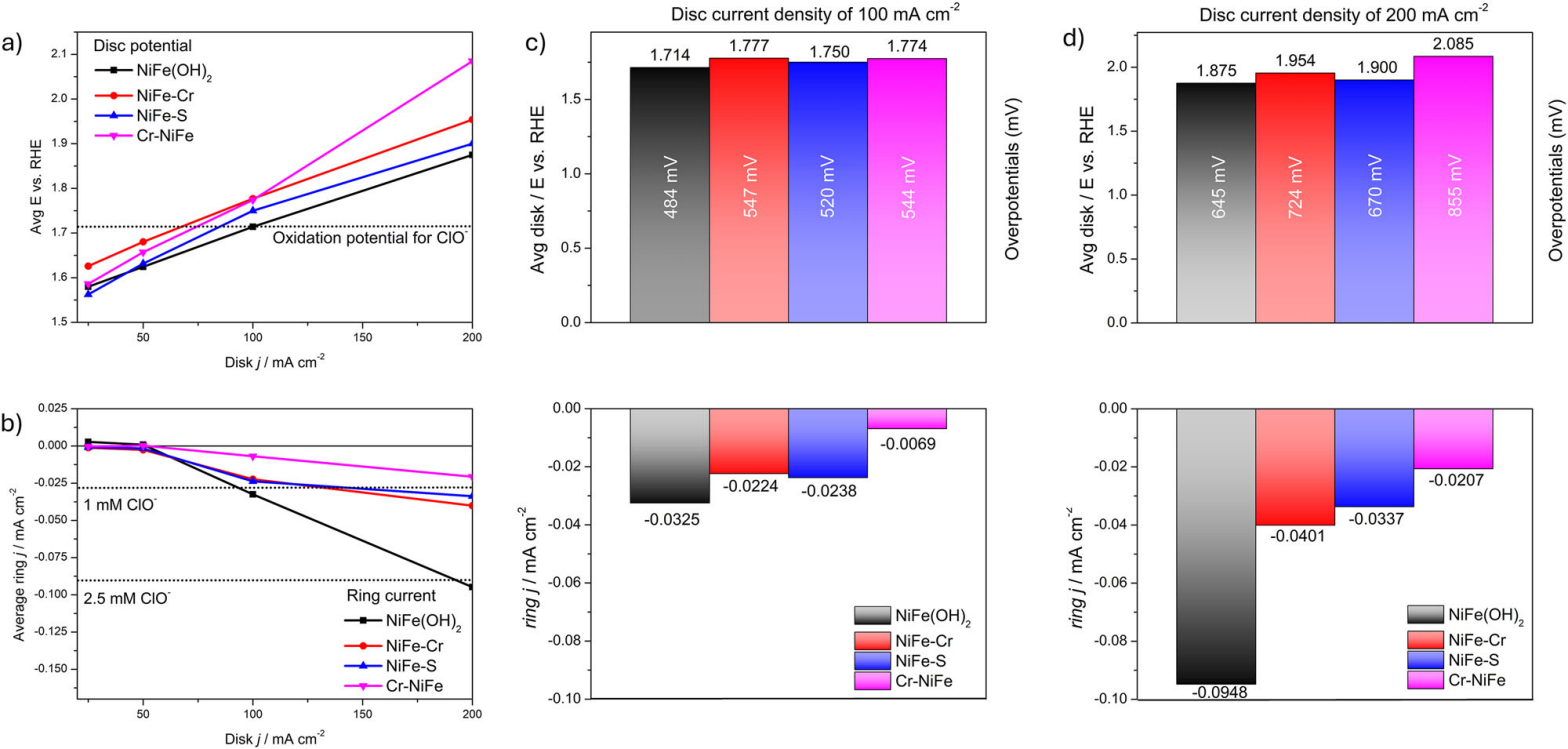
Variations in ring current density and disc overpotential of catalyst materials in simulated seawater. RRDE in 1 M KOH & 0.5 M NaCl, pH 14 at a rotation rate of 900 rpm at 298 K, with different catalyst coated on the disc **a** average disc potential taken over the 5 min period at different anodic disc current densities disc. **b** Ring responses from the corresponding disc current densities as set out in graph a, relating the quantity of ClO^-^ formed during the reaction. **c** Average disc potentials recorded with catalyst coated GC disc at 100 mA cm^−2^ with corresponding ring current densities. **d** Average disc potentials recorded with catalyst coated GC disc at 200 mA cm^−2^ with corresponding ring current densities. Note: ring j denotes the ring current density, and E vs RHE refers to potentials versus reversible hydrogen electrode. The potential values within this figure are not iR compensated. Resistance values (ohms): NiFe(OH)_2_ 8.357, NiFe-Cr 9.699, NiFe-S 7.655 and Cr-NiFe 7.676. Source data are provided as a Source Data file.

Theoretically, the potential at the disc drives the formation of ClO^-^ on the surface of the catalysts, thus the catalyst with the largest overpotential at the disc would be the catalyst that thermodynamically would produce the greatest amount of ClO^-^. However, the results in Fig. 6 reveal a different effect, Cr-NiFe has the largest disc voltage 2.085 V (largest overpotential 855 mV), at a disc current density of 200 mA cm^−2^ but induces the lowest ring current of −20.7 µA cm^−2^, compared to that of NiFe(OH)_2_, which poses the lowest overpotential between the materials of 645 mV at a disc current density of 200 mA cm^-2^ but with the greatest ring current density of -94.8 µA cm^−2^. Moreover, Cr-NiFe produces 78.16% less ClO^-^ than NiFe(OH)_2_ (Fig. 6d), which demonstrates that the formation of ClO^-^ is not solely driven by the thermodynamic potential but can also be influenced by modifying the catalyst and interfacial chemistry within the electrical double layer. The addition of the ISS to the Cr-NiFe reduced the formation of ClO^-^, emphasising the ClO^-^ formation suppressing effect of the corrosion-resistant techniques implemented into the catalyst (Fig. 6d). A similar trend can be observed between NiFe(OH)_2_ and NiFe-S at a disc current density of 200 mA cm^−2^ (Fig. 6d), both illustrate disc overpotentials of 645 and 670 mV, respectively. Yet, the ring current density for NiFe(OH)_2_ is 64.45 % higher than that of NiFe-S, illustrating the benefit of the ESR theory over the thermodynamic preferability and further supporting the hypothesis that the nature of the catalyst modification strategy has a considerable effect on ClO^-^ formation/suppression. It is worth noting that due to the high disc current density applied, some noise is observed (Fig. S11a, b) across the ring and disc data from the significant gas evolution. The noise is attributed to bubbles formed at the disc that disperse radially. Bubble transport is observed not to perturb the ring current since a steady state current is achieved (Fig. S11b); a more fractured and highly variable ring current would be observed across the 5-min period otherwise, since the bubbles would block ClO^-^ detection at the ring either completely or sporadically.

From Fig. 6 it is evident that applying a Cr containing species to the catalyst material results in a higher overpotential with respect to NiFe-S and NiFe(OH)_2_ as observed in Fig. 6a, since some of the highly active catalyst sites have been covered/replaced with a less conductive coating of Cr(OH)_3_ and Cr_2_O_3_ (Fig. S2)^42^.

Overall, the protocol suggests that it is not exclusively the catalyst’s activity (seen through the thermodynamic overpotential) driving the formation of ClO^-^, as catalysts that require a larger overpotential to achieve a fixed current density would produce the greatest amount of ClO^-^; the protocol suggests that it is rather the specific corrosion-resistant technique applied (Fig. S8).

### Quantifying the selectivity of corrosion-resistant OER electrocatalysts

**Fig. 7:**
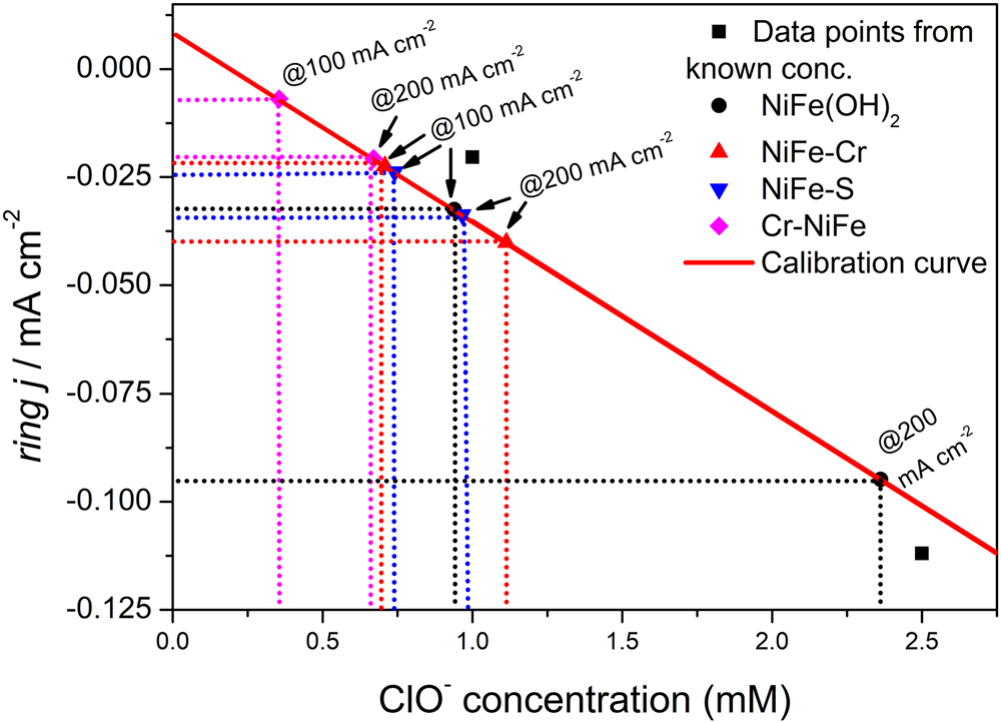
Quantification of ring current densities to known hypochlorite concentrations. RRDE in 1 M KOH and different concentrations of sodium hypochlorite with the ring potential held at 0.974 V vs RHE (0.05 V vs Hg/HgO) at a rotation rate of 900 rpm. Note: ring j denotes the ring current density. Source data are provided as a Source Data file.

To gain further insight into the selectivity of the corrosion-resistant catalysts, it is possible to utilise the associated ring currents to make further estimations based on the catalyst materials used in this study. Based on the calibration curve determined in Fig. 7, showing a high linearity (R^2^ = 0.998), it demonstrates an clear correlation between ClO^-^ concentration and ring current density and is suitable for estimating quantitative analysis. Measured ring current densities from catalysts tested were substituted into the calibration equation to determine the corresponding ClO^-^ concentrations at the ring. Tables 1 and 2 reveal insights into the estimated ClO^-^ concentrations for each catalyst in the localised region between the disc and ring that are specific to this particular RRDE setup. More precisely, at zero displacement from the ring, the defined concentration refers to a very small volume very close to the ring. It is interesting to estimate localised concentrations of ClO^-^ because of the corrosive effects on the electrode surface. Based on the ring currents observed in Fig. 5 and 6, Table 1 was used to determine the rate of ClO^-^ production on the catalyst, as calculated using Figs. 5 and 6. The rate of ClO^-^ production is more directly related to typical localised corrosion and is independent of different electrolyte volumes and various cell hardware. Since the ClOR rates are based on the ring currents observed in the experiments, the trend observed in Fig. 5 and 6 is maintained throughout Table 1. To provide perspective based on these values, since the scale is difficult to quantify, we estimate that the highest ClOR rate is observed with NiFe(OH)_2_. To generate 1 mM of ClO^-^ in 100 ml of electrolyte at 200 mA cm^−2^, it would take approximately 3.85 days, assuming no ClO^-^ decay, no significant catalyst decay and the suitability of the collection efficiency in modelling the transport of hypochlorite from the disc to the ring using Eqn S12 to Eqn S15^43^. Cr-NiFe, in comparison, with the lowest rate of ClOR observed, under the same conditions, would take approximately 17.64 days (Table 1).

**Table 1:** Estimated localised production of hypochlorite from the ring current densities

Catalyst	Rate of ClOR (mmol cm^−2^ s^−1^)	Localised ClO^-^ concentration to reach 1 mM dm^−3^ in bulk solution (days)		
	At 100 mA cm^−2^	At 200 mA cm^-2^	At 100 mA cm^−2^	At 200 mA cm^−2^
NiFe(OH)_2_	4.336 × 10^−6^	1.265 × 10^−5^	11.23	3.85
NiFe-Cr	2.988 × 10^−6^	5.348 × 10^−6^	16.30	9.11
NiFe-S	3.175 × 10^−6^	4.503 × 10^−6^	15.34	10.82
Cr-NiFe	9.205 × 10^−7^	2.762 × 10^−6^	52.92	17.64

Rates of chloride oxidation reaction (ClOR) between disc and ring during electrochemical testing in 1 M KOH & 0.5 M NaCl based on ring current densities and estimated calculations for localised ClO^-^ production rates to reach 1 mM bulk electrolyte concentration.

**Table 2:** Estimated Faradaic efficiency and hypochlorite production from known concentrations

Catalyst	ClOR Faradaic efficiency (%)	Estimated concentration of ClO^-^ produced derived from calibration curve		
	At 100 mA cm^−2^	At 200 mA cm^−2^	At 100 mA cm^−2^	At 200 mA cm^−2^
NiFe(OH)_2_	0.084	0.122	0.939 mM	2.362 mM
NiFe-Cr	0.058	0.052	0.708 mM	1.113 mM
NiFe-S	0.061	0.043	0.740 mM	0.966 mM
Cr-NiFe	0.018	0.027	0.355 mM	0.670 mM

ClOR Faradaic efficiency and estimated localised concentration of ClO^-^ between disc and ring during electrochemical testing in 1 M KOH & 0.5 M NaCl. Estimated concentrations are based on average ring currents after 5 min of electrolysis. Note: ClOR Faradaic efficiency was derived from the ring currents observed in Fig. 6. Thus, an element of deviation will be observed from these values as ring currents deviate.

To provide a more general estimation and link the ring currents to the calibration curve (Fig. 3c). Table 2 shows that the calculated concentrations based on extrapolation from Fig. 4c ranging from ~0.355 to ~2.362 mM, reflecting varying oxidative strength in the samples; NiFe(OH)_2_ produces the most significant amount of ClO^-^ at 200 mA cm^−2^, corresponding to 2.362 mM, whereas Cr-NiFe under the same conditions produces only 0.670 mM. These results demonstrate that RRDE effectively detects and quantifies hypochlorite in solutions across a broad concentration range. Lower ring currents correspond to decreased ClO^-^ levels, consistent with the electrochemical behaviour of oxidising species at the ring electrode. Importantly, ring current densities significantly below the y-intercept (−8.65 µA cm⁻² (Eqn S9)) were interpreted as below the quantifiable limit of the calibration curve; only Cr-NiFe at a disc current density of 100 mA cm^-2^ produced a ring current density below that value, all other tests were within detection boundaries.

8 $${FE}{ClO}=\frac{{I}_{R}\times {n}_{D}}{{I}_{D}\times {n}_{R}\times N}$$

To gain further insight into the quantification, an RRDE can also be used to estimate FE, which indicates the proportion of total current used for the designated electrochemical process reaction. Based on this, we use the established Eq. 8 to calculate FE^43–45^. I_D_ & I_R_ stand for disc and ring current, respectively. Collection efficiency (N) - a geometric factor of the RRDE system and has been calculated previously using a ferri/ferro redox couple (38.52%) (Eqn S8), and n_D_ & n_R_ are the number of electrons transferred for each molecule of product generated at the disc & ring electrode, respectively. In an alkaline solution, hypochlorite can be electrochemically reduced at the ring (Eq. 7), a 2-electron reduction process.

The calculated FEs for the catalysts are shown in Table 2. The values range from 0.122 to 0.020 % at 200 mA cm^−2^, meaning that only a minimal amount of the current at the disc contributed to ClO^-^ formation, which is then detected at the ring. While the low FE for ClO^-^ suggests that other processes are consuming the majority of the current, including the OER as the dominant reaction, direct quantification of FE for OER is not achievable because the potential at the ring was held at a value at which exclusively ClRR occurs. The region we highlighted in Fig. 3 shows a distinguishable region for ClRR and a combined reduction region for ORR and ClO^-^ at potentials less than 0.9 V vs RHE; therefore, identifying a region for pure oxygen will have contributions from ClO^-^ reduction. A further caveat contributing to the low FEs is the nature of the reaction and the high evolution of gas bubbles, which can cause deviations in the collection efficiency compared to the previously determined value. This is because bubbles transiently block pathways and the interface between the disc and ring. Several studies highlight this issue encountered when applying inert hydrophobic catalysts to the disc^46–50^. However, it is assumed that this is not the primary issue here, since using our methodology, this issue would have prevented the detection of ClO^-^ completely, and it is estimated that the catalysts applied here are hydrophilic and demonstrate sufficient surface roughness to aid bubble detachment.

Through the use of an RRDE, we have implemented a protocol that utilises OER electrocatalysts to reveal insights into selectivity in highly alkaline simulated seawater electrolytes (pH 14 in 1 M KOH and 0.5 M NaCl), allowing catalysts with different corrosion prevention characteristics to be compared. We use a Pt ring to selectively reduce ClO^-^ formed at the disc by holding it in a region (0.974 vs. RHE) that is only sufficient to reduce ClO^-^ over the ORR at this pH range. We have compared two established corrosion-resistant techniques to identify which method is more effective in reducing the formation of ClO^-^. Using the protocol in this study, we have found that incorporating Cr into OER catalysts has a direct effect on reducing the formation of ClO^-^ based on ISS. Moreover, the application of Cr in a subsequent synthesis step (to add Cr contributions to the surface), remains the most selective for OER at 100 and 200 mA cm^−2^ with a COR_FE_ of 0.018% and 0.027%, respectively. At the same time, both corrosion reduction strategies for OER catalysts (ISS and ESR) are effective methods to reduce the formation of ClO^-^ compared to an unmodified catalyst. It is the combination of high catalytic activity (a crucial criterion for OER electrocatalysts) and selectivity of NiFe-S using the ESR that allows us to conclude this is the more effective overall strategy for these particular materials. NiFe-S produces a COR_FE_ of 0.061% and 0.043% at 100 and 200 mA cm^−2^, respectively, over a 5-min testing period. Overall, the methodology has enabled estimation of which established corrosion-resistant technique among these materials is more effective at reducing the formation of ClO^-^ during the electrolysis of simulated seawater.

## Methods

### Materials and chemicals

Materials used during synthesis and electrochemical measurements are as follows: Pine E6R2 Rotating ring disc electrode (RRDE) and MSR evo Electrode Rotator. Glassy carbon (GC) served as the working electrode (WE1) with a disc surface area of 0.2376 cm^2^ and a platinum (Pt) ring surface area (WE2) of 0.2356 cm^2^, potassium hydroxide (Sigma Aldrich, ≥90%), sodium chloride (Sigma Aldrich, ≥99%), potassium ferricyanide, Fe(CN)_6_^3-^ (Thermo scientific, ≥99%), potassium ferrocyanide (Fe(CN)_6_^4-^) (Scientific Laboratory Supplies, ≥99%), potassium chloride (KCl) (Sigma Aldrich, ≥99.5%), isopropyl alcohol, sodium hypochlorite solution, (Honeywell, 6−14% active chlorine basis), C_3_H_8_O (Sigma-Aldrich, ≥99.5%), hydrochloric acid, HCl (Sigma Aldrich, 36.5–38.0%), iron (II) sulphate heptahydrate FeSO_4_.7H_2_O (Sigma-Aldrich, ≥99%), Nickel (Ni) (II) sulphate hexahydrate, NiSO_4_.6H_2_O (Sigma-Aldrich, ≥98%), Chromium (III) sulphate hydrate Cr(SO_4_)_3_.xH_2_O (Sigma-Aldrich, ≥99%), thiourea, CS(NH_2_)_2_ (Hogg laboratory supplies, ≥99.5%), ammonium sulphate, (NH_4_)_2_SO_4_ (Sigma-Aldrich, ≥99%), reference electrode (Hg/HgO (fabricated in-house) in alkaline electrolytes) and (Ag/AgCl in acidic electrolytes), and Pt mesh counter electrode. Polished carbon plates (SGL Carbon Bipolar Plates, FR10) for SEM analysis of materials. A three-electrode electrochemical cell, 150 ml in volume, was purchased from Pine Instruments and used throughout the tests. Deionised water (DI) from an Elga Biopure 600 water processor (conductivity <30 μS cm^−1^) was used to prepare all aqueous solutions. All chemicals were used as received. Electrolytes used for electrochemical testing were prepared at the start of the dataset and kept consistent throughout the dataset. KOH was prepared in a beaker within a cold water bath to minimise the exothermic nature of the reaction and to avoid the solution boiling. Electrolytes were kept in a glass bottle and stored airtight away from direct light.

### Electrode preparation

For catalyst electrodeposition, a GC disc served as the working electrode with an active area of 0.2376 cm^2^. An Ag/AgCl reference electrode and a Pt mesh counter electrode were used in the deposition process. Before every electrodeposition process, the working electrode substrate was polished with a 0.3 and 0.05 µm alumina slurry, rinsed with IPA, and then left to dry. The Pt counter electrode was ultrasonically cleaned in 1 M HCl for 5 min, rinsed with DI H_2_O, and ultrasonically cleaned for a further 5 min.

### Synthesis of NiFe(OH)_2_

For the electrodeposition process, a three-electrode setup was used: a GC RRDE tip (ø = 5.5 mm) as the working electrode, an Ag/AgCl in 3 M NaCl electrode as the reference, and a Pt mesh as the counter electrode. The electrodes were immersed in a jacketed glass cell with about 60 mL of solution. To prepare the electrodeposition solution, an 18 mM mixed metal sulphate solution with a Ni:Fe molar ratio of 4:1 was used. The chemicals were weighed out, and a new solution was prepared for each deposition. Electrodeposition of NiFe(OH)_2_ was performed via chronopotentiometry (CP) using a Biologic Potentiostat (Biologic SP-300) and EC-lab software. The CP process applied a steady cathodic current density of −2.5 mA cm^−2^ for 5000 s at room temperature (298 K) on the RRDE tip without rotation. Following the electrodeposition, the RRDE tip was taken out of the cell and rotator, then rinsed carefully with DI water.

### Synthesis of electrostatic repulsion catalyst (ESR)

**Fig. 8:**
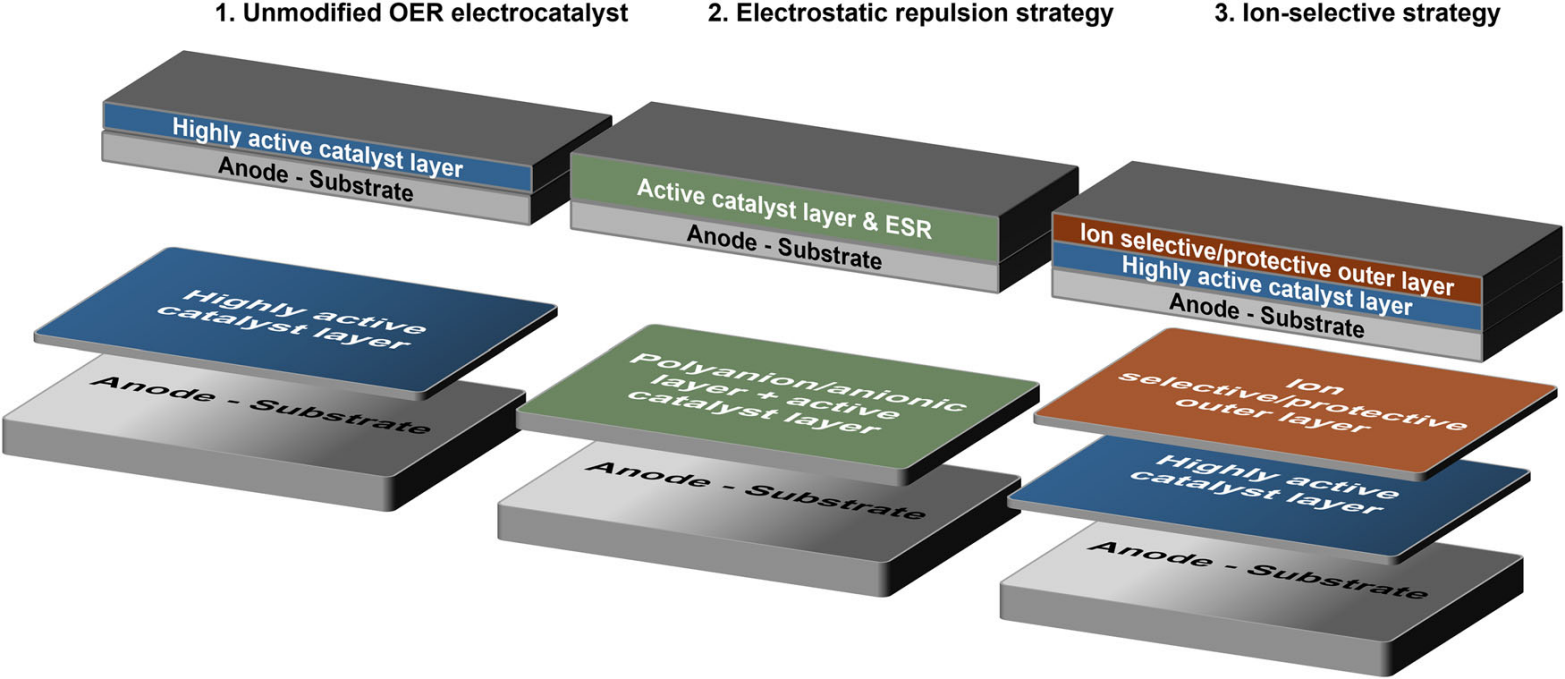
Schematic demonstrating the theory behind each catalyst coating and the layered structure. Note: ESR relates to the electrostatic repulsion strategy.

The electrodeposition conditions for the ESR catalyst were based on a modified synthesis for the NiFe(OH)_2_ catalyst, which yielded an 18 mM metal hydroxide mixture. For the ESR, thiourea (CS(NH_2_)_2_) was added to the deposition solution to serve as the sulphur source. To accommodate the dopant, the ratios were altered within the 18 mM metal sulphate mix equating to a molar ratio of 75% Ni, 12.5% Fe, and 12.5% thiourea. The one-step electrodeposition of catalysts was conducted using CP with a Biologic Potentiostat at a constant current density of −5 mA cm^−2^ and a deposition interval of 2100 seconds at 298 K, resulting in a charge passed of 2.4948 C. Similarly, after the electrodeposition, the RRDE tip was taken out of the cell and rotator, then rinsed carefully with DI water. The aim of this synthesis is for the thiourea to be incorporated into the NiFe catalyst and the sulphur residues to facilitate the OER and aid chloride repellency without impeding the active sites of the catalyst (Fig. 8).

### Synthesis of ion-selective catalyst

Synthesising the ion-selective catalyst was approached using two methods. Initially, the same deposition method as for ESR was used, with the ion-selective method incorporating chromium (Cr) into the 18 mM metal sulphate mix at a molar ratio of 75% Ni, 12.5% Fe, and 12.5% Cr. Similarly, a one-step electrodeposition was conducted using CP with a Biologic Potentiostat at a constant current density of −5 mA cm^−2^ and a deposition interval of 2100 s at 298 K. The catalyst synthesised using this approach is denoted NiFe-Cr. The aim of this synthesis was to embed Cr within the catalyst layer to identify whether a hard Lewis acid (Cr) improved the selectivity of a catalyst while being embedded into the deposit.

To probe the effect of Cr and determine whether its application order affects selectivity e.g. a surface layer, we prepared a subsequent catalyst via two-step electrodeposition. The initial deposition was conducted with a molar ratio of 75% Ni to 12.5% Fe in the precursor solution, using CP with a constant current density of −5 mA cm^−2^ at a deposition interval of 2100 s s at 298 K. The tip was removed, rinsed thoroughly, then remounted and immersed in a solution of pure Cr sulphate at the same concentration as in the single deposition (2.25 mM). The deposition parameters remained identical (−5 mA cm^−2^ for 2100 s), aiming to distribute the Cr homogenously above the NiFe deposit and maintain the same charge passed. This catalyst was denoted Cr-NiFe to illustrate Cr being on the surface of the NiFe sites.

### Electrochemical measurements

Electrochemical measurements were conducted using a Bio-Logic Potentiostat SP-300 in EC-Lab software (version 11.62.5) connected to a 4-electrode system. The setup utilised BioLogic’s bi-potentiostat configuration, which enabled the simultaneous study of two working electrode connections. The GC disc served as the first working electrode (WE1), with the Pt ring being the second (WE2). A Mercury/mercury oxide (Hg/HgO) electrode and a Graphite rod electrode served as the reference and counter electrodes, respectively.

### Voltammetry used for determining collection efficiency

Cyclic voltammetry (CV) was used to sweep the potential at the disc (WE1) and ring (WE2) simultaneously to determine the collection efficiency. Based on an established redox couple, the disc and ring currents can then be used to calculate the collection efficiency. A 150 ml glass cell was used for the electrochemical testing with 100 ml of electrolyte. The collection efficiency experiments occurred on a cleaned, uncoated GC tip and Pt ring. CVs were collected using a scan rate of 10 mV s^−1^.

### Voltammetry used for determining catalyst selectivity

Following the electrodeposition process, the WE1 was removed from the cell, and the tip was rinsed thoroughly with DI water. The RRDE tip was remounted onto the rotator and inserted into the cell setup for electrochemical testing using 1 M KOH and 1 M KOH + 0.5 M NaCl simulated seawater electrolyte. To determine the selectivity of the catalyst using the RRDE, we applied CP at the disc, while performing chronoamperometry (CA) at the ring, this allowed for a constant current density to be applied to the disc while maintaining a constant potential at the ring. CVs were collected using a potential range of 1.3–0.5 V vs. RHE with a scan rate of 10 mV s−^1^. Resistance measurements of the electrochemical setup were conducted using the current interrupt method. Precautions were taken, such as minimising cable length to reduce the detection of stray capacitances and inductances. Current interrupt measurements were employed because they enable rapid determination of uncompensated resistance under electrolysis conditions without requiring equivalent circuit fitting, and they are the most widely used method for evaluating ohmic drop and ohmic resistance in electrochemical systems^51–53^. However, as the current interrupt accounts for the complete cell resistance, there is a risk of overcompensating the potentials. Considering within this study, the magnitude of potential correction approaches the thermodynamic hypochlorite evolution window, deriving scientific conclusions based solely on the accuracy of the uncompensated resistance determination is likely to lead to inaccuracies; it is for this reason that potentials are left uncompensated. Resistance values obtained across the dataset are shown in Fig.S12 and where relevant within the figure captions.

### Physical characterisation

A scanning electron microscope (SEM), specifically the FEI Quanta FEG 650, was used to analyse the catalyst morphology on the carbon substrate^54^. The composition of the materials was analysed using Energy Dispersive X-ray Spectroscopy (EDS) with a TESCAN VEGA3 GMU SEM fitted with an Oxford Instruments X-Max 80 mm^2^ energy dispersive X-ray spectrometer^54^. The samples were placed on polished carbon plates to minimise the impact of the Ni substrate during EDS analysis. Electrodes were masked with non-conductive tape to restrict the substrate surface area to 0.2376 cm^2^, matching the surface area of the glassy carbon disc RRDE tip. Afterwards, the samples were washed with DI water and dried prior to characterisation. X-ray photoelectron spectroscopy (XPS) data was acquired at the Henry Royce Institute for Advanced Materials. XPS data was acquired using a PHI XPS Versaprobe III with a monochromatic Al Kα source (hν = 1486.6 eV, 15 kV anode voltage, 25 W beam power). Spectra have been calibrated during analysis to the carbon 1 s spectrum (adventitious carbon) set to 284.8 eV. Measurements were recorded at a base pressure (<9 × 10^−^^9 ^Torr) and at temperature of 298 K. Data was analysed using CasaXPS v2.3.19. Peak fitting used a Shirley background before component analysis.

## Supplementary information

Supplementary Information

Transparent Peer Review file

## Source data

Source Data

## Data Availability

The data supporting this article have been included in the source data files attached to this manuscript/publication. Source data are provided with this paper.
