# Supplementary material for: Assessing hypochlorite selectivity of corrosion resistance catalysts for alkaline seawater splitting
Source: Nat Commun. 2026 Jul 8;17:8089. doi: 10.1038/s41467-026-75110-9 (PMC13458344; doi:10.1038/s41467-026-75110-9)
Supplement: Supplementary file 1 [file 41467_2026_75110_MOESM1_ESM.pdf]

Supplementary information for assessing hypochlorite selectivity of corrosion resistance catalysts for alkaline seawater splitting

Jack Corbin [1], David Trudgeon [1, 2], Cheng Lyu [1], Mikey Jones [1], Adeline Loh [1], Tom Mackay [1], Zhenyu Zhang [1], Xiaohong Li [1*]

[1] Renewable Energy Group, Department of Engineering, Faculty of Environment, Science and Economy, University of Exeter, Penryn Campus, Cornwall TR10 9FE, UK

[2] Camborne School of Mines, Department of Earth and Environmental Science, Faculty of Environment, Science and Economy, University of Exeter, Penryn Campus, Cornwall TR10 9FE, UK

Email: X.Li@exeter.ac.uk *Corresponding Author

Supplementary information.

1. Theoretical collection efficiency

Equation 1: $N = 1 - F\left(\frac{\alpha}{\beta}\right) + \beta^{\frac{2}{3}}[1 - F(\alpha)] - (1 + \alpha + \beta)^{\frac{2}{3}}\left\{1 - F\left[\left(\frac{\alpha}{\beta}\right)(1 + \alpha + \beta)\right]\right\}$

Equation 2: $\alpha = \left(\frac{r_2}{r_1}\right)^3 - 1$

Equation 3: $\beta = \left(\frac{r_3}{r_1}\right)^3 - \left(\frac{r_2}{r_1}\right)^3$

Equation 4: $F(\theta) = \frac{3^{\frac{1}{2}}}{4\pi} ln\left[\frac{\left(1+\theta^{\frac{1}{3}}\right)^3}{1+\theta}\right] + \frac{3}{2\pi} tan^{-1}\left(\frac{2\theta^{\frac{1}{3}}-1}{3^{\frac{1}{2}}}\right) + \frac{1}{4}$

Equation 5: $\alpha = \left(\frac{3.25}{2.75}\right)^3 - 1 = 0.6506 = 0.651$ to (3 d.p.)

Equation 6: $\beta = \left(\frac{4.25}{2.75}\right)^3 - \left(\frac{3.25}{2.75}\right)^3 = 2.0405 = 2.041$ (3 d.p.)

Equation 7: $N = 1 - 0.527 + 2.041^{\frac{2}{3}}[1 - 0.630] - (1 + 0.651 + 2.041)^{\frac{2}{3}}\{1 - 0.713\}$

Equation 8: $N = 1 - 0.527 + 0.595 - 0.686$

N = 38.2 % collection efficiency

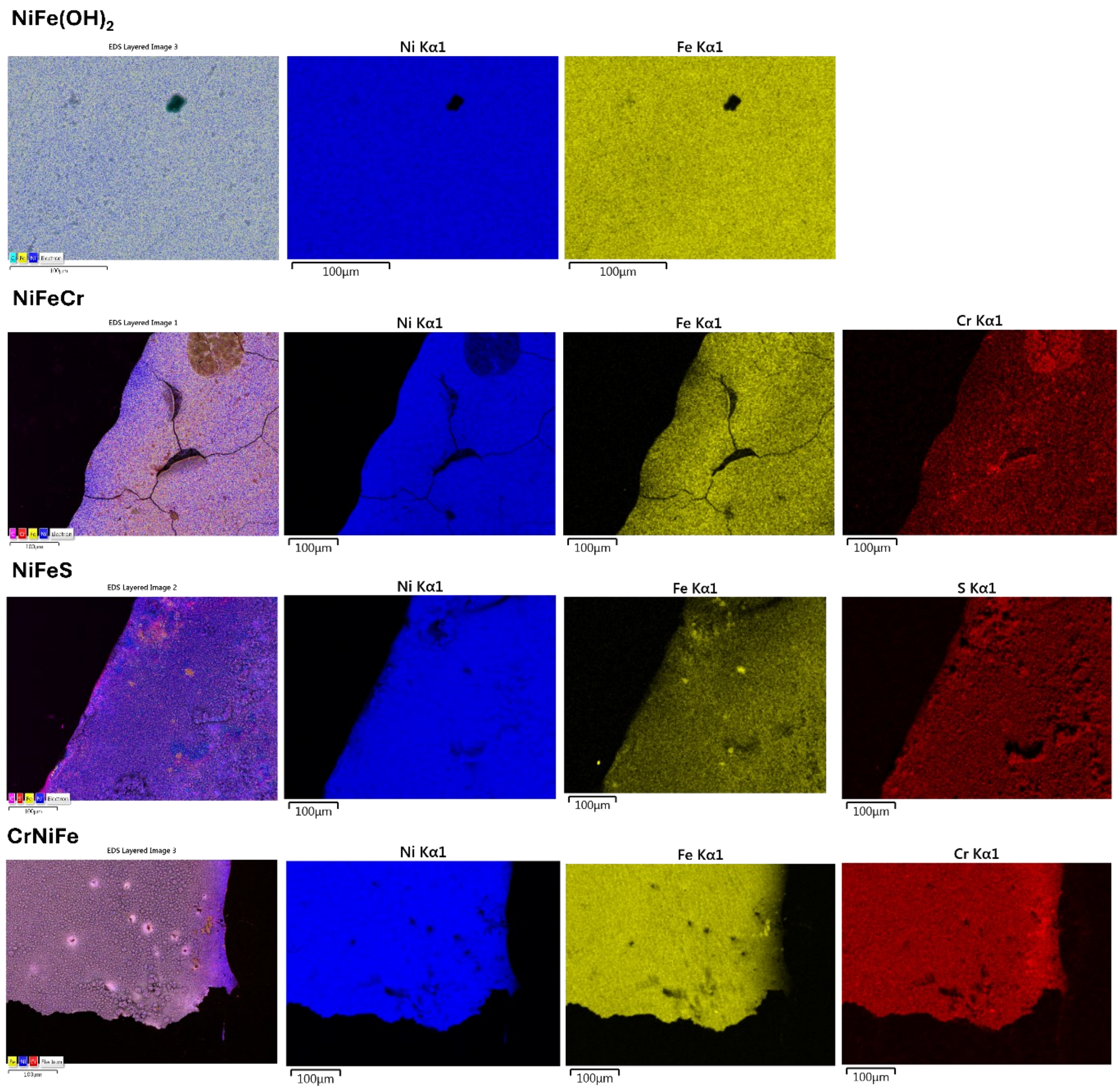

Fig. S1 Elemental distribution of the catalyst materials. EDS map data collected on tested samples prepared with different corrosion-resistant techniques.

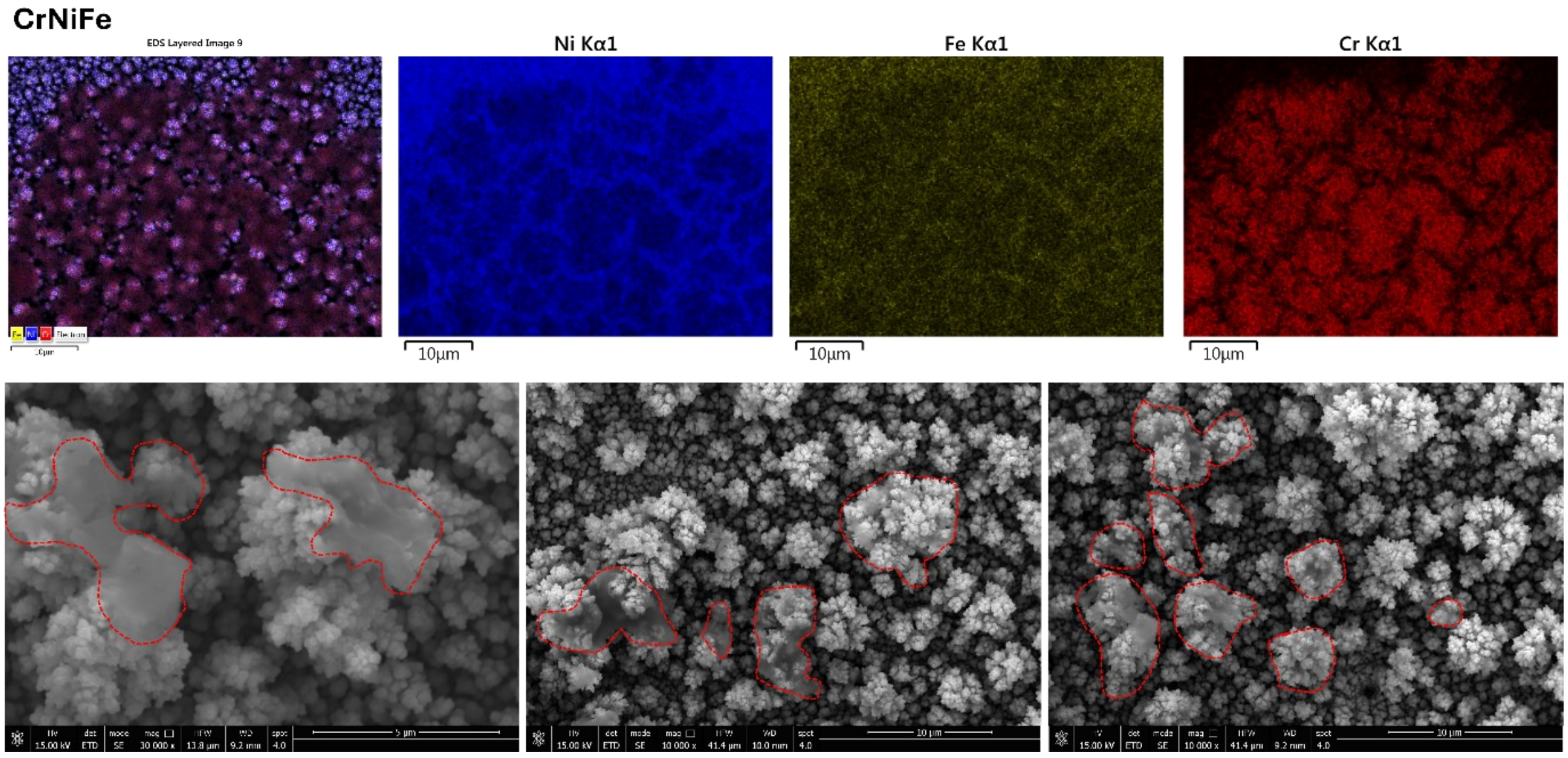

Fig. S2 Specific sites of chromium on the Cr-NiFe catalyst. EDS map data at a higher magnification for CrNiFe to illustrate the Cr coating.

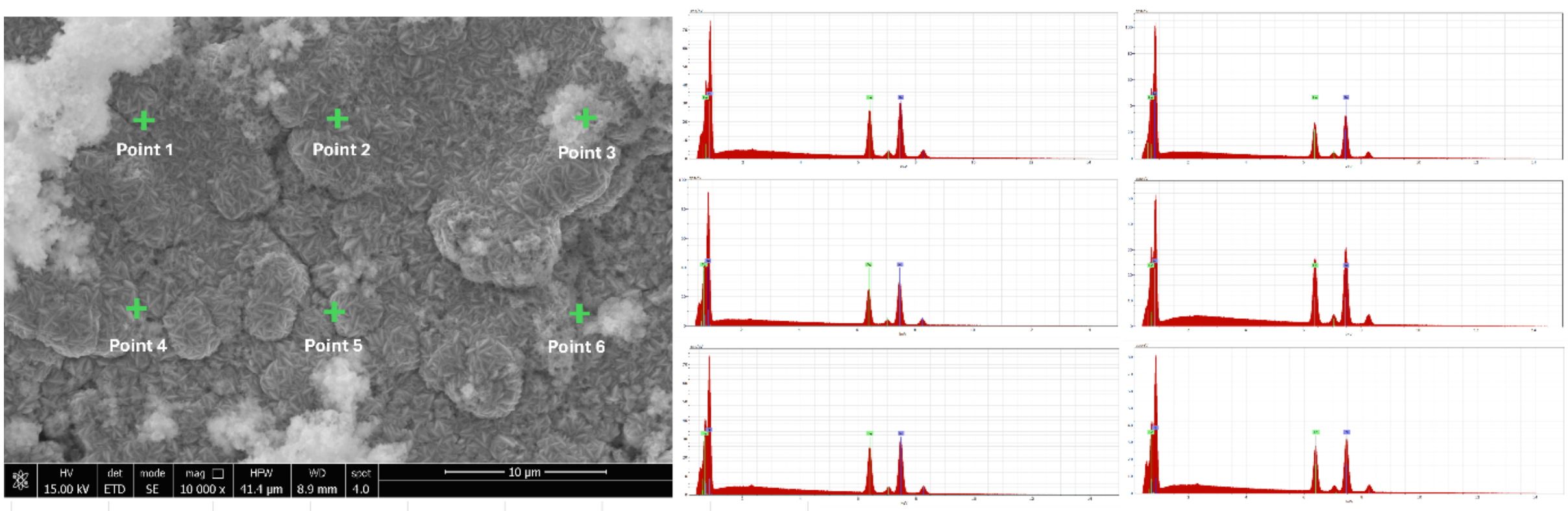

Element	Point 1	Point 2	Point 3	Point 4	Point 5	Point 6	Average
Iron	31.56	31.5	29.17	29.96	30.87	30.24	30.55
Nickel	68.43	68.5	70.82	70.03	69.12	69.75	69.44167

Fig. S3 Nickel and iron distribution for $NiFe(OH)_2$. Elemental distribution of EDS plot of $NiFe(OH)_2$ catalyst synthesised using 2.5 mA cm^{-2} for 500 seconds on polished carbon plate, 0.2376 cm^2 surface area within 18 mM metal sulphate & 25 mM ammonium sulphate.

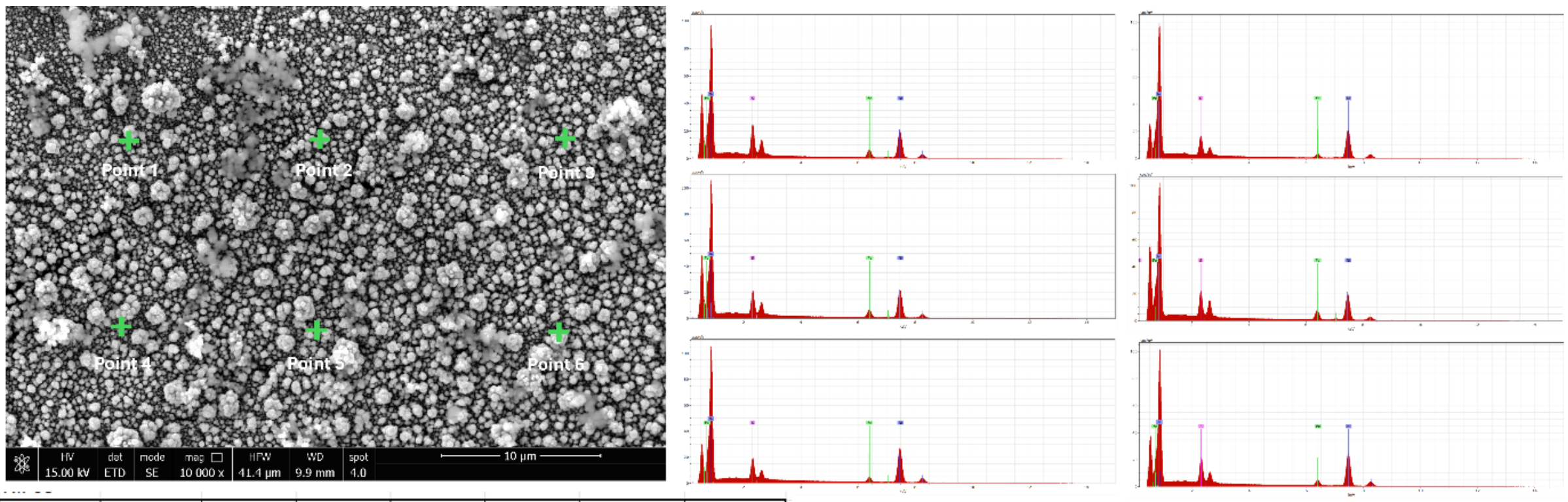

Element	Point 1	Point 2	Point 3	Point 4	Point 5	Point 6	Average
Sulphur	16.99	11.74	13.36	10.96	14.72	11.33	13.18
Iron	11.03	5.43	10.61	5.85	14.2	8.03	9.19
Nickel	71.97	82.82	76.02	83.18	71.07	80.63	77.62

Fig. S4 Nickel, iron and sulphur distribution for NiFe-S. EDS plot of NiFe-S catalyst synthesised using 5 mA cm^{-2} for 2100 seconds on polished carbon plate, 0.2376 cm^{2} surface area within 18 mM metal sulphate & 25 mM ammonium sulphate.

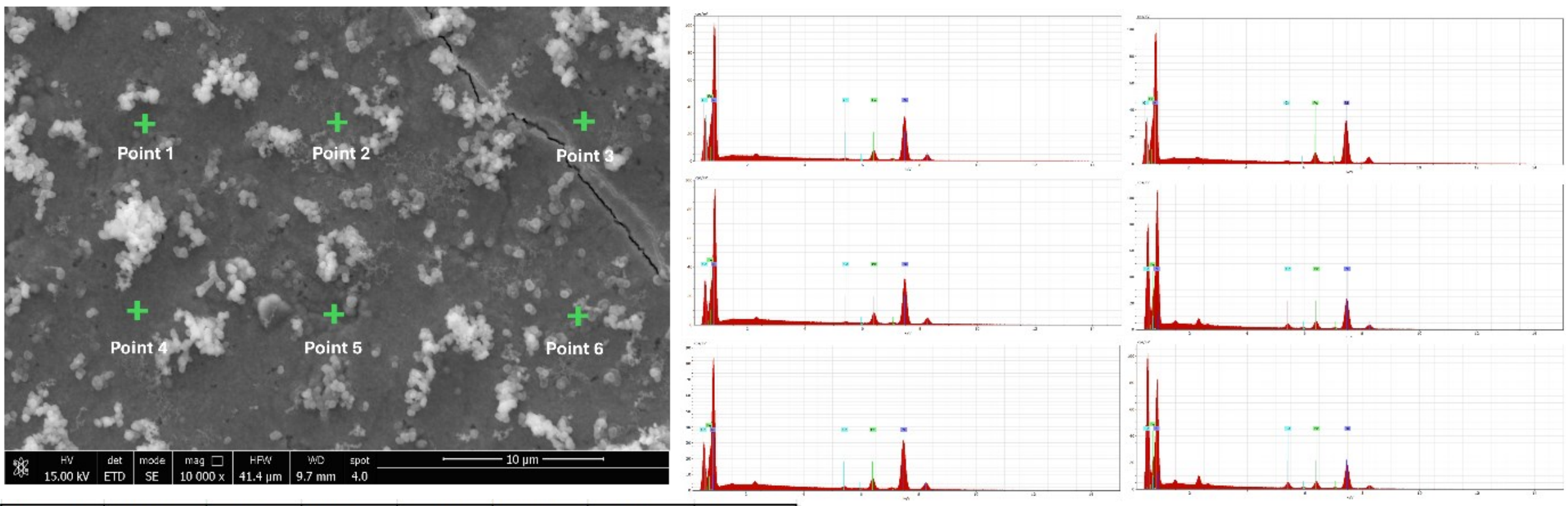

Element	Point 1	Point 2	Point 3	Point 4	Point 5	Point 6	Average
Chromuim	9.65	8.08	6.31	6.69	3.95	6.76	6.91
Iron	12.19	11.42	12.14	12.37	10.83	12.2	11.86
Nickel	78.15	80.49	81.54	80.93	85.21	81.02	81.22

Fig. S5 Nickel, iron and chromium distribution for NiFe-Cr. EDS plot of NiFe-Cr catalyst synthesised using 5 mA cm^{-2} for 2100 seconds on polished carbon plate, 0.2376 cm^{2} surface area within 18 mM metal sulphate & 25 mM ammonium sulphate.

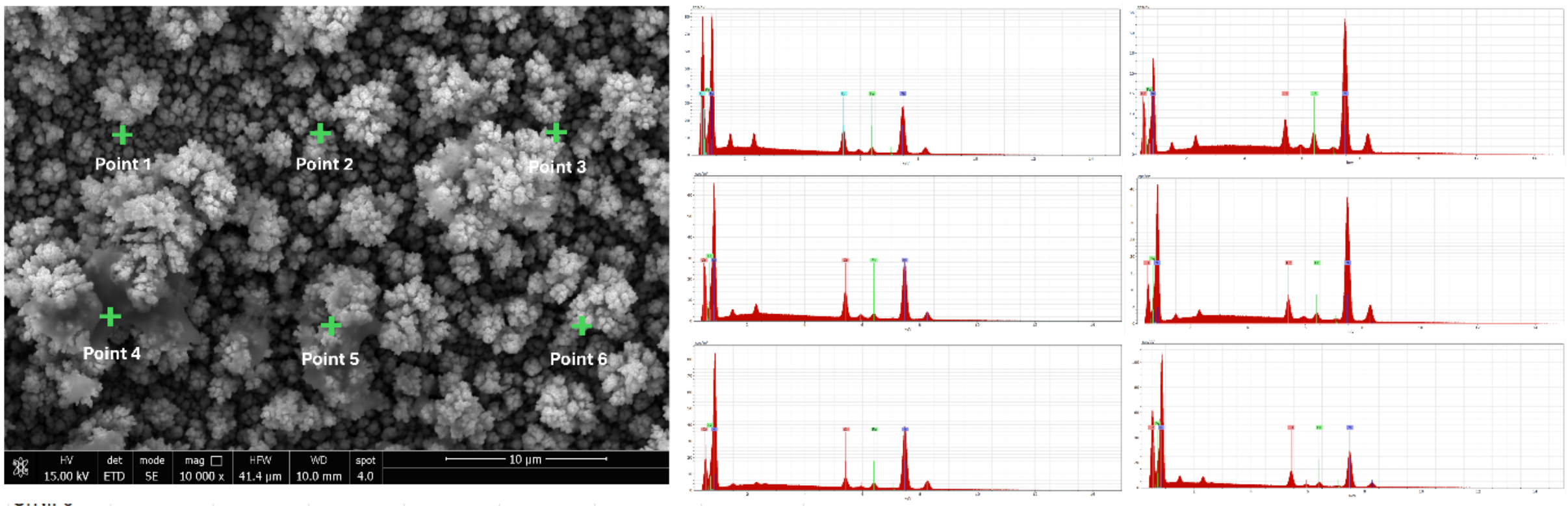

Element	Point 1	Point 2	Point 3	Point 4	Point 5	Point 6	Average
Chromuim	7.94	14.66	13.17	32.14	38.06	14.88	20.14
Iron	6.79	4.02	5.37	5.84	4.69	4.88	5.27
Nickel	85.26	81.31	81.45	62.01	57.24	80.22	74.58

Fig. S6 Nickel, iron and chromium distribution for Cr-NiFe. EDS plot of Cr-NiFe catalyst synthesised using 5 mA cm^{-2} for 2100 seconds on polished carbon plate, 0.2376 cm^2 surface area within 18 mM metal sulphate & 25 mM ammonium sulphate.

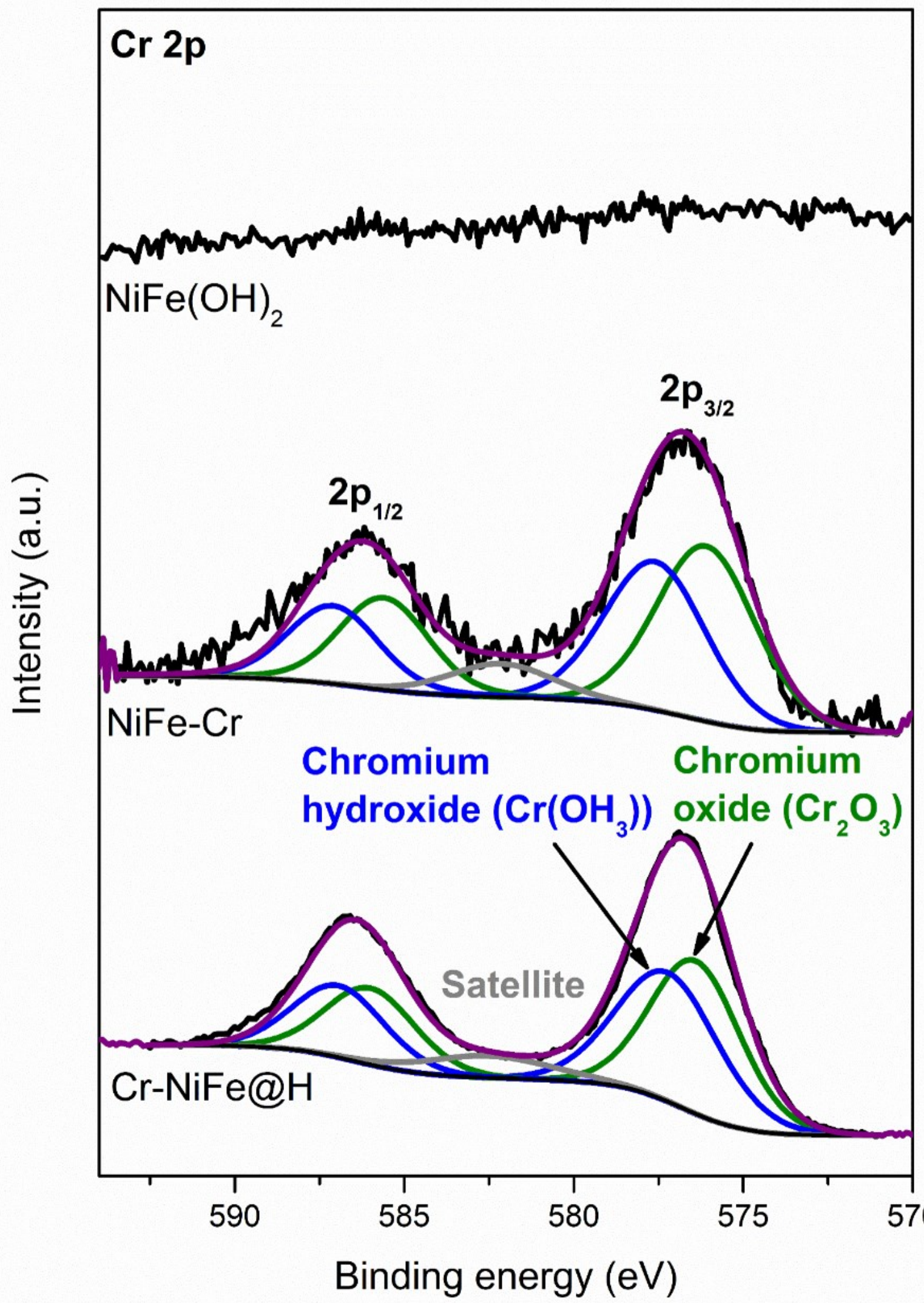

Fig. S7 X-ray photoelectron spectroscopy to identify the valence state of chromium. Chromium XPS 2p region for $NiFe(OH)_2$, NiFe-Cr and Cr-NiFe

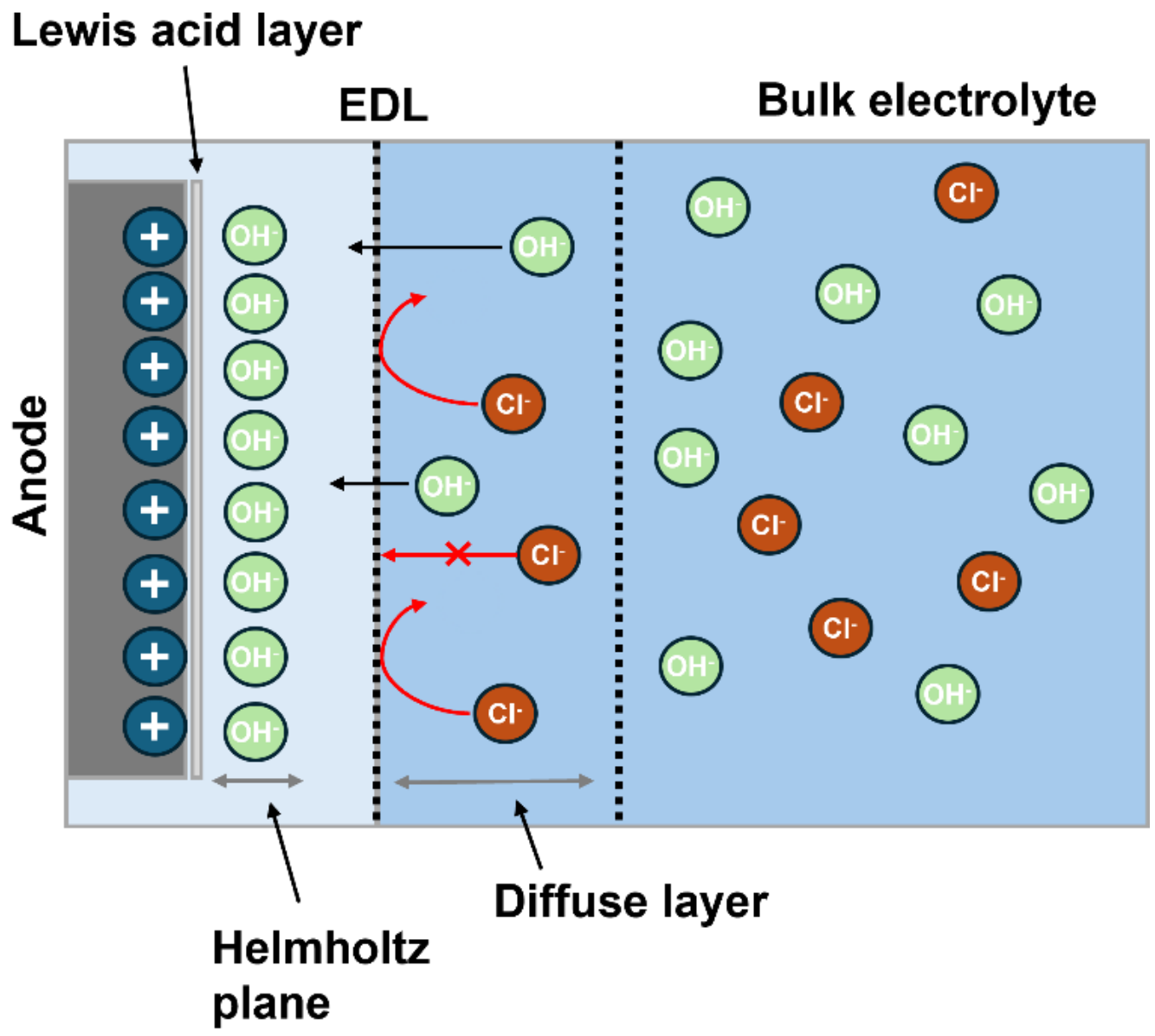

Fig. S8 Schematic for an ion-selective layer on top of the anode during seawater electrolysis. Adapted from ref [1].

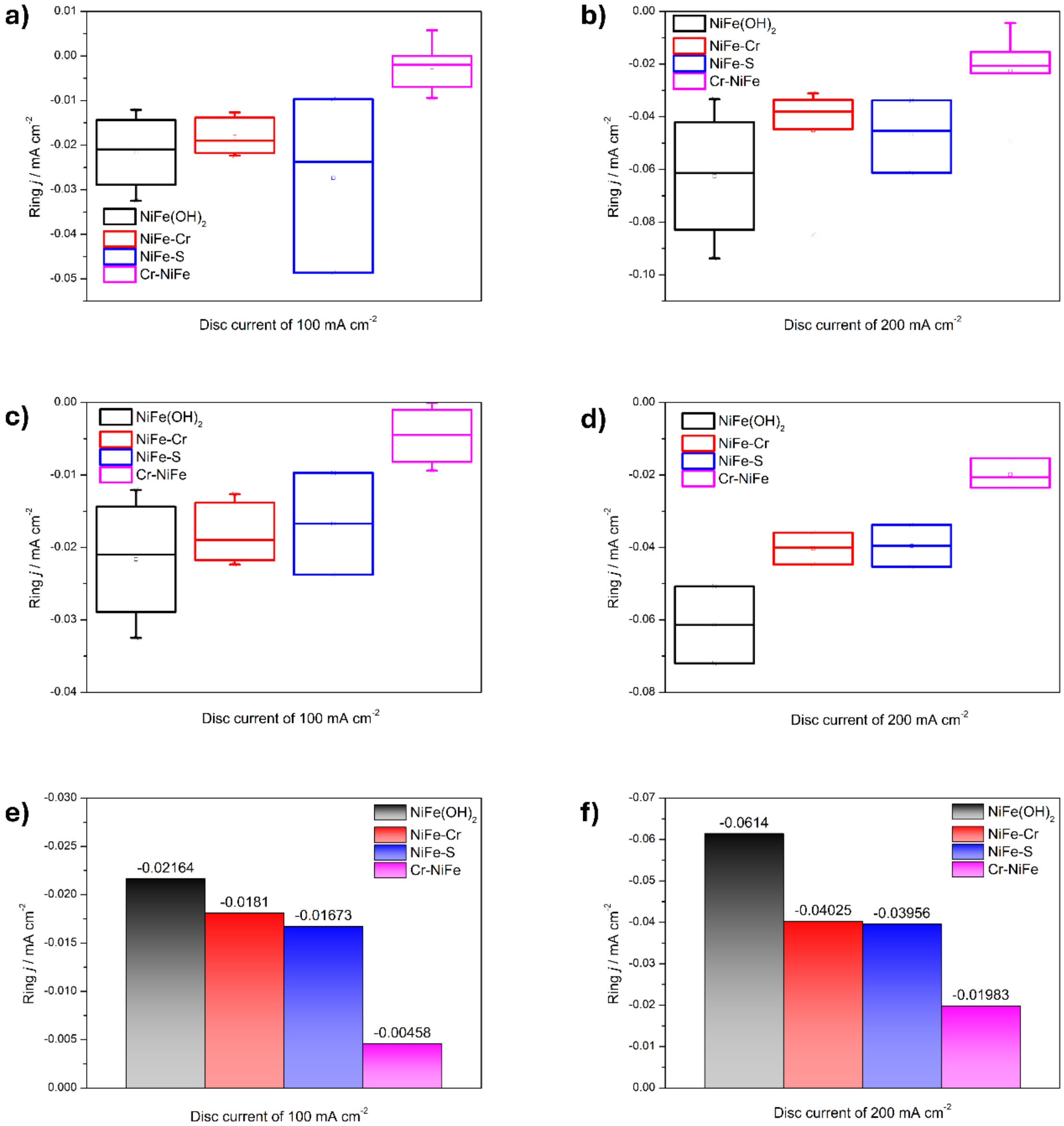

Fig. S9 Statistical reproducibility displayed using box plots of the average ring currents taken over a course of multiple tests. Each experiment was subject to at least 3 repeats a) all ring currents recorded during electrochemical testing at a disc current of 100 mA cm^{-2} b) all ring currents recorded during electrochemical testing at a disc current of 200 mA cm^{-2}. Ring currents recorded during electrochemical testing with anomalous values removed and taking the 3 most consistent data points c) at a disc current of 100 mA cm^{-2} d) at a disc current of 200 mA cm^{-2}.

A display of the reproducibility is highlighted in Fig. S9, where all the ring current density data is collected across electrochemical testing for the catalyst materials and is shown in an average format for simplicity. This data is presented to maximise the

transparency of the methodology and acknowledge flaws. Fig. S9 a-b show the average ring current observed across all tests in simulated seawater with different catalyst materials. The trend observed in the dataset of the main manuscript is consistent with that in these box plots, with the average ring current density decreasing from the highest to the lowest in the following order: $NiFe(OH)_2$>NiFe-Cr>NiFe-S>Cr-NiFe. It is worth highlighting some discrepancies shown in Fig. S9 a-b, specifically the abnormally high average ring current for NiFe-S at a disc current density of 100 mA cm^{-2}, NiFe-Cr at a disc current density of 200 mA cm^{-2}, as well as the large data range of $NiFe(OH)_2$ at a disc current density of 200 mA cm^{-2}. Due to the number of tests conducted and the trends of data collected, we are able to attribute some of these values as anomalies, since the nature of the RRDE experiment within high gas evolution studies will introduce a level of discrepancy over conventional electrochemical testing, due to the complexity of measuring products in-situ, which is explained by many authors.[2–6] As a result of the abnormalities, removing these values (Fig. S9c-d) still yields the same trends observed in the main manuscript and brings the overall averages closer to the values used in the manuscript (Fig. S9e-f). A key point to highlight with these box plots is that the trend in selectivity of the catalysts does not change, and we can say with confidence that the overlayer of chromium has a direct effect on reducing the ring current, which can be linked to hypochlorite production at the disc electrode (Fig. S9e-f). Ultimately, it is acknowledged that this RRDE methodology/protocol is not without limitations; the conclusions drawn in this study can only be explicitly concluded within the materials used.

To gain further insight into the RRDE methodology we have developed, we applied $NiFe(OH)_2$ into a standardised seawater electrolyte (1 M KOH & AbsOcean) using the identical parameters to identify how the ring would respond. Fig. S10 shows the data collected, it is evident that testing in seawater results in a greater disc overpotential for a given disc current density, particularly at lower disc current densities. From Fig. S10b, a ring current can be observed at 50 mA cm^{-2}, where this is not typically seen in the simulated seawater electrolyte. However, at higher disc currents, a lower ring current density is observed for the standardised seawater electrolyte than for the simulated seawater electrolyte (Fig. S10c), we estimate that this is because natural seawater contains numerous ions such as Br^-, SO_4^{2-}, and HCO_3^-, along with organic compounds, which can interfere with ClO^- electrochemically

or chemically. Some of these anions may adsorb onto the electrode, reduce sensitivity, or change the local pH and redox conditions, thereby influencing the identification and reactivity of ClO^-. The most notable ion to complicate this detection is bromide (Br^-) which occurs at a concentration of 0.8 mM in seawater and can be oxidised at similar potentials. This process produces BrO^- or Br_2, which can compete with ClO^- during generation or detection, or form mixed halogen compounds like BrCl that disrupt the electrochemical signal. Additionally, they may react with ClO^- during side reactions.

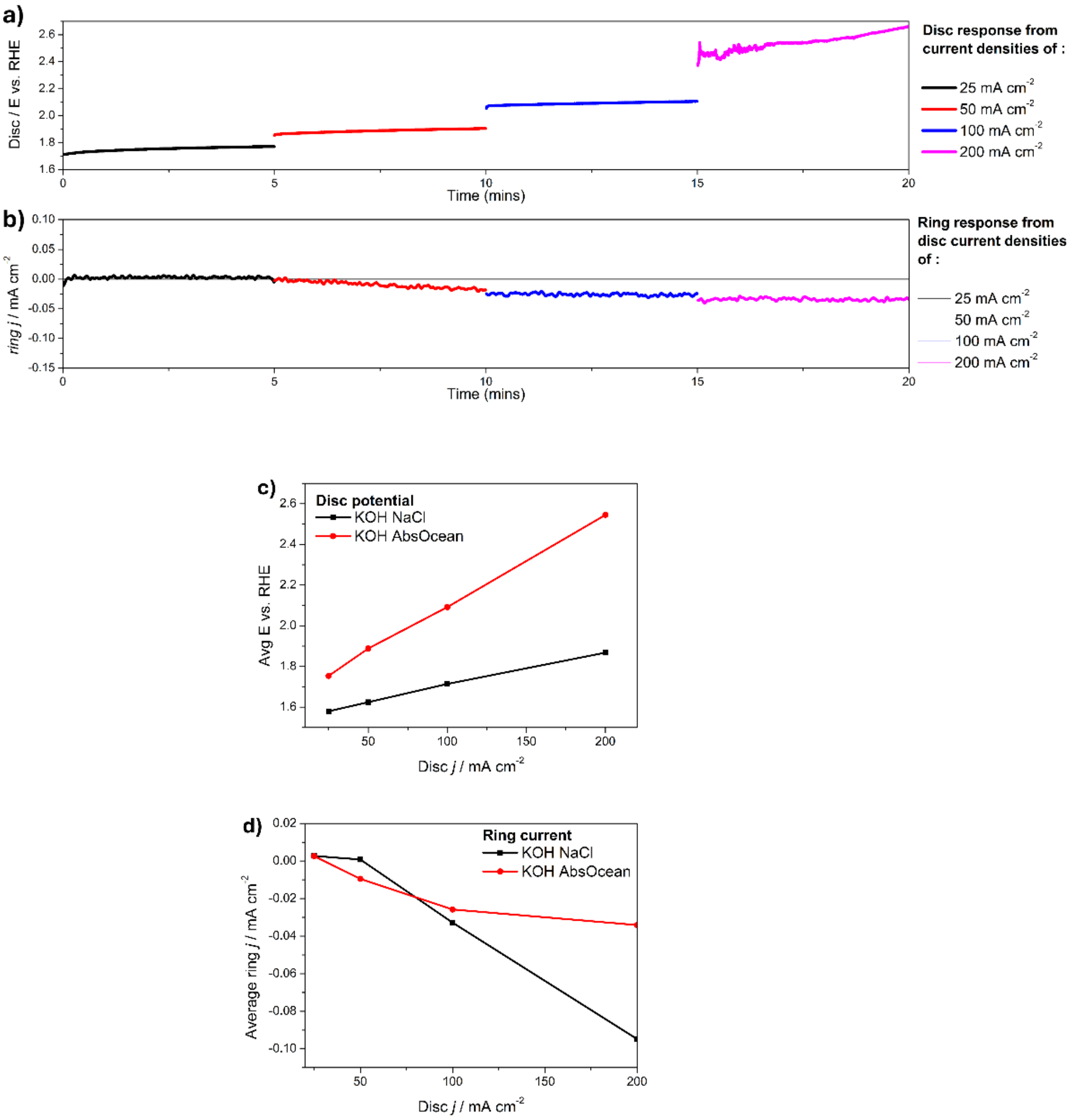

Fig. S10 Experimental methodology applied in alkaline standardised seawater compared to alkaline simulated seawater. $NiFe(OH)_2$ coated disc in 1 M KOH & AbsOcean a) disc potential b) ring current density with respect to different ring current densities. Comparison of c) disc potentials d) ring current densities between 1 M KOH & 0.5 M NaCl and 1 M KOH & AbsOcean.

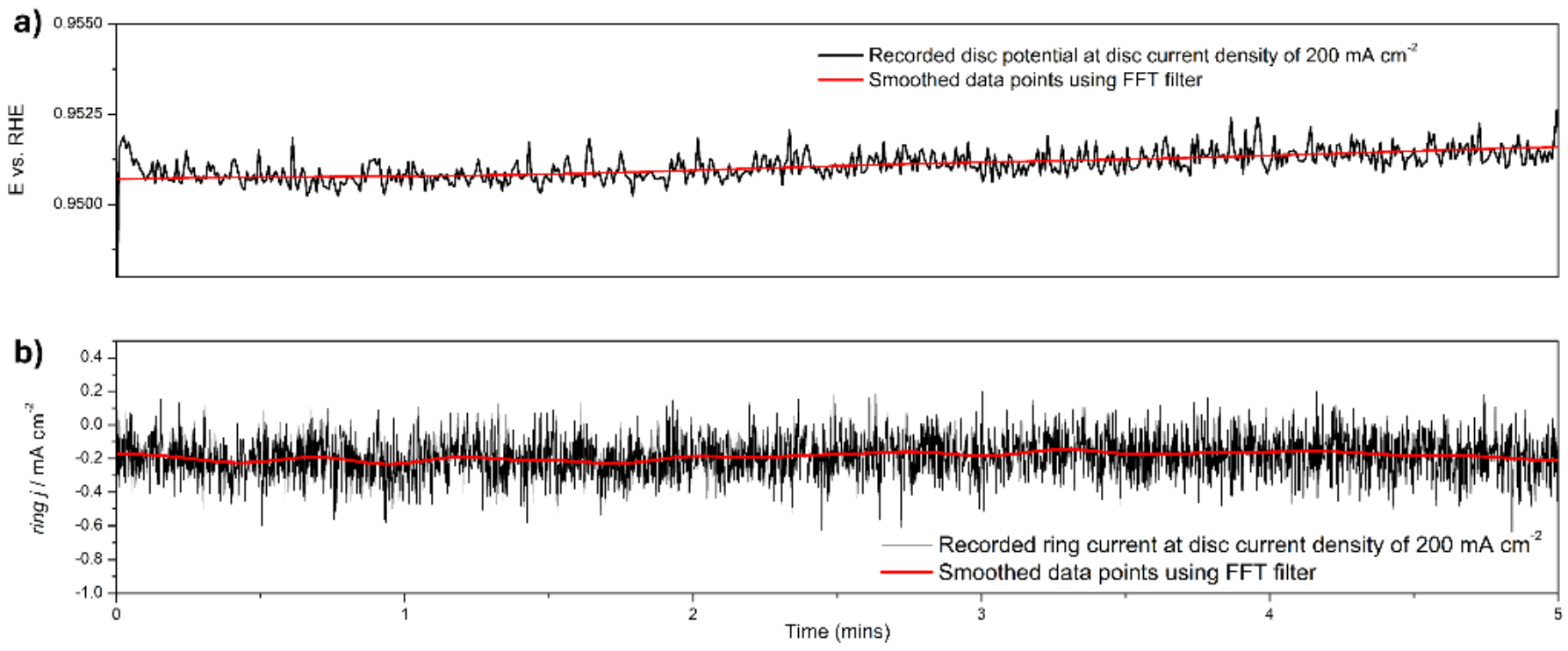

Fig. S11 Treatment of ring and disc raw data. Example of average ring current at 200 mA cm^{-2} in 1 M KOH & 0.5 M NaCl smoothed using FFT filter over 100 points to reduce the effect of noise on the ring current reading.

2. RRDE ClO^- concentration calculations

Data collected during the determination of the hypochlorite sensitive region using RRDE voltammetry, using different concentrations of sodium hypochlorite.

Equation 9: $y = mx + b$

Where:

- y = ring current density (mA cm^{-2})
- m = slope = -0.0438 mA cm^{-2} / mM^{-1} calculated from concentration calibration curve.
- x = NaOCl concentration (mM)
- b= y-intercept = -8.65 μA cm^{-2} calculated from concentration calibration curve.

Table S1 Calculation of localised ClO^- concentrations based on the ring current density at different disk current densities for the different catalysts tested. Calculations are based on the concentrations used to determine the hypochlorite-sensitive region. In Fig. 2c of the manuscript.

	**100 mA cm^{-2}**	**200 mA cm^{-2}**
**$NiFe(OH)_2$**	$-0.0325 = -0.0438x + 0.00865$	$-0.09482 = -0.0438x + 0.00865$

	$-0.0325 - 0.00865 = -0.0438x$<br>$-0.04115 = -0.0438x$<br>$x = \frac{-0.04115}{-0.0438} = 0.939$ mM	$-0.09482 - 0.00865 = -0.0438x$<br>$-0.10347 = -0.0438x$<br>$x = \frac{-0.10347}{-0.0438} = 2.362$ mM
**NiFe-Cr**	$-0.0224 = -0.0438x + 0.00865$<br>$-0.0224 - 0.00865 = -0.0438x$<br>$-0.03105 = -0.0438x$<br>$x = \frac{-0.03105}{-0.0438} = 0.708$ mM	$-0.0401 = -0.0438x + 0.00865$<br>$-0.0401 - 0.00865 = -0.0438x$<br>$-0.04875 = -0.0438x$<br>$x = \frac{-0.04875}{-0.0438} = 1.113$ mM
**NiFe-S**	$-0.0238 = -0.0438x + 0.00865$<br>$-0.0238 - 0.00865 = -0.0438x$<br>$-0.0324 = -0.0438x$<br>$x = \frac{-0.0324}{-0.0438} = 0.740$ mM	$-0.0337 = -0.0438x + 0.00865$<br>$-0.0337 - 0.00865 = -0.0438x$<br>$-0.04235 = -0.0438x$<br>$x = \frac{-0.04245}{-0.0438} = 0.966$ mM
**Cr-NiFe**	$-0.0069 = -0.0438x + 0.00865$<br>$-0.0069 - 0.00865 = -0.0438x$<br>$-0.0155 = -0.0438x$<br>$x = \frac{-0.0155}{-0.0438} = 0.355$ mM	$-0.0207 = -0.0438x + 0.00865$<br>$-0.0207 - 0.00865 = -0.0438x$<br>$-0.02935 = -0.0438x$<br>$x = \frac{-0.02405}{-0.0438} = 0.670$ mM

The standard error of the y-intercept (±0.00626) and slope should be considered when determining confidence in calculated concentrations, particularly at lower intensities where noise becomes more significant.

Formula for rates of ClO⁻ formation:

Equation 10: Rate of COR = $\frac{\frac{I_R}{N}}{n \times F \times Vol} = mmol\ s^{-1}$

Equation 11: Rate of COR = $\frac{\left(\frac{\frac{I_R}{N}}{n \times F}\right)}{A_D} = mmol\ cm^{-2}\ s^{-1}$

I_r = ring current (mA)

N = experimental collection efficiency (38.52%)

n = number of electrons in reaction

F = Faraday's constant (96,485 C/mol)

A_D = Disc surface area (cm^2)

Vol = Volume of electrolyte (100 ml in a 150 ml cell)

Example calculation for localised ClO^- concentration to reach 1 mM dm^{-3} in 100 ml electrolyte in the bulk solution: *$NiFe(OH)_2$ r*ate of COR *at 200 mA cm^{-2}*

$$\text{Equation 12: } = \left(\frac{1\ mM}{3.006 \times 10^{-6}\ mmol\ s{-1}}\right) = 332{,}667.99\ secs$$

$$\text{Equation 13: } \frac{332{,}667.99}{60} = 5{,}544.46\ mins$$

$$\text{Equation 14: } \frac{5{,}544.46}{60} = 92.40\ hrs$$

$$\text{Equation 15: } \frac{92.4}{24} = 3.85\ days$$

Based on the calculations for $NiFe(OH)_2$ at 200 mA cm^{-2} in 1 M KOH & 0.5 M NaCl, it would take 3.85 days to reach 1 mM dm^{-3} of ClO^- in 100 ml of electrolyte in the bulk solution. It is essential to note that this period can be influenced by a range of factors, including the rate of COR production and the tendency to ramp up or down, which depends on the electrode surface and the reactions occurring. Degradation of the catalyst materials at this concentration will further affect the concentration, due to the corrosive nature of ClO^-, and greater ClO^- will result in greater catalyst degradation. Furthermore, this rate is necessary to achieve the bulk concentration; similarly, the localised concentration is also more critical in terms of electrode stability and corrosion due to the diffusion of unwanted ions.

Table S2 Rates of chloride oxidation reaction (COR) between disc and ring during electrochemical testing in 1 M KOH & 0.5 M NaCl based on ring current densities.

**Catalyst**	**Rate of ClOR(mmol s^{-1})**		**Rate of ClOR(mmol cm^{-2} s^{-1})**	
	**At 100 mA cm^{-2}**	**At 200 mA cm^{-2}**	**At 100 mA cm^{-2}**	**At 200 mA cm^{-2}**
**$NiFe(OH)_2$**	1.030×10^{-6}	3.006×10^{-6}	4.336×10^{-6}	1.265×10^{-5}
**NiFe-Cr**	7.100×10^{-7}	1.271×10^{-6}	2.988×10^{-6}	5.348×10^{-6}
**NiFe-S**	7.544×10^{-7}	1.070×10^{-6}	3.175×10^{-6}	4.503×10^{-6}
**Cr-NiFe**	2.187×10^{-7}	6.562×10^{-7}	9.205×10^{-7}	2.762×10^{-6}

Simplified Nernst equation for converting to RHE

$$\text{Equation 16: } \mathrm{E_{RHE}} = E_{Hg/HgO} + \left(2.303 \times \frac{RT}{F}\right) \times \mathrm{pH} + E^0_{Hg/HgO}$$

R is the ideal gas constant (8.314 J K^{-1} mol^{-1}), T is the temperature (298 K), F is the Faraday constant (96485 C mol^{-1}), and $E^0_{Hg/HgO}$ is the standard potential of the Hg/HgO electrode (0.0983 V vs. standard hydrogen electrode), pH of the electrolytes was ˜14. All potentials are quoted vs. RHE unless otherwise stated.

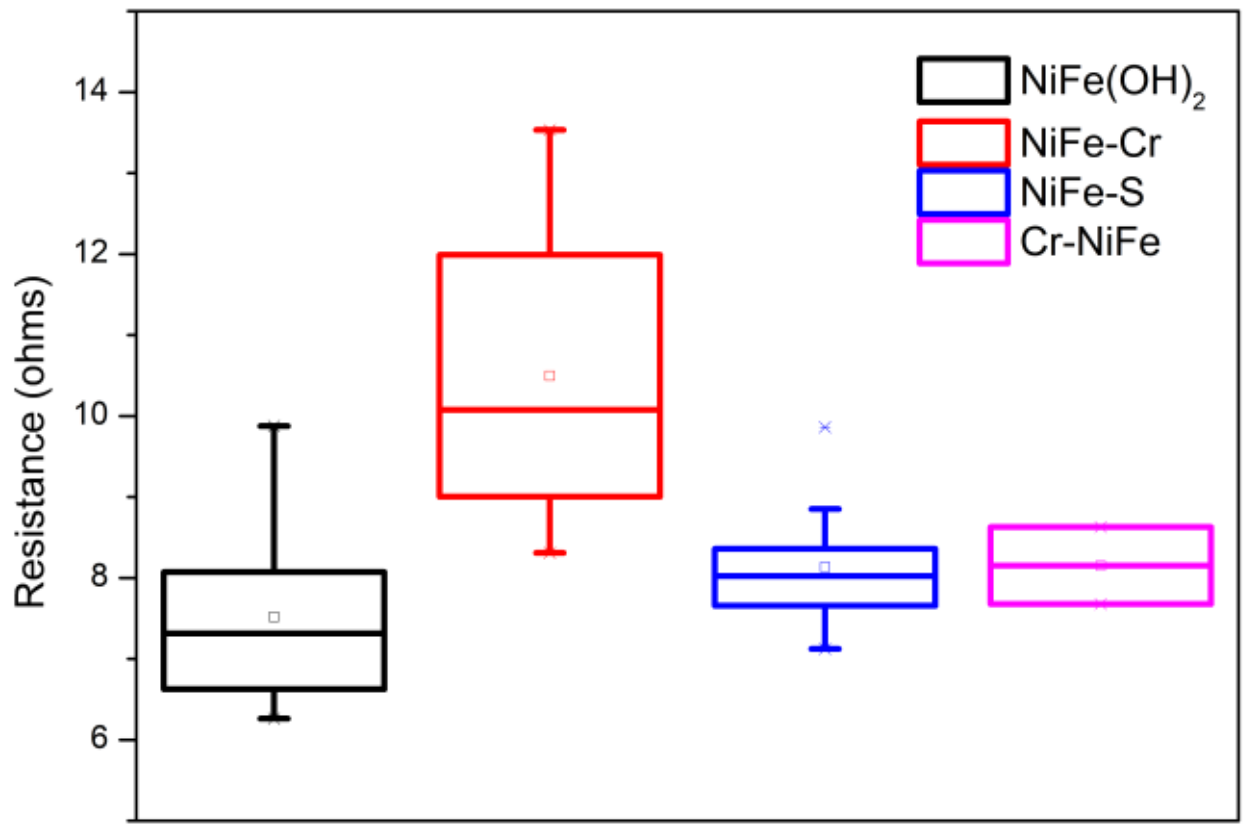

Fig. S12 Resistance values obtained within 1 M KOH and 1 M KOH & 0.5 M NaCl, pH 14 at 298 K for all catalyst materials using the current interrupt method.

References

1 J. Guo, Y. Zheng, Z. Hu, C. Zheng, J. Mao, K. Du, M. Jaroniec, S.-Z. Qiao and T. Ling, *Nat. Energy*, DOI:10.1038/s41560-023-01195-x.

2 J. G. Vos and M. T. M. Koper, *Journal of Electroanalytical Chemistry*, 2018, 819, 260–268.

3 J. G. Vos and M. T. M. Koper, *Journal of Electroanalytical Chemistry*, 2019, 850, 113363.

4 H. Vogt, *Electrochim. Acta*, 2012, 78, 183–187.

5 H. Vogt and R. J. Balzer, *Electrochim. Acta*, 2005, 50, 2073–2079.

6 I. S. Filimonenkov, S. Ya. Istomin, E. V. Antipov, G. A. Tsirlina and E. R. Savinova, *Electrochim. Acta*, 2018, 286, 304–312.
