# Supplementary material for: Assessing hypochlorite selectivity of corrosion resistance catalysts for alkaline seawater splitting
Source: Nat Commun. 2026 Jul 8;17:8089. doi: 10.1038/s41467-026-75110-9 (PMC13458344; doi:10.1038/s41467-026-75110-9)
Supplement: Supplementary file 2 [file 41467_2026_75110_MOESM2_ESM.pdf]

nature portfolio

# Peer Review File

# Assessing hypochlorite selectivity of corrosion resistance OER electrocatalysts for alkaline seawater splitting using a rotating ring-disc electrode

Corresponding Author: Professor Xiaohong Li

**This file contains all reviewer reports in order by version, followed by all author rebuttals in order by version.**

Version 0:

Reviewer comments:

Reviewer #1

(Remarks to the Author)
This manuscript reports a comparative study of electrostatic repulsion and ion-selective strategies for suppressing Cl- oxidation during alkaline seawater electrolysis, using an RRDE-based methodology to quantify ClO- formation. The work is interesting and the use of RRDE for in situ selectivity analysis is potentially valuable. However, several substantial issues should be addressed in presentation, structural characterization, and mechanistic investigations to strengthen the novelty and data reliability of the study:

Reasons:
General:
1) Several figures are insufficiently clear, with low resolution, small fonts, and indistinct features.
2) Widespread inconsistencies between text, figures, equations and captions. The figures descriptions of in the main text frequently do not follow the actual order of panels shown in the figures, including the figures shown in supporting information. e.g. Figure 2, Figure 5, Figures S2-S7; Equation 3 is referenced on page 13, line 10, but the equation shown does not match the described reaction.
3) Many other mistakes: -0.461437 µA cm-2, 1.72 VRHE. A thorough check of all text is essential.
Characterization:
The manuscript discusses four critical catalyst systems: NiFe(OH)2, NiFeCr, NiFeS, and CrNiFe. However, their structural characterization is limited to SEM, EDS, and XPS, which is insufficient to support the structural and mechanistic claims made. Given that the proposed mechanism critically relies on surface vs bulk Cr distribution, ion-selective adsorption, advanced structural characterizations, like XRD/TEM are necessary.
2) The suppression of ClO- formation is attributed to preferential OH- adsorption based on HSAB theory, but no direct evidence of corresponding OH- or Cl- adsorption is provided. Some in situ characterizations, like Raman under COR-relevant potentials can be used to validate changes in surface adsorption behaviour.
More evidence is needed to adequately solve the mechanistic concern:
1) Bubble formation at high current densities (≥100-200 mA cm-2) might significantly perturb mass transport and thus affect collection efficiency. If this effect can be assessed? e.g. change rotation speeds to see the ring current values?
2) The use of a calibration curve derived from homogeneous bulk ClO- solutions to estimate localized, transient ClO- concentrations generated at the disc brings uncertainty.
3) It remains unclear whether lower ClO- production arises from selectivity effects or from reduced accessibility of COR-active sites. Given the substantial morphological differences among the catalysts, as shown in the SEM images, ECSA measurements are required to normalize the data to electrochemically active surface area or intrinsic activity.

Reviewer #2

(Remarks to the Author)
The author used the rotating ring disk electrode (RRDE) device to compare two strategies for inhibiting COR and CER, independently detecting the formation of hypochlorite at the disk in 1 M KOH and 0.5 M NaCl (simulated seawater) solutions. The results indicated that in alkaline seawater, the ion-selective strategy was more effective in reducing the formation of hypochlorite. However, the electrostatic repulsion strategy could improve selectivity to reduce the formation of hypochlorite

in terms of electrochemical performance, but its performance slightly declined. This insight will greatly enhance the efficiency and development level of oxygen evolution reaction electrocatalysts in seawater electrolysis, as it can simplify the design of oxygen evolution reaction catalysts. However, the manuscript still has many issues that require attention. It is recommended for major revision.

1.The title is misleading and may give the impression that the authors have developed a ring-disc electrode for evaluating hypochlorite selectivity. The authors need to rewrite the title carefully.

2.The abstract section is too long. The authors need to condense and optimize it.

3.In the Graphical abstract, there is too much text and it is too small, making it difficult to read.

4.The author should pay attention to the reliability of the evidence. Some of the content goes against basic common sense. For instance, the sentence "However, creating sustainable hydrogen
remains expensive, as most production methods currently rely on fossil fuels." is not very reasonable. It is well-known that the current price of green hydrogen is much higher than that of grey hydrogen and blue hydrogen. There are many such questionable statements like this, and the author needs to pay attention to them.At the same time, the author's writing in the introduction section is overly obscure and full of leaps in logic, making it extremely difficult to understand. The author will discuss the significance of hydrogen for a while, then write about water scarcity, and also about the Australian deserts, and so on. It is suggested to focus on one main point and develop it accordingly.

5.CER and COR exhibit better inhibitory effects. However, it is worth noting that from the physical property characterization results of the manuscript, the surfaces of these catalysts are not the result of normalization. As shown in the scanning electron microscope test results in Figure 2, the particles after Cr doping have significantly increased, which will significantly increase their active sites. Then, to what extent is the author's demonstrated advantage of Cr derived from the increase in specific surface area? The author needs to normalize the relevant data using the specific surface area method and the electrochemical surface area method.

6.All the statistical data in the manuscript are derived from a single one-time test result. Due to the significant systematic error in electrochemical testing, the author needs to increase the number of tests for each data point and include the error range in all the statistical graphs. This thereby enhances the reliability of the data.

7.Due to the significant differences in the surface of the samples, in order to ensure the accuracy and rationality of the final conclusion, it is suggested that the author add higher magnification scanning electron microscope images or transmission electron microscope images to present more detailed information.

Reviewer #3

(Remarks to the Author)

1. Introduction is overly long and reads like a literature review rather than a focused motivation for the present study.
2. In experimental section, the authors report the synthesis of four catalysts ($NiFe(OH)_2$, NiFeS, NiFeCr, and CrNiFe) using electrodeposition. Forming phase-pure materials via electrodeposition typically requires careful optimization, but no systematic optimization process is described. This raises concerns regarding the reproducibility and phase purity of the synthesized catalysts.
3. On page 14 (lines 17–22), the authors should clarify whether the synthesized material is $NiFeS_x$ or sulphur-doped $NiFe(OH)_2$. Identification of the material is provided. Additional structural characterization, such as XRD, is recommended to confirm the phase and composition of the synthesized material.
4. TEM analysis is recommended to explain the effect of chromium modification on $NiFe(OH)_2$. Although XPS suggests the oxidation states in the NiFeCr and CrNiFe samples, complementary TEM and XRD analyses can provide more insight into chromium distribution, phase formation, and its structural position in $NiFe(OH)_2$.
5. Figures 5e and 6a show different current values despite apparently using the same catalysts and experimental conditions. The authors should clarify the reason for this discrepancy.
6. The authors should explain the origin of the performance difference between NiFeCr and CrNiFe catalysts, as both contain chromium species. The limited characterization makes it difficult to rationalize their different electrochemical and corrosion-resistance behaviors. In addition to chromium content, critical factors such as chromium distribution, oxidation state, phase composition, crystallinity, defect density, surface composition, and microstructural differences may influence corrosion resistance and should be considered.

Major revision is required before the work is considered for publication.

Version 1:

Reviewer comments:

Reviewer #1

(Remarks to the Author)

The authors have adressed my concerns now, and this work can be published now.

Reviewer #2

(Remarks to the Author)

I have completed my review of this manuscript. Overall, I find it to be a well-written and scientifically sound piece of work. The research question is clear, themethodology is appropriate, the results are presented clearly, and the conclusions are supported by the data.The manuscript fits well within the scope of Nature Communications.

Therefore, I am pleased to recommend that this manuscript be accepted for publication in its current form.

Reviewer #3

(Remarks to the Author)
publish as is

**Open Access** This Peer Review File is licensed under a Creative Commons Attribution 4.0 International License, which permits use, sharing, adaptation, distribution and reproduction in any medium or format, as long as you give appropriate credit to the original author(s) and the source, provide a link to the Creative Commons license, and indicate if changes were made.
In cases where reviewers are anonymous, credit should be given to 'Anonymous Referee' and the source.
The images or other third party material in this Peer Review File are included in the article's Creative Commons license, unless indicated otherwise in a credit line to the material. If material is not included in the article's Creative Commons license and your intended use is not permitted by statutory regulation or exceeds the permitted use, you will need to obtain permission directly from the copyright holder.
To view a copy of this license, visit https://creativecommons.org/licenses/by/4.0/

**<u>Decision on Nature Communications manuscript NCOMMS-25-97645</u>**

**REVIEWER COMMENTS**

**Reviewer #1 (Remarks to the Author):**

This manuscript reports a comparative study of electrostatic repulsion and ion-selective strategies for suppressing Cl- oxidation during alkaline seawater electrolysis, using an RRDE-based methodology to quantify ClO- formation. The work is interesting and the use of RRDE for in situ selectivity analysis is potentially valuable. However, several substantial issues should be addressed in presentation, structural characterization, and mechanistic investigations to strengthen the novelty and data reliability of the study:

We appreciate the positive feedback provided by the reviewer, and we hope that the revised manuscript will address the concerns raised.

**Reasons:**

**General:**

1) Several figures are insufficiently clear, with low resolution, small fonts, and indistinct features.

Thank you for pointing this out. The reviewer is correct, and we have improved the quality of the figures by increasing font sizes and reintroducing the images in the manuscript as higher-resolution PNGs.

2) Widespread inconsistencies between text, figures, equations and captions. The figures descriptions of in the main text frequently do not follow the actual order of panels shown in the figures, including the figures shown in supporting information. e.g. Figure 2, Figure 5, Figures S2-S7; Equation 3 is referenced on page 13, line 10, but the equation shown does not match the described reaction. Many other mistakes: -0.461437 µA cm-2, 1.72 VRHE. A thorough check of all text is essential.

We thank the reviewer for pointing these out; the reviewer is correct, and we have carefully reviewed the manuscript to correct any identified errors. We hope that the updated manuscript satisfies the reviewer.

**Characterization:**

1) The manuscript discusses four critical catalyst systems: NiFe(OH)2, NiFeCr, NiFeS, and CrNiFe. However, their structural characterization is limited to SEM, EDS, and XPS, which is insufficient to support the structural and mechanistic claims made. Given that the proposed mechanism critically relies on surface vs bulk Cr distribution, ion-selective adsorption, advanced structural characterizations, like XRD/TEM are necessary.

We thank the reviewer for highlighting this critical point. The reviewer is right XRD and TEM are very useful techniques to provide greater information about the catalyst materials. These techniques were not included in the initial manuscript because we had a separate paper focused on the in-depth characterisation of the materials and their intrinsic properties. This manuscript aims to complement the previous manuscript by focusing on the variations in selectivity introduced by the experimental methodology. Furthermore, XRD did not reveal any additional information about the catalysts due to the materials' amorphous nature. TEM did provide some highlights of the variations in particle distribution; thus, we have included them below for the reviewer's clarity. However, as this data is included with another journal, it is not included in the manuscript here.

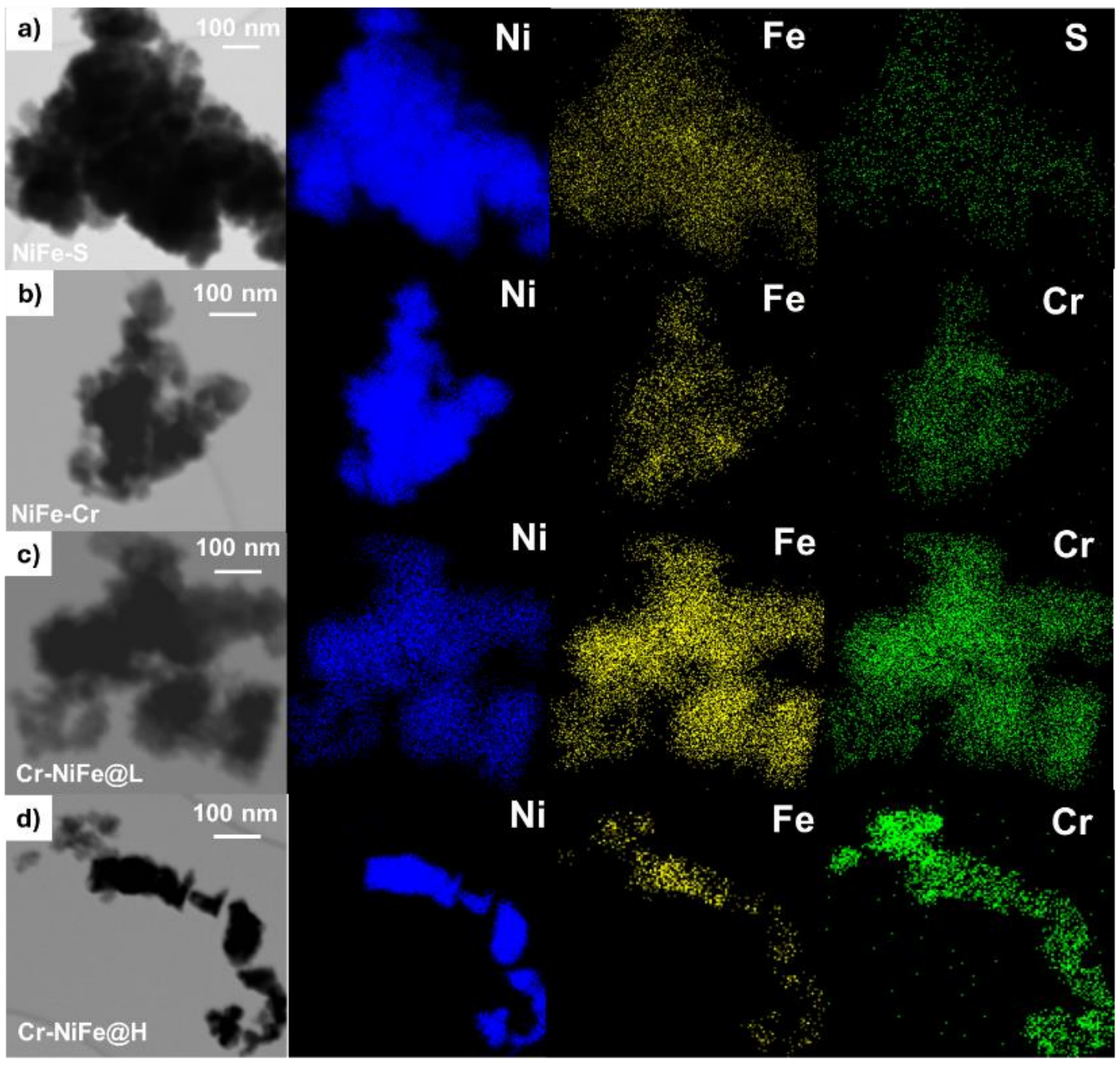


Figure 1 Transmission electron microscopy energy dispersive spectroscopy (TEM-EDS) mapping of corrosion-resistant catalysts on carbon plate deposited from 18 mM metal sulphate & 25 mM ammonium sulphate using -50 mA $cm^{-2}$ for 210s using a magnetic stirrer at 500 rpm a) NiFe-S b) NiFe-Cr c) Cr-NiFe@L d) Cr-NiFe@H.

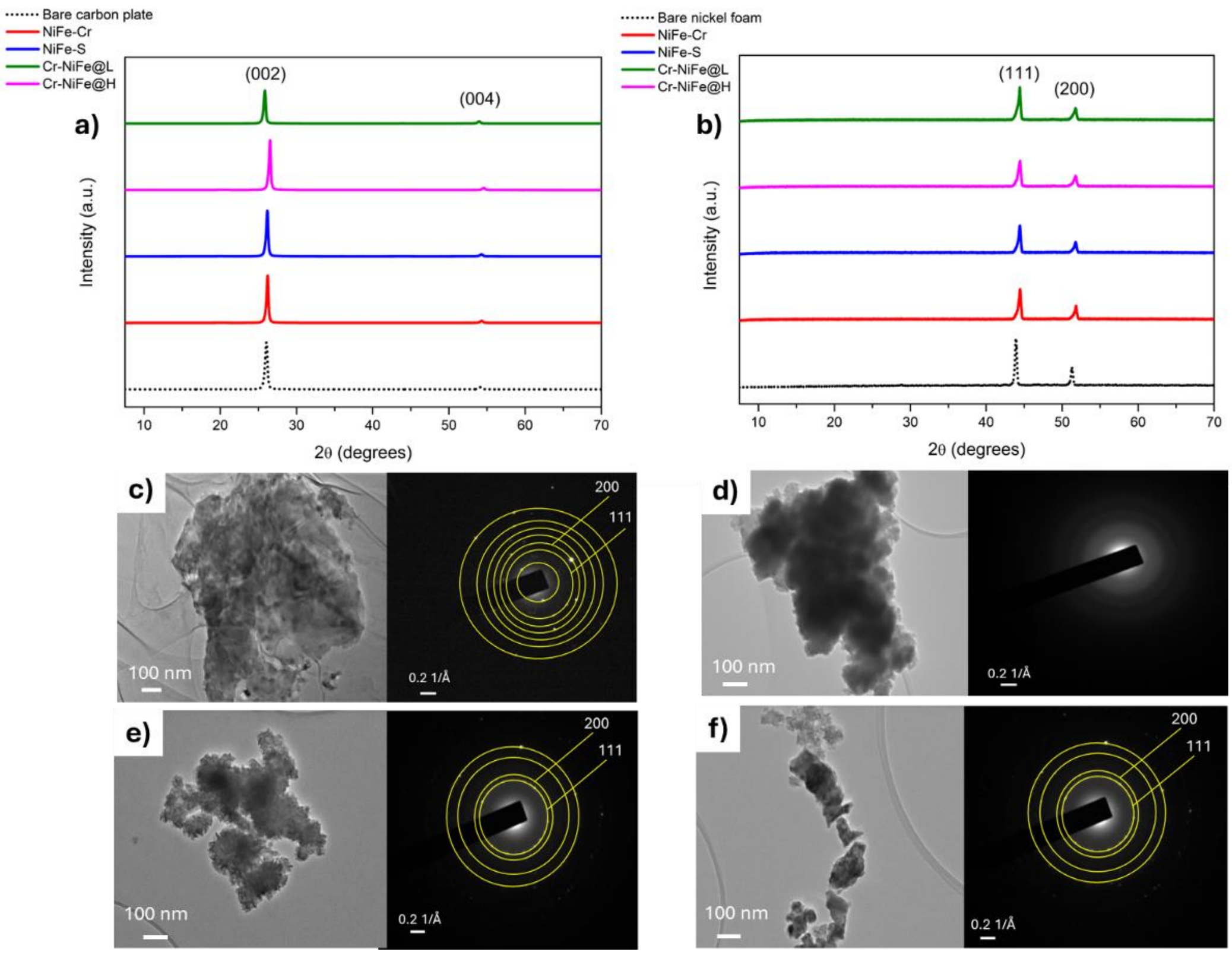


Figure 2 XRD patterns of deposited corrosion-resistant coatings on a) 1 $cm^2$ of carbon plate using -50 mA $cm^{-2}$ for 210s b) 1 $cm^2$ of nickel foam using -50 mA $cm^{-2}$ for 210s. Low-magnification TEM image and SAED ring diffraction pattern of c) NiFe-Cr d) NiFe-S e) Cr-NiFe@L f) Cr-NiFe@H.

Figure 2 a & b show no peaks associated with the catalyst coatings, only peaks from the substrate. Distinct diffraction peaks for raw graphite; an intense crystalline peak is observed at a 2θ of 26.4˚, with a lattice spacing of ˜3.4 Å, which links to the diffraction plane of graphite (002). A smaller peak is observed at a 2θ of 54.5°, corresponding to the (004) diffraction plane.[1] Initially it was anticipated that the carbon substrate (Figure 2a), due to its high crystallinity, drowned out any catalyst features, yet no catalyst features were observed, still, this was later confirmed by using a similarly crystalline NF substrate (Figure 2b), leading us to the conclusion that all the deposits are amorphous. The high-intensity peaks of the coated NF substrate pattern at 44.5° and 52° correspond to metallic Ni (COD 2100646), originating from the metal NF substrate (Figure 2b), with a calculated lattice spacing of approx. 2 Å and 1.7 Å, respectively, which relate to planes of (111) and (200).[2] Furthermore, the peak intensity of NF decreases upon catalyst application, further confirming the catalyst's amorphous nature. Since a significant portion of the crystallinity arises from the metallic substrate, we conclude that the catalyst is primarily amorphous, as confirmed in our previous work.[1] Amorphous materials lack defined crystalline structures and, accordingly, they provide structural flexibility, increased ECSA, and improved stability.[3–5]

TEM analysis was performed to further investigate the crystallinity of the deposit and identify any short-range nanometer-scale crystalline features for the catalyst materials (Figure 2 c-f). The analysis shows TEM micrographs of the various catalysts, along with selected area electron diffraction (SAED) patterns. From the SAEDs, it is evident that some dotted ring patterns were observed, indicating minor crystalline areas across all Cr containing samples, whilst NiFe-S displayed solid rings, which suggests it remains completely amorphous. From the dotted ring patterns in the SAEDs, interplanar d-spacing can be determined from the SAEDs using Bragg's law and is related to the Bragg angle. The crystalline regions with a lower Bragg angle (closer to the centre) in the SAEDs correspond to metallic species, with spacings of approximately 2.0 Å and 1.7 Å, related to the standard diffraction planes (111) and (200) for Ni metal. [1,6–8] It is generally understood that the Cr doped catalyst catalysts are predominantly amorphous, with some crystalline features linked to the mixed metal deposition that results from the reduction of Ni and Fe during cathodic electrodeposition. This is supported by the fact that samples were prepared on a carbon plate and the deposited materials were scraped off, emphasising the Ni observed here is from the catalyst materials, not the substrate. Notably, NiFe-S has a completely amorphous structure, which is hypothesised to be due to two possible reasons. Firstly, the lower overall metal ratio in the electrodeposition solution reduces the amount of divalent Ni and Fe available for reduction. Secondly, thiourea acts as a complexing agent, binding with metal ions. In this context, $Ni^{2+}$ ions can coordinate with thiourea, thereby forming stable surface complexes that inhibit further reduction of these ions.[9]

2) The suppression of ClO- formation is attributed to preferential OH- adsorption based on HSAB theory, but no direct evidence of corresponding OH- or Cl- adsorption is provided. Some in situ characterisations, like Raman under COR-relevant potentials, can be used to validate changes in surface adsorption behaviour.

We sincerely thank the reviewer for the valuable suggestion to include in situ characterisation. We fully agree that such an experiment would provide additional insight and would further strengthen the work by confirming the statements within the manuscript. However, due to the significant cost and time required to conduct this additional work, it is beyond the scope of the current manuscript. We consider this a valuable direction for future work and would recommend it in follow-up studies.

More evidence is needed to adequately solve the mechanistic concern:

1) Bubble formation at high current densities (≥100-200 mA cm-2) might significantly perturb mass transport and thus affect collection efficiency. If this effect can be assessed? e.g. change rotation speeds to see the ring current values?

The reviewer highlights a really critical point here. We have tried to address this comment as accurately as possible. We have included an updated description of this in the manuscript, which can be found on pages 26 and 27, lines 19 to 24 and 1 to 2. We have also updated some figures to further support our discussion, namely Fig. S11 a-b.

But we have included the description below here for ease of access for the reviewer. We hope this can increase the confidence of the reviewer in the data provided in the manuscript:

"It is worth noting that due to the high disc current density applied, some noise is observed (Fig. S11 a-b) across the ring and disc data from the significant gas evolution. The noise is attributed to bubbles formed at the disc that disperse radially. Bubble transport is observed not to perturb the ring current since a steady state current is achieved (Fig. S11 b); a more fractured and highly variable ring current would be observed across the 5-minute period otherwise, since the bubbles would block ClO- detection at the ring either completely or sporadically."

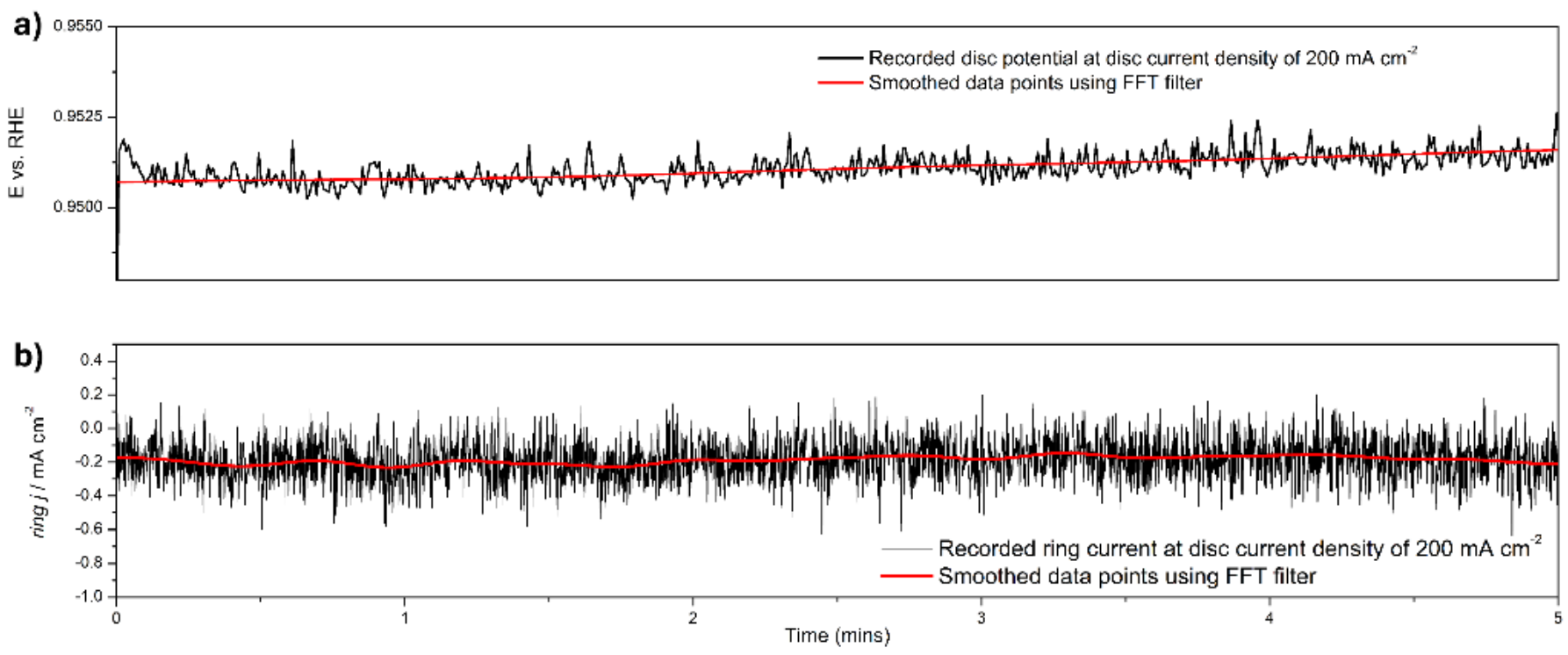


Fig. S11 Example of average ring current at 200 mA cm-2 in 1 M KOH & 0.5 M NaCl smoothed using FFT filter over 100 points to reduce the effect of noise on the ring current reading.

We have also included a further discussion further in the manuscript, when discussing faradaic efficiency, which can be found on pages 30 and 31, lines 14 to 19 and 1 to 11. We include below for ease of access for the reviewer:

"The calculated FEs for the catalysts are shown in Table 2. The values range from 0.122 % to 0.020 % at 200 mA cm$^{-2}$, meaning that only a minimal amount of the current at the disc contributed to $ClO^-$ formation, which is then detected at the ring. While the low FE for $ClO^-$ suggests that other processes are consuming the majority of the current, including the OER as the dominant reaction, direct quantification of FE for OER is not achievable because the potential at the ring was held at a value at which exclusively ClRR occurs. The region we highlighted in Fig. 4 shows a distinguishable region for ClRR and a combined reduction region for ORR and $ClO^-$ at potentials less than 0.9 V vs RHE; therefore, identifying a region for pure oxygen will have contributions from $ClO^-$ reduction. A further caveat contributing to the low FEs is the nature of the reaction and the high evolution of gas bubbles,

which can cause deviations in the collection efficiency compared to the previously determined value. This is because bubbles transiently block pathways and the interface between the disc and ring. Several studies highlight this issue encountered when applying inert hydrophobic catalysts to the disc.[10–14] However, it is assumed that this is not the primary issue here, since using our methodology, this issue would have prevented the detection of $ClO^-$ completely, and it is estimated that the catalysts applied here are hydrophilic and demonstrate sufficient surface roughness to aid bubble detachment."

"Furthermore, the RRDE was rotated at 900 rpm, whilst the rotation rate that achieved the highest collection efficiency (500 rpm) is ideal; the rotation rate must be sufficient to ensure bubble disturbance when used in the OER." While a higher rotation rate could help alleviate some bubble noise, it would add further centrifugal and shear forces to the catalyst materials, likely accelerating degradation.

2) The use of a calibration curve derived from homogeneous bulk ClO- solutions to estimate localized, transient ClO- concentrations generated at the disc brings uncertainty.

We thank the reviewer for this comment. The calibration curve was never used as a quantitative metric for assessing hypochlorite; it was more a useful tool to link additional information, namely, the ring currents, to the known concentration of hypochlorite. We stress caution within the manuscript regarding the concentrations with the following paragraph, found on pages 19 and 20:

"One caveat to discuss regarding the calibration curve is that the known concentration of $ClO^-$ is homogeneous throughout the solution and is assumed also to be the initial local concentrations at the RRDE, between the disc and ring. Whereas the $ClO^-$ formed in situ at the disc is not homogeneously distributed in the solution, it is a localised time-dependent concentration at the ring based on the specific conditions at the disc surface, as the initial bulk concentration of $ClO^-$ at the start of the test is equal to zero. Therefore, using the curve to estimate unknown $ClO^-$ concentrations in subsequent electrochemical measurements should be done so cautiously, with this in mind. However, since the comparison between the ring currents of the catalysts is kept consistent with the identical starting electrolyte and parameters applied throughout, it is not affected by this and serves as a useful insight."

We stress throughout the manuscript that the concentration values should be taken lightly as estimates. The research aims to provide a proof of concept, and the main value of this work lies in the observed trends in the ring currents. The calibration curve merely aims to provide another way for the reader to visualise the variations and to give them further context of their meaning in other applications.

3) It remains unclear whether lower ClO- production arises from selectivity effects or from reduced accessibility of COR-active sites. Given the substantial morphological differences among the

catalysts, as shown in the SEM images, ECSA measurements are required to normalize the data to electrochemically active surface area or intrinsic activity.

Thank you for this suggestion; it is a critical point. We have attempted to enhance the clarity of the morphological differences. We investigated ECSA measurements in the previously mentioned work we conducted on these catalyst materials. We have included the data below:

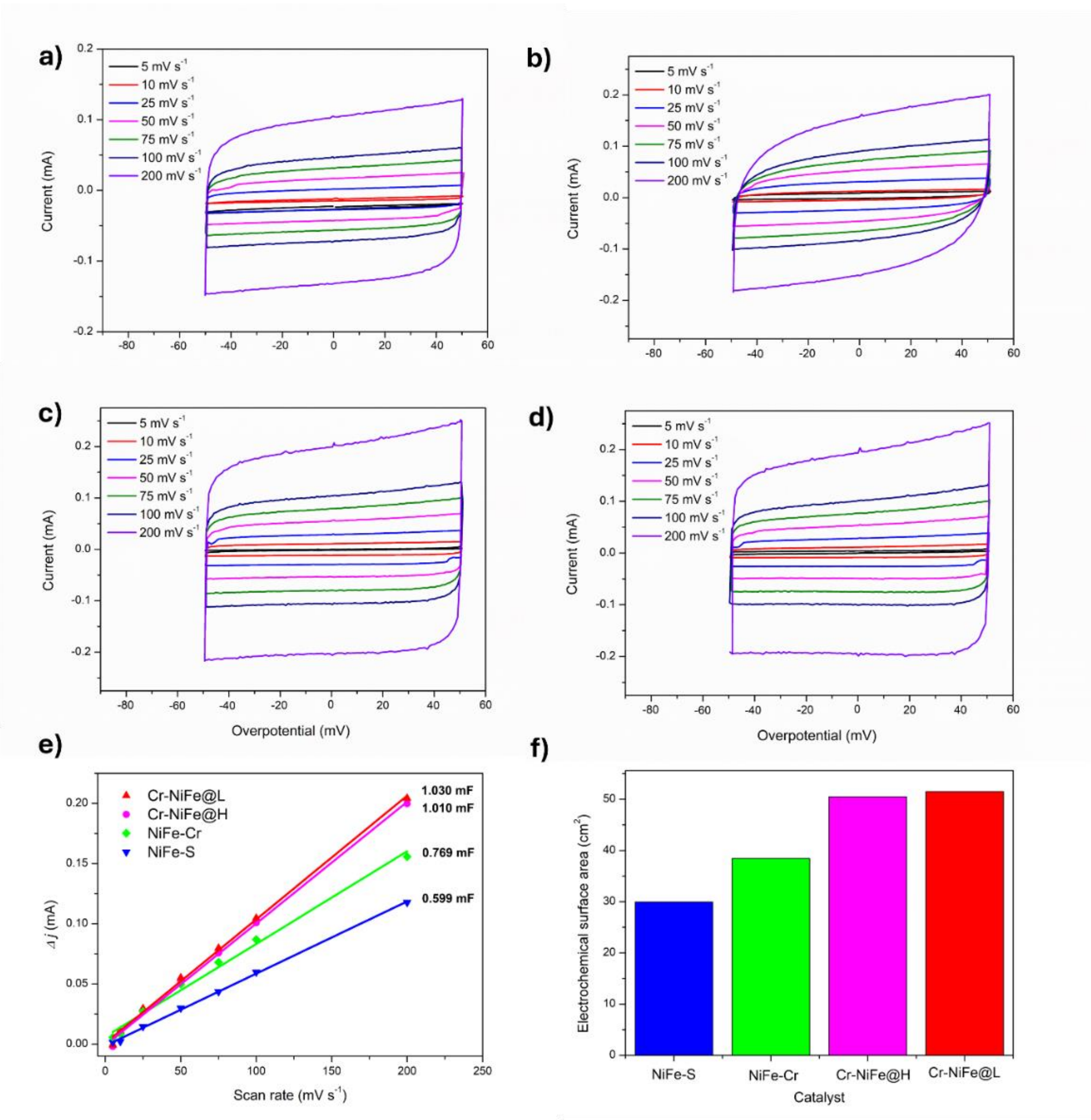


Figure 3 CVs of varied synthesis current densities on 1 $cm^2$ of Ni mesh. $C_{dls}$ of a) NiFe-S b) NiFe-Cr c) Cr-NiFe@L d) Cr-NiFe@H. Average current ($Dj=(j_a-j_c)/2$) against the scan rate showing the double-layer capacitance ($C_{dl}$) extracted from the corresponding CVs of various corrosion-resistant catalysts f) ECSAs of varied corrosion-resistant catalysts.

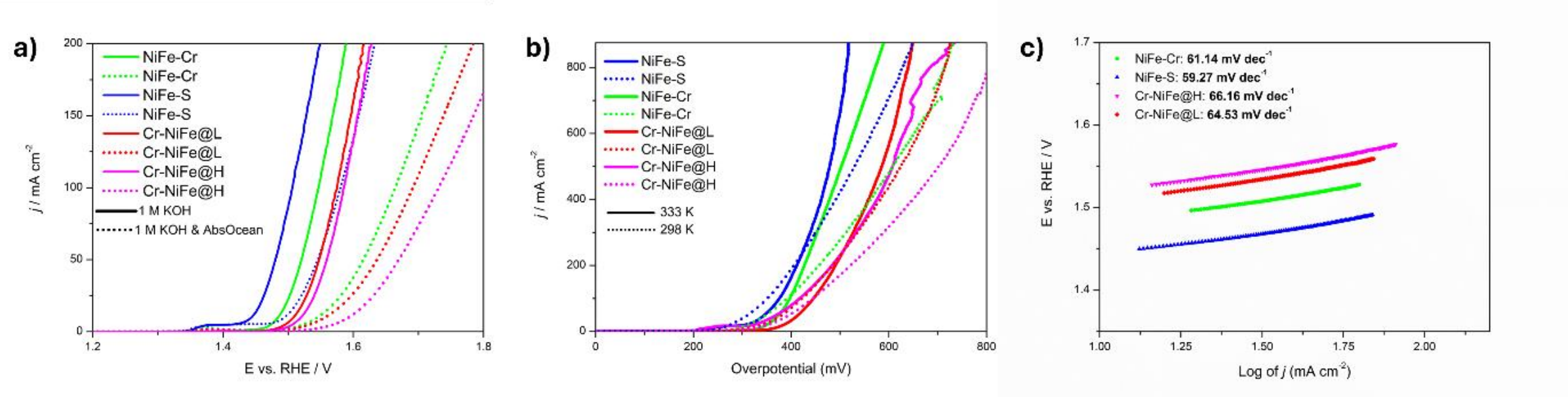


Figure 4 a) LSV curves of catalyst-coated mesh in 1 M KOH & 1 M KOH + AbsOcean at 298K at a scan rate of 1 $mVs^{-1}$ with an 80% iR compensation b) LSV curves of catalyst-coated foam in 1 M KOH & AbsOcean at 298K vs 333K at a scan rate of 1 $mVs^{-1}$ with an 80% iR compensation c) Tafel analysis of catalyst-coated mesh in 1 M KOH at 298K at a scan rate of 10 $mVs^{-1}$.

The electrochemically active surface area (ECSA) was determined through electrochemical double-layer capacitance ($C_{dl}$) measurements from CV scans at different scan rates (5 to 200 mV $s^{-1}$) (*Figure 3*) in the non-Faradaic region, which is within a ± 50 mV window of the open circuit potential (OCP).[15] Cr-NiFe@L exhibits the highest $C_{dl}$ (1.03 mF), marginally higher than Cr-NiFe@H (1.01 mF), with NiFe-S highlighting the lowest $C_{dl}$ at 0.599 mF, which based on the morphology is expected, based on the increased roughness of the secondary Cr layer compared to the NiFe-S catalyst. Figure *3*f shows the ECSAs for the catalyst materials, calculated using a specific capacitance for Ni (20 µF $cm^{-2}$). Ni is used as it is the dominant element across the catalyst materials.[16,17]

Interestingly, based on electrochemical tests in *Figure 4*, the ECSA of the catalyst materials has no correlation with increased electrochemical performance, indicating that catalytic performance is primarily dictated by the intrinsic activity of the catalyst materials, rather than the topography. Therefore, applying ECSA normalisation to the materials would likely result in the same electrochemical performance trend, and the differences between the catalysts would be more pronounced.

**Reviewer #2 (Remarks to the Author):**

The author used the rotating ring disk electrode (RRDE) device to compare two strategies for inhibiting COR and CER, independently detecting the formation of hypochlorite at the disk in 1 M KOH and 0.5 M NaCl (simulated seawater) solutions. The results indicated that in alkaline seawater, the ion-selective strategy was more effective in reducing the formation of hypochlorite. However, the electrostatic repulsion strategy could improve selectivity to reduce the formation of hypochlorite in terms of electrochemical performance, but its performance slightly declined. This insight will greatly enhance the efficiency and development level of oxygen evolution reaction electrocatalysts in seawater electrolysis, as it can simplify the design of oxygen evolution reaction catalysts. However, the manuscript still has many issues that require attention. It is recommended for major revision.

We thank the reviewer for the positive feedback and trust that the revised manuscript addresses the concerns raised.

1.The title is misleading and may give the impression that the authors have developed a ring-disc electrode for evaluating hypochlorite selectivity. The authors need to rewrite the title carefully.

We thank the reviewer for highlighting this crucial point, and we agree that the title could be construed to be the study developing a new RRDE; thus, we have slightly altered the title to reflect that the study uses a RRDE rather than develops one, we have included the altered title below:

**Previous title:** Rotating ring-disc electrode for assessing hypochlorite selectivity of corrosion resistance OER electrocatalysts for alkaline seawater splitting.

**New title**: Assessing hypochlorite selectivity of corrosion resistance OER electrocatalysts for alkaline seawater splitting using a rotating ring-disc electrode.

2.The abstract section is too long. The authors need to condense and optimize it.

Following the suggestion, we have made a good effort to condense the abstract into a more streamlined version. We have reduced the abstract from 307 to 195 words.

3.In the Graphical abstract, there is too much text and it is too small, making it difficult to read.

We thank the reviewer for highlighting this point. We agree that we had integrated too much into the graphical abstract, so we reduced the number of images and increased the figure size to improve readability. We have included the figure below for ease of access:

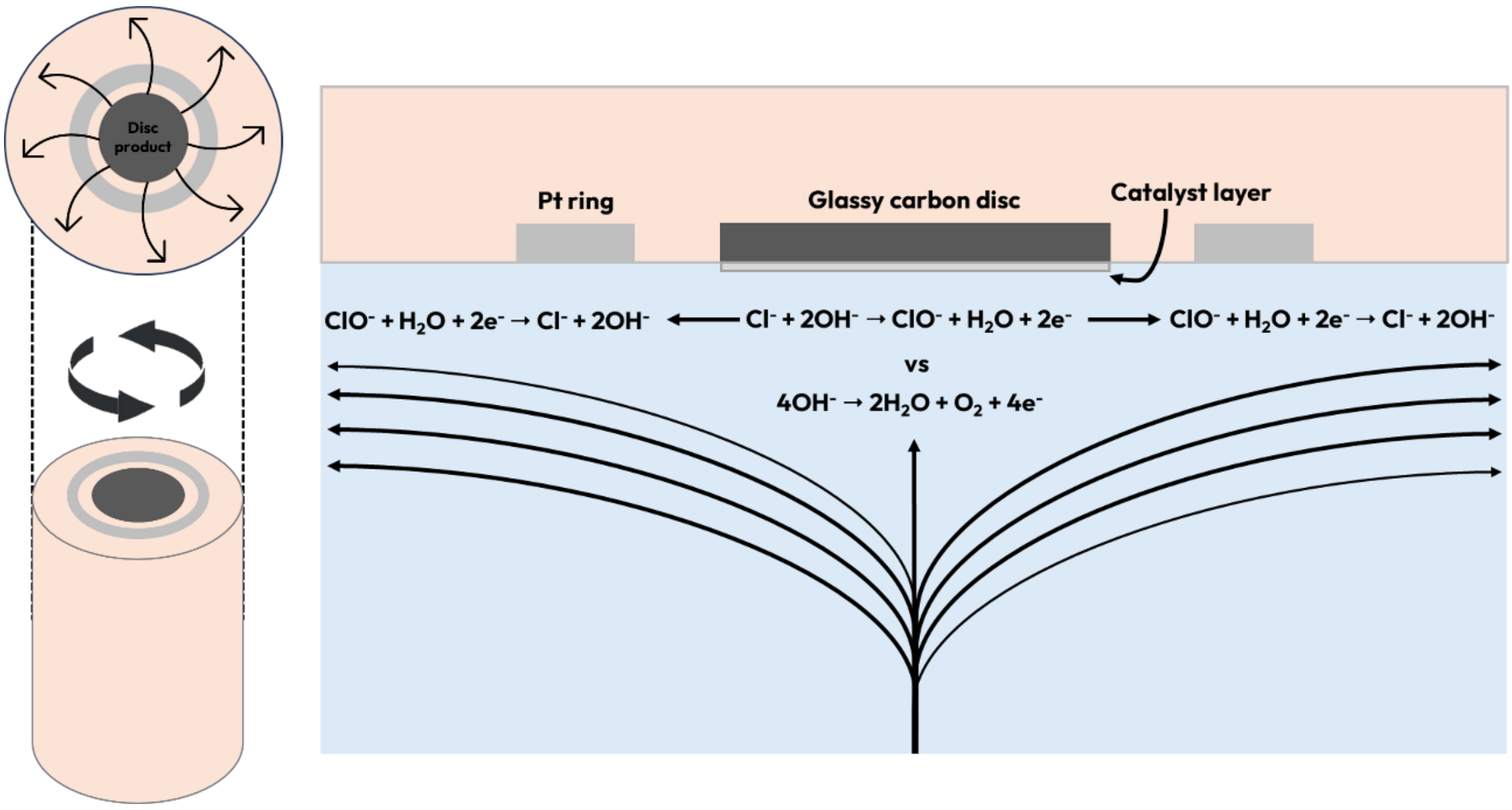


**Table of contents entry**: Experiment methodology using an RRDE to detect hypochlorite reduction in alkaline simulated seawater electrolytes.

4.The author should pay attention to the reliability of the evidence. Some of the content goes against basic common sense. For instance, the sentence "However, creating sustainable hydrogen remains expensive, as most production methods currently rely on fossil fuels." is not very reasonable. It is well-known that the current price of green hydrogen is much higher than that of grey hydrogen and blue hydrogen. There are many such questionable statements like this, and the author needs to pay attention to them. At the same time, the author's writing in the introduction section is overly obscure and full of leaps in logic, making it extremely difficult to understand. The author will discuss the significance of hydrogen for a while, then write about water scarcity, and also about the Australian deserts, and so on. It is suggested to focus on one main point and develop it accordingly.

Thank you for highlighting this. We have carefully revised the introduction to reduce ambiguity in some sentences and to develop points, particularly early on. These changes can be found on page 2, lines 10 to 16 and page 3, lines 1 to 6.

5. CER and COR exhibit better inhibitory effects. However, it is worth noting that from the physical property characterization results of the manuscript, the surfaces of these catalysts are not the result of normalization. As shown in the scanning electron microscope test results in Figure 2, the particles after Cr doping have significantly increased, which will significantly increase their active sites. Then, to what extent is the author's demonstrated advantage of Cr derived from the increase in specific surface area? The author needs to normalize the relevant data using the specific surface area method and the electrochemical surface area method.

We thank the reviewer for this suggestion, and we note that it has been highlighted by another reviewer also; it is a critical point. We include the same response as we did for the other reviewer which can be found below:

We have attempted to enhance the clarity of the morphological differences. We investigated the ECSA measurements in the previously mentioned work on these catalyst materials and have included the data below:

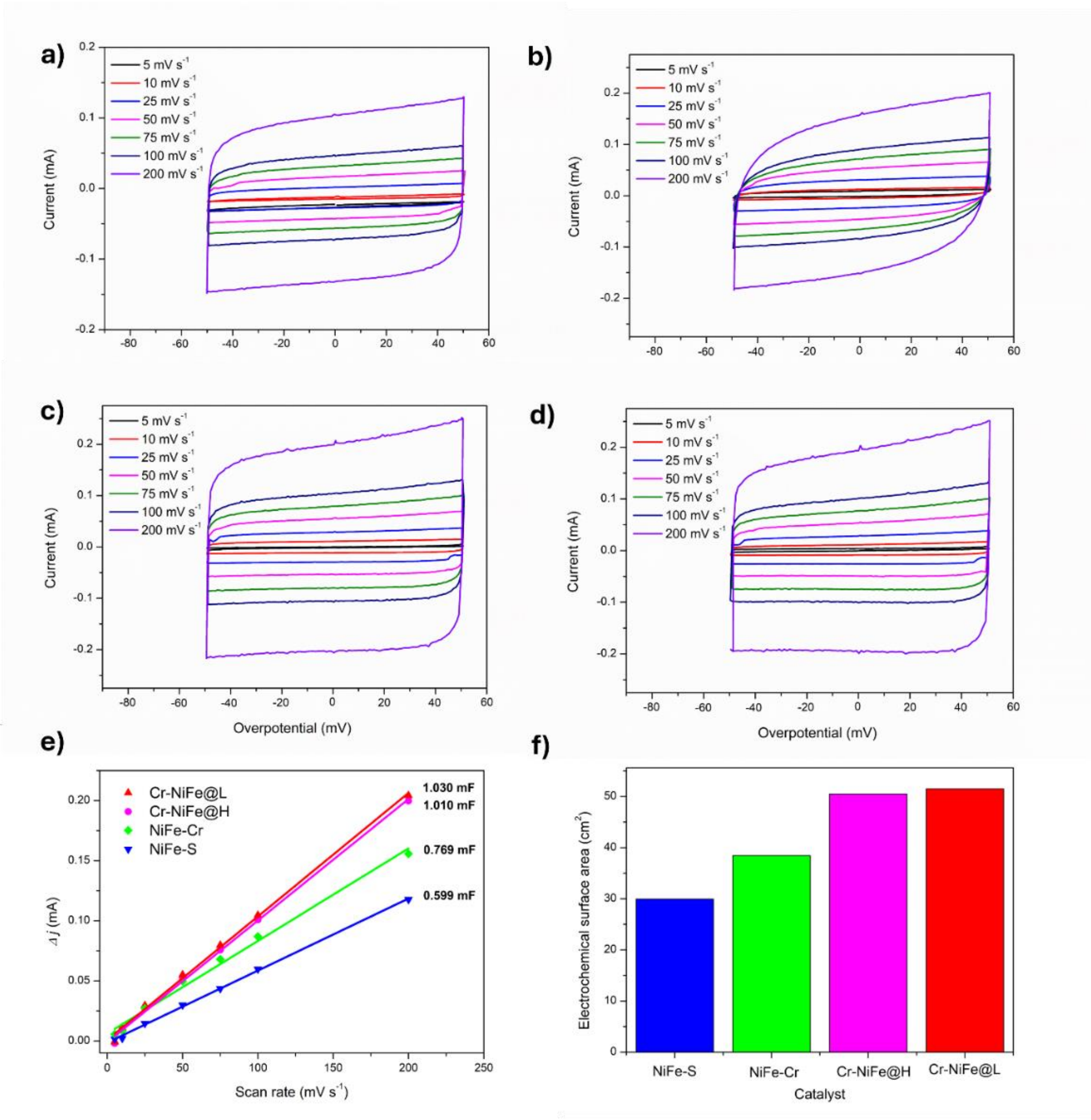


Figure 5 CVs of varied synthesis current densities on 1 $cm^2$ of Ni mesh. $C_{dls}$ of a) NiFe-S b) NiFe-Cr c) Cr-NiFe@L d) Cr-NiFe@H. Average current ($Dj=(j_a-j_c)/2$) against the scan rate showing the double-layer capacitance ($C_{dl}$) extracted from the corresponding CVs of various corrosion-resistant catalysts f) ECSAs of varied corrosion-resistant catalysts.

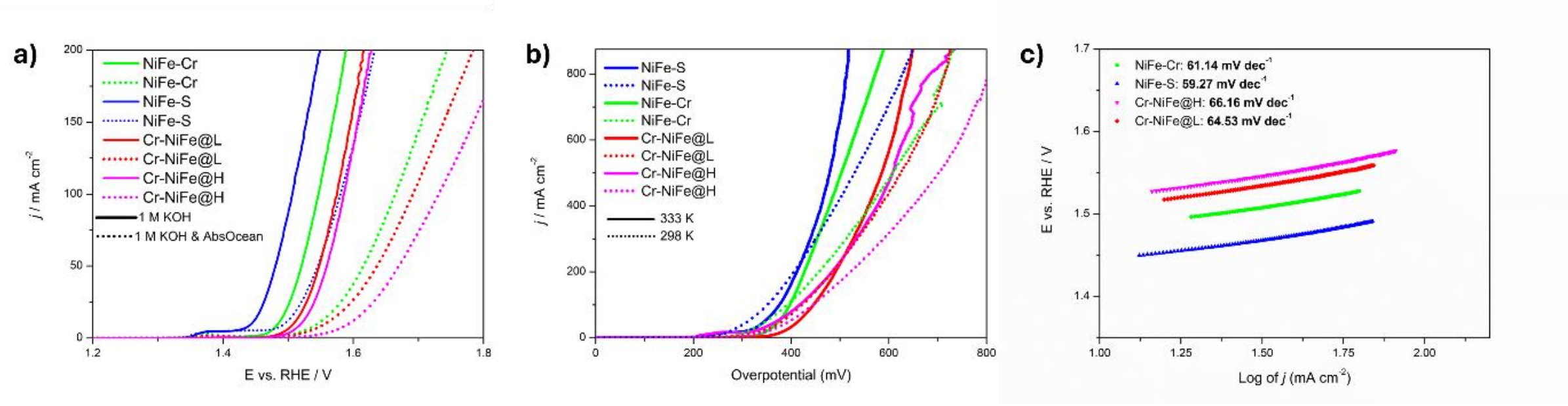


Figure 6 a) LSV curves of catalyst-coated mesh in 1 M KOH & 1 M KOH + AbsOcean at 298K at a scan rate of 1 $mVs^{-1}$ with an 80% iR compensation b) LSV curves of catalyst-coated foam in 1 M KOH & AbsOcean at 298K vs 333K at a scan rate of 1 $mVs^{-1}$ with an 80% iR compensation c) Tafel analysis of catalyst-coated mesh in 1 M KOH at 298K at a scan rate of 10 $mVs^{-1}$.

The electrochemically active surface area (ECSA) was determined through electrochemical double-layer capacitance ($C_{dl}$) measurements from CV scans at different scan rates (5 to 200 mV $s^{-1}$) (Figure 3) in the non-Faradaic region, which is within a ± 50 mV window of the open circuit potential (OCP).[15] Cr-NiFe@L exhibits the highest $C_{dl}$ (1.03 mF), marginally higher than Cr-NiFe@H (1.01 mF), with NiFe-S highlighting the lowest $C_{dl}$ at 0.599 mF, which based on the morphology is expected, based on the increased roughness of the secondary Cr layer compared to the NiFe-S catalyst. Figure 5f shows the ECSAs for the catalyst materials, calculated using a specific capacitance for Ni (20 µF $cm^{-2}$). Ni is used as it is the dominant element across the catalyst materials.[16,17]

Interestingly, based on electrochemical tests in Figure 4, the ECSA of the catalyst materials has no correlation with increased electrochemical performance, indicating that catalytic performance is primarily dictated by the intrinsic activity of the catalyst materials, rather than the topography. Therefore, applying ECSA normalisation to the materials would likely result in the same electrochemical performance trend, and the differences between the catalysts would be more pronounced.

6. All the statistical data in the manuscript are derived from a single one-time test result. Due to the significant systematic error in electrochemical testing, the author needs to increase the number of tests for each data point and include the error range in all the statistical graphs. This thereby enhances the reliability of the data.

We thank the reviewer for highlighting such a critical point. In the supporting information, we have created a new figure on page 8 (Fig. S9) and provided an analysis based on this on page 8, lines 8 to 11 and page 9, lines 1 to 18. We have included the figure below for ease of access. Specifically, we have created a statistical display using box plots to represent any variations in the average ring current densities, particularly at high disc current densities. Each test of the catalyst material was conducted at

least three times to ensure reproducibility, but often more to confirm the methodology. The box plots take into account the variations observed (Fig. S9 a-b), and due to the high number of tests, some variation was found; it is with confidence that we have been able to exclude some values as anomalies, specifically with NiFeS at 100 mA $cm^{-2}$ (Fig. S9 c-d). Our hypothesis regarding the variation in NiFeS is that the materials developed here were based on an existing recipe, and the corrosion-resistant modifications (addition of thiourea or chromium) were added in a simplistic manner and not optimised. While the stability of these materials in a static electrolyte was sufficient, their application to a rotating electrochemical setup, such as an RRDE or RDE, introduces centrifugal forces and viscous drag, both of which may contribute to material degradation during testing and lead to deviations in the ring current response, which can be attributed to greater hypochlorite formation. However, a key point to highlight with these box plots is that the trend in selectivity of the catalysts does not change, and we can say with confidence that the overlayer of chromium has a direct effect on reducing the ring current, which can be linked to hypochlorite production at the disc electrode.

We have also updated the average ring currents in Fig. 6 and Fig. 7 to better reflect the values obtained across the reproducibility graphs in Fig. S9. The updated values have thus led us to update Fig. 8 and Tables 1 and 2 in the manuscript. We are confident that the modified ring currents in the manuscript reflect the averages across the tests, and we have updated the values to clarify the averages.

Ultimately, it is acknowledged that this RRDE methodology/protocol is not without limitations; the conclusions drawn in this study can only be explicitly concluded within the materials used.

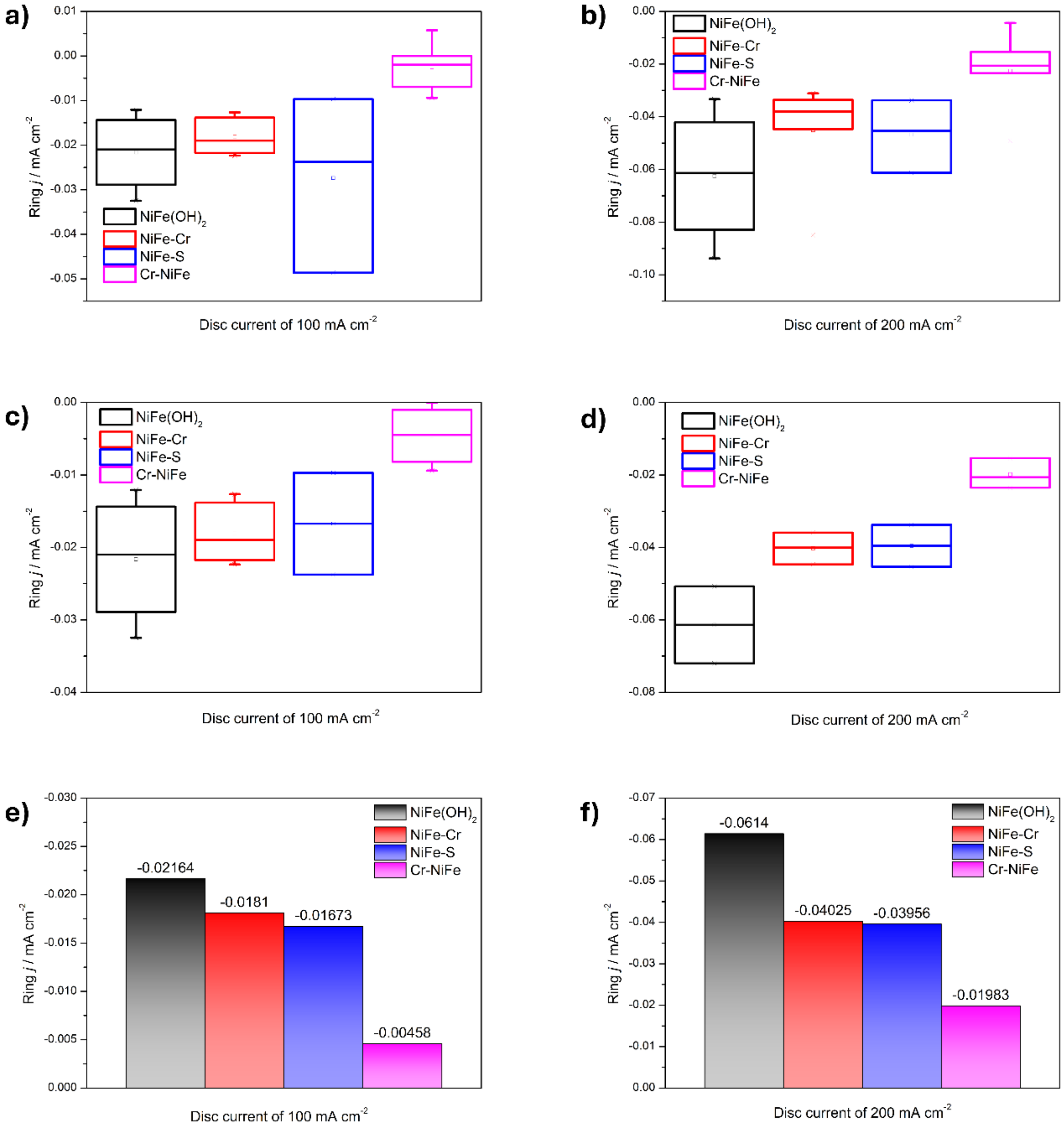


Fig. S9 Statistical reproducibility displayed using box plots of the average ring currents taken over a course of multiple tests. Each experiment was subject to at least 3 repeats a) all ring currents recorded during electrochemical testing at a disc current of 100 mA cm$^{-2}$ b) all ring currents recorded during electrochemical testing at a disc current of 200 mA cm$^{-2}$. Ring currents recorded during electrochemical testing with anomalous values removed and taking the 3 most consistent data points c) at a disc current of 100 mA cm$^{-2}$ d) at a disc current of 200 mA cm$^{-2}$.

7. Due to the significant differences in the surface of the samples, in order to ensure the accuracy and rationality of the final conclusion, it is suggested that the author add higher magnification scanning electron microscope images or transmission electron microscope images to present more detailed information.

We thank the reviewer for highlighting this. We have included the following TEM imagery from a separate manuscript on these identical materials. Figure 7 and Figure 8 both show TEM analysis on the materials used in this study, down to a 5-nanometer magnification. We hope that these figures provide clarity for the reviewer, but we are unable to include these figures as they are under review with a separate journal in a different manuscript.

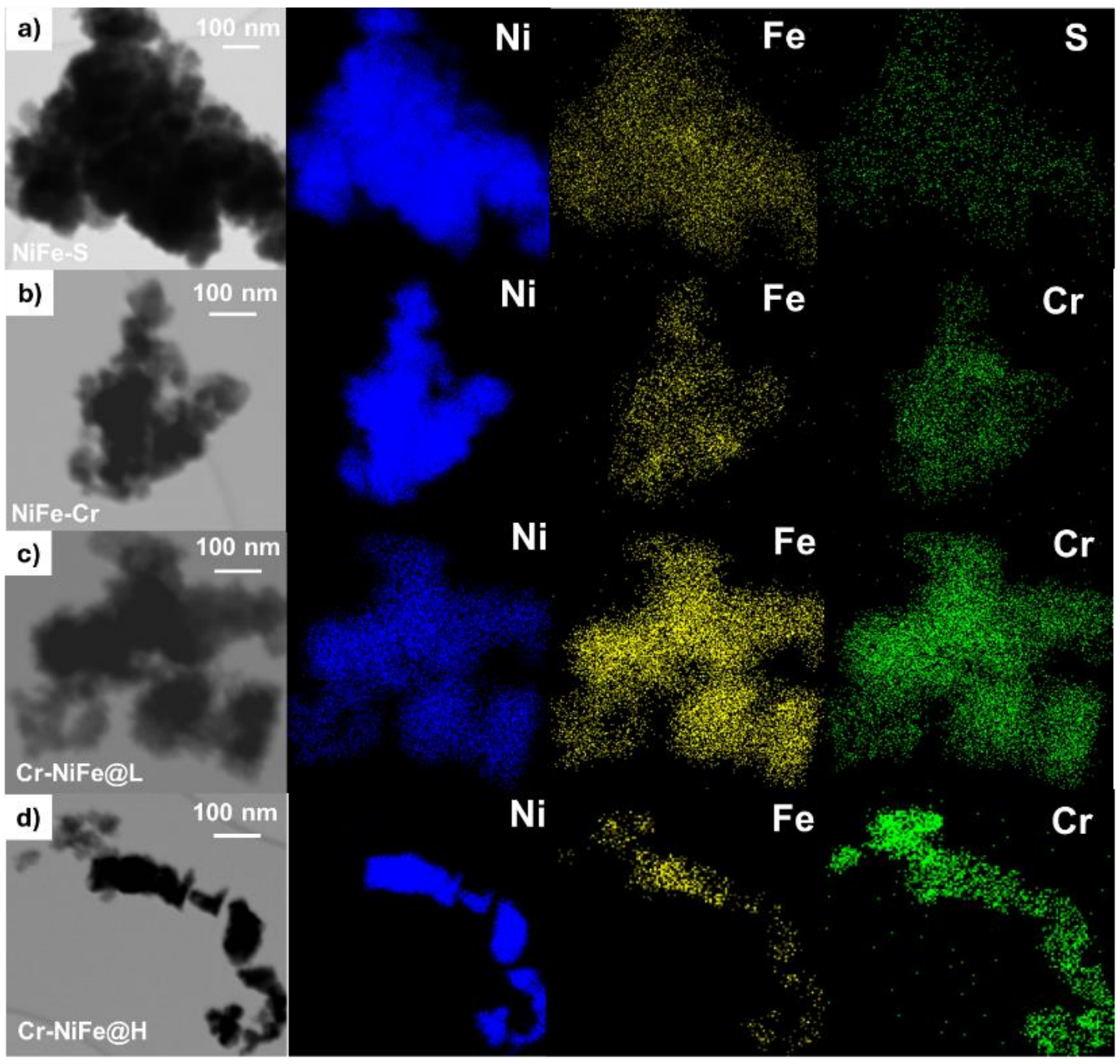


Figure 7 Transmission electron microscopy energy dispersive spectroscopy (TEM-EDS) mapping of corrosion-resistant catalysts on carbon plate deposited from 18 mM metal sulphate & 25 mM ammonium sulphate using -50 mA $cm^{-2}$ for 210s using a magnetic stirrer at 500 rpm a) NiFe-S b) NiFe-Cr c) Cr-NiFe@L d) Cr-NiFe@H.

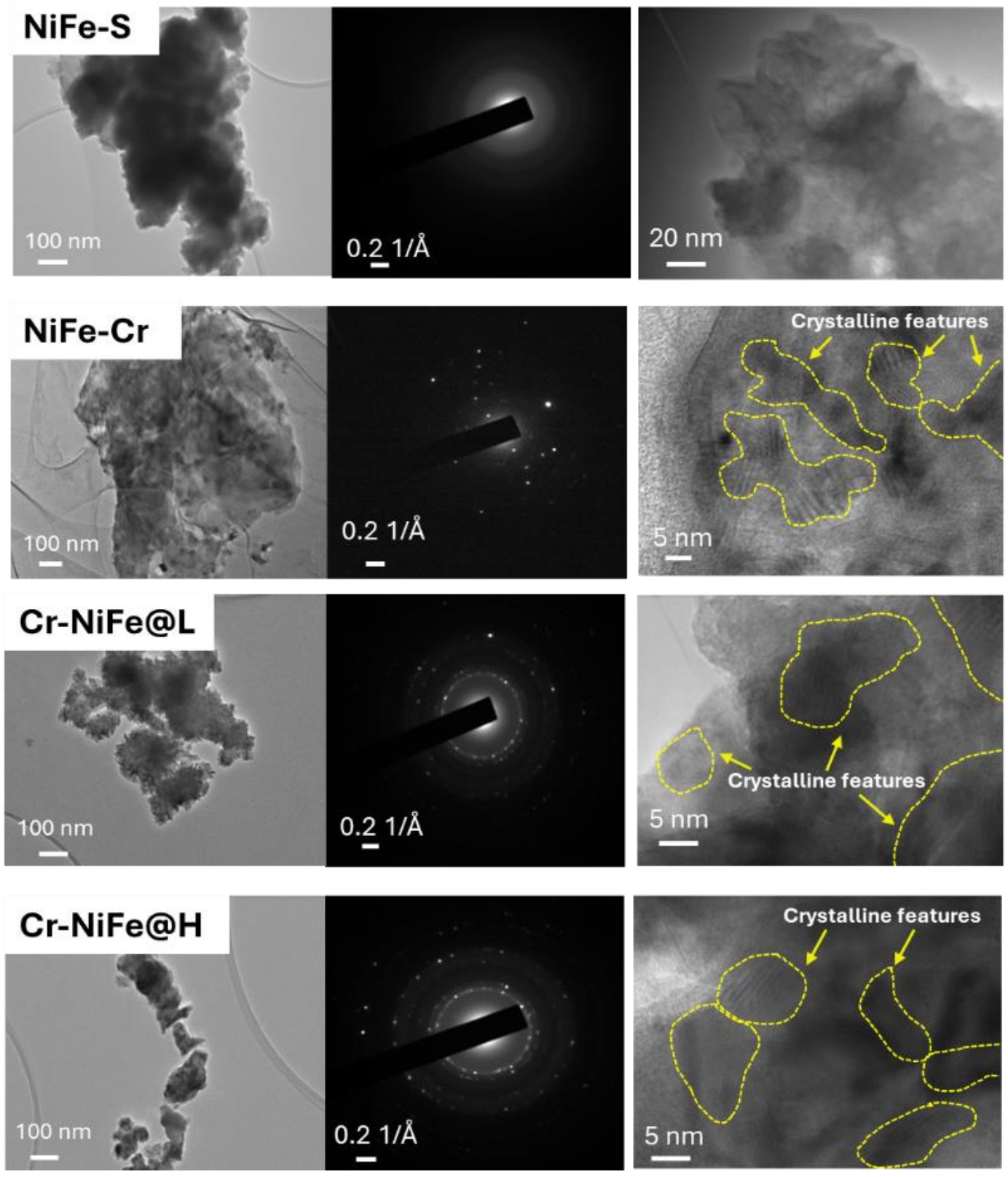


Figure 8 Low-magnification TEM image, SAED ring diffraction pattern and high-magnification TEM images showing crystalline features of corrosion-resistant catalysts.

**Reviewer #3 (Remarks to the Author):**

Major revision is required before the work is considered for publication.

1. The introduction is overly long and reads like a literature review rather than a focused motivation for the present study.

We thank the reviewer for this comment. We appreciate what the reviewer is saying, and we have removed some sections of the introduction that mention various catalyst materials of the different corrosion-resistant techniques and have streamlined the overall introduction to be more focused. We hope that the revised version satisfies the reviewers' comments. The introduction has been reduced from 1282 words to 1046 words.

2. In experimental section, the authors report the synthesis of four catalysts (NiFe(OH)2, NiFeS, NiFeCr, and CrNiFe) using electrodeposition. Forming phase-pure materials via electrodeposition typically requires careful optimization, but no systematic optimization process is described. This raises concerns regarding the reproducibility and phase purity of the synthesized catalysts.

The reviewer raises a really good point here. The motivation of this study was to better understand the modification strategies made to the NiFe electrocatalyst, namely the addition of sulphur and chromium, which can be seen to optimise certain parameters (selectivity) but detrimentally affect others (catalytic activity and, to some extent, stability). Thus, we acknowledge modifications to the precursor concentrations and the way in which these modifications are applied to the NiFe electrocatalyst could be further optimised, either through different synthesis methods (e.g., hydrothermal) or by varying concentrations to identify an optimal relationship between catalytic activity, stability and selectivity. Further optimisation could involve adding substances to the precursor solution to improve adhesion between the substrate and catalyst layer; however, this was not within the scope of this study, as it would have introduced time constraints and distracted from the primary objectives of evaluating the selectivity between catalysts as clearly as possible.

We fully agree that such optimisation would provide additional insight and could further strengthen the work. However, due to the significant cost and time required to conduct this additional study, we believe it is beyond the scope of the current manuscript.

3. On page 14 (lines 17–22), the authors should clarify whether the synthesized material is NiFeSx or sulphur-doped NiFe(OH)2. Identification of the material is provided. Additional structural characterization, such as XRD, is recommended to confirm the phase and composition of the synthesized material.

We thank the reviewer for highlighting this crucial point. We believe that the NiFeS is more of a sulphur-doped NiFe(OH)2, with some features of nickel iron sulphide, since we believe there are some mixed Ni-Fe sulphide phases. We come to this conclusion since only small amounts of sulphur are included as a modification strategy, for a true NiFe sulphide, the sulphur would form Ni-S and Fe-S bonds, over hydroxide groups, which is the opposite of what we identified using Raman spectroscopy (Figure 9). Furthermore, NiFe sulphide materials would typically exhibit a crystal lattice built around $S^{2-}$ ions, of which we don't see in the XRD patterns (Figure 11), and from the TEM imagery (Figure 11), the NiFeS is completely amorphous.

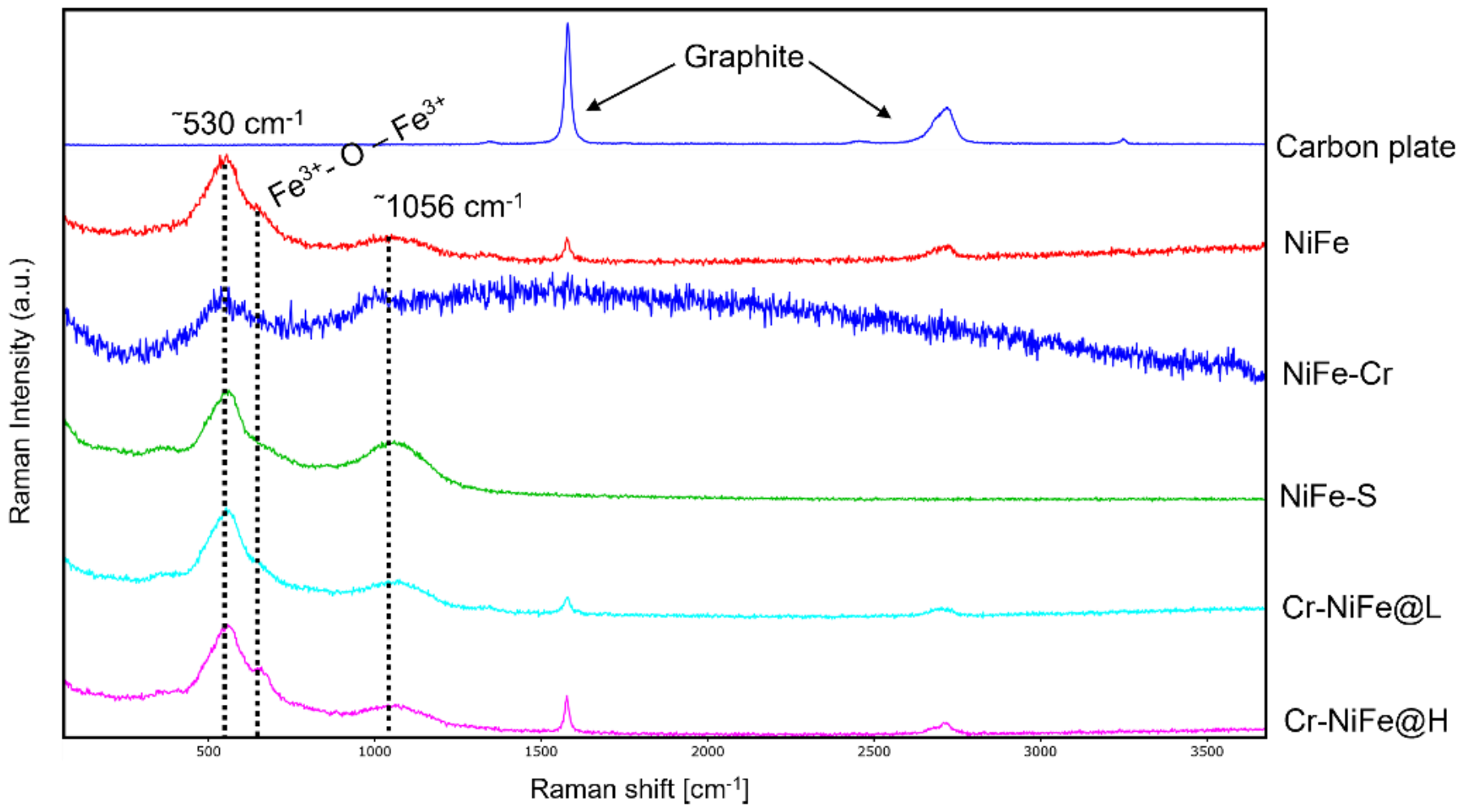


Figure 9 Raman spectroscopy of modified catalyst materials on a carbon plate, surface area 1 $cm^2$.

Based on the analysis conducted in a separate study on these exact materials, we include the Raman spectroscopy analysis (below) to shed further information about the materials for the reviewer:

"Ex-situ Raman spectroscopy was employed to gather more information on the modified catalysts and to determine whether the modification techniques alter the characteristics of the NiFe catalyst. Figure *9* shows the spectra for each of the modified samples and is subsequently overlaid. Despite the modifications, all catalysts share very similar bonding characteristics, and the breadth of the peaks is characteristic of amorphous electrodeposited materials.[18] The dominant peak at ˜530 $cm^{-1}$ is a distinctive feature of disordered/ defective β-$Ni(OH)_2$, commonly reported by other authors.[1,18–21] However, this region can consist of α & β-$Ni(OH)_2$, but the broadness of the peaks complicates deconvolution; typically, elements of α-$Ni(OH)_2$ are observed at ˜460 $cm^{-1}$, which contribute to the wider bases of the peaks; α-$Ni(OH)_2$ is another form of metal-oxygen vibration. The width of the peak can be affected by higher Fe contents, hence the shoulder peak observed; this is hypothesised to be influenced by the $Fe^{3+}$-O-$Fe^{3+}$ structure.[18,19,22,23] The expanded Raman shift region shows that only a graphite peak is observed near 1500 $cm^{-1}$ and 2750 $cm^{-1}$, stemming from the carbon plate used as a substrate. The peak observed at 1056 $cm^{-1}$ can also be linked to C-C bond stretching vibration stemming from the substrate.[24]"

4. TEM analysis is recommended to explain the effect of chromium modification on NiFe(OH)2. Although XPS suggests the oxidation states in the NiFeCr and CrNiFe samples, complementary TEM and XRD analyses can provide more insight into chromium distribution, phase formation, and its structural position in NiFe(OH)2.

We thank the reviewer for highlighting this very important point. The reviewer is right: XRD and TEM are very useful techniques for providing more information about the catalyst materials. These techniques were not included in the initial manuscript because we had a separate paper focused on the in-depth characterisation of the materials and their intrinsic properties. This manuscript aims to complement the previous manuscript by focusing on the variations in selectivity introduced by the experimental methodology. Furthermore, XRD did not reveal any additional information about the catalysts due to the materials' amorphous nature. TEM did provide some highlights of the variations in particle distribution; thus, we have included them below for the reviewer's clarity. However, as this data is included with another journal, it is not included in the manuscript here.

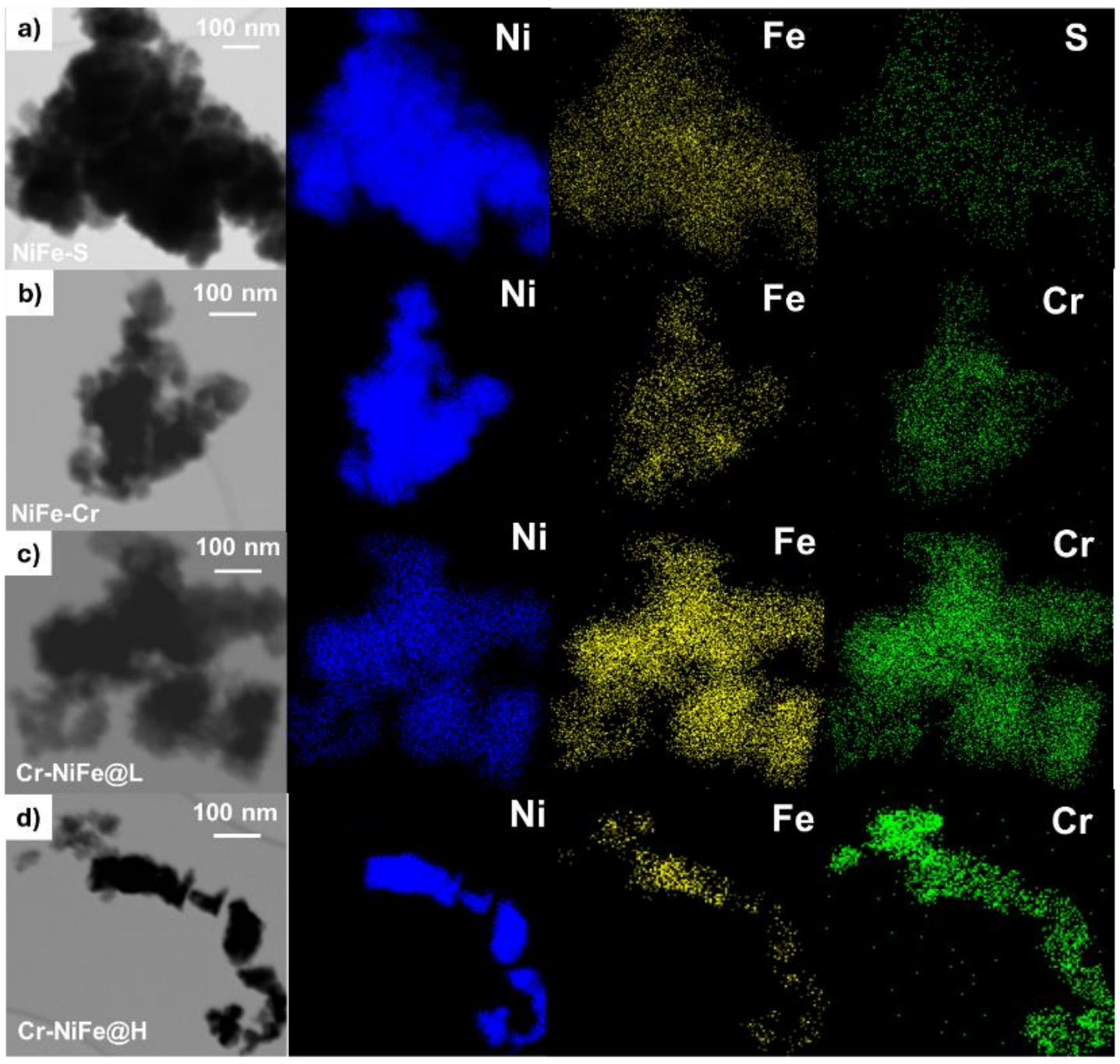


Figure 10 Transmission electron microscopy energy dispersive spectroscopy (TEM-EDS) mapping of corrosion-resistant catalysts on carbon plate deposited from 18 mM metal sulphate & 25 mM ammonium sulphate using -50 mA cm$^{-2}$ for 210s using a magnetic stirrer at 500 rpm a) NiFe-S b) NiFe-Cr c) Cr-NiFe@L d) Cr-NiFe@H.

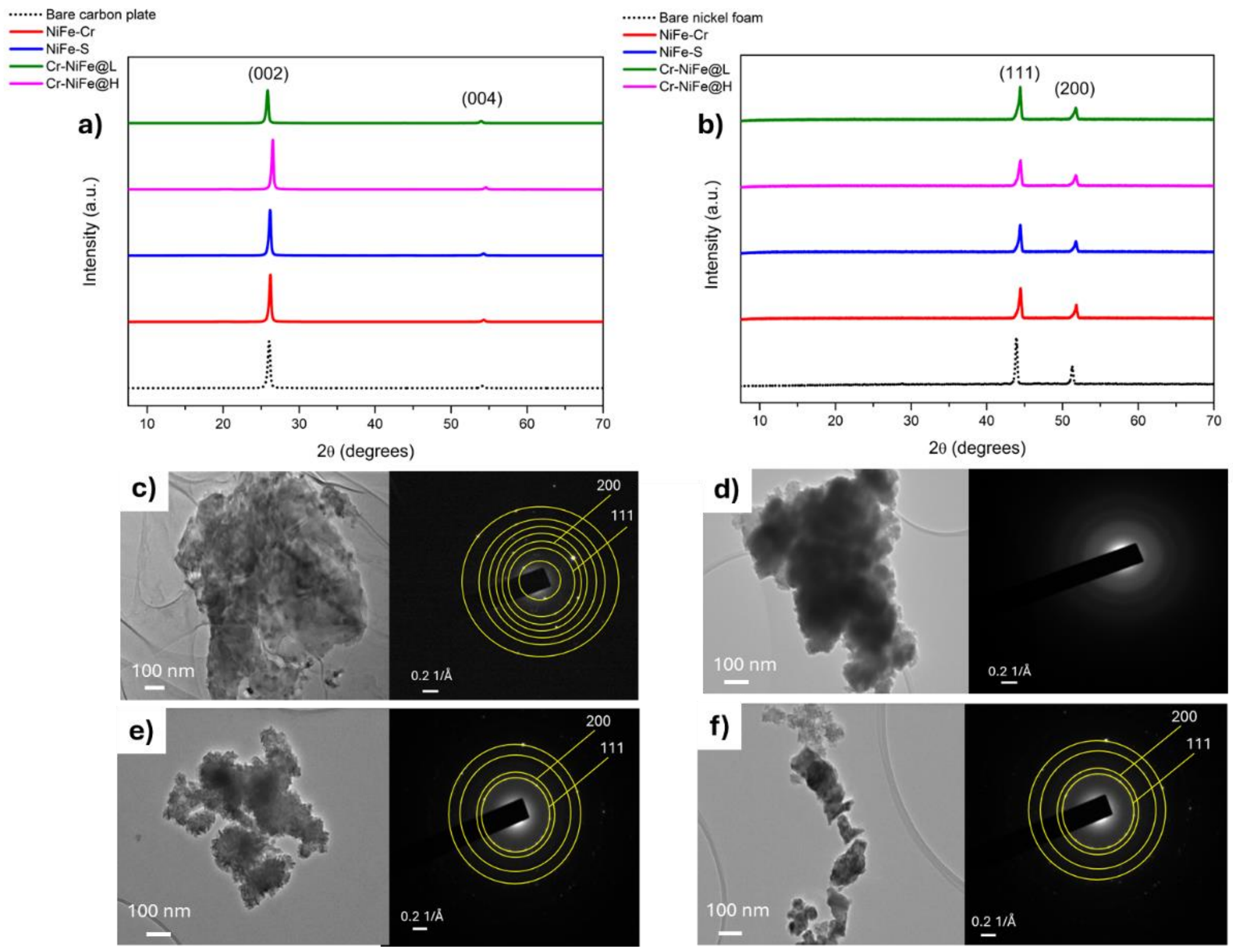


Figure 11 XRD patterns of deposited corrosion-resistant coatings on a) 1 $cm^2$ of carbon plate using -50 mA $cm^{-2}$ for 210s b) 1 $cm^2$ of nickel foam using -50 mA $cm^{-2}$ for 210s. Low-magnification TEM image and SAED ring diffraction pattern of c) NiFe-Cr d) NiFe-S e) Cr-NiFe@L f) Cr-NiFe@H.

Figure *11* a & b show no peaks associated with the catalyst coatings, only peaks from the substrate. Distinct diffraction peaks for raw graphite; an intense crystalline peak is observed at a 2θ of 26.4˚, with a lattice spacing of ˜3.4 Å, which links to the diffraction plane of graphite (002). A smaller peak is observed at a 2θ of 54.5°, corresponding to the (004) diffraction plane.[1] Initially it was anticipated that the carbon substrate (Figure *11*a), due to its high crystallinity, drowned out any catalyst features, yet no catalyst features were observed, still, this was later confirmed by using a similarly crystalline NF substrate (Figure 2b), leading us to the conclusion that all the deposits are amorphous. The high-intensity peaks of the coated NF substrate pattern at 44.5° and 52° correspond to metallic Ni (COD 2100646), originating from the metal NF substrate (Figure 2b), with a calculated lattice spacing of approx. 2 Å and 1.7 Å, respectively, which relate to planes of (111) and (200).[2] Furthermore, the peak intensity of NF decreases upon catalyst application, further confirming the catalyst's amorphous nature. Since a significant portion of the crystallinity arises from the metallic substrate, we conclude that the catalyst is amorphous primarily, as also confirmed in our previous work.[1] Amorphous materials lack defined crystalline structures and, accordingly, they provide structural flexibility, increased ECSA, and improved stability. [3–5]

TEM analysis was performed to further investigate the crystallinity of the deposit and identify any short-range nanometer-scale crystalline features for the catalyst materials (Figure *11* c-f). The analysis shows TEM micrographs of the various catalysts, along with selected area electron diffraction (SAED) patterns. From the SAEDs, it is evident that some dotted ring patterns were observed, indicating minor crystalline areas across all Cr containing samples, whilst NiFe-S displayed solid rings, which suggests it remains completely amorphous. From the dotted ring patterns in the SAEDs, interplanar d-spacing can be determined from the SAEDs using Bragg's law and is related to the Bragg angle. The crystalline regions with a lower Bragg angle (closer to the centre) in the SAEDs correspond to metallic species, with spacings of approximately 2.0 Å and 1.7 Å, related to the standard diffraction planes (111) and (200) for Ni metal. [1,6–8] It is generally understood that the Cr doped catalyst catalysts are predominantly amorphous, with some crystalline features linked to the mixed metal deposition that results from the reduction of Ni and Fe during cathodic electrodeposition. This is supported by the fact that samples were prepared on a carbon plate and the deposited materials were scraped off, emphasising the Ni observed here is from the catalyst materials, not the substrate. Notably, NiFe-S has a completely amorphous structure, which is hypothesised to be due to two possible reasons. Firstly, the lower overall metal ratio in the electrodeposition solution provides less divalent Ni and Fe to be reduced. Secondly, thiourea acts as a complexing agent, binding with metal ions. In this context, $Ni^{2+}$ ions can coordinate with thiourea, thereby forming stable surface complexes that inhibit further reduction of these ions.[9]

5. Figures 5e and 6a show different current values despite apparently using the same catalysts and experimental conditions. The authors should clarify the reason for this discrepancy.

We thank the reviewer for highlighting this point, and we apologise for the lack of clarity. The same values are used, but a different scale is applied across the graphs, resulting in a visual disparity. We have improved the scale on figure 5 to improve the readability, as well as increasing the font size.

6. The authors should explain the origin of the performance difference between NiFeCr and CrNiFe catalysts, as both contain chromium species. The limited characterization makes it difficult to rationalize their different electrochemical and corrosion-resistance behaviors. In addition to chromium content, critical factors such as chromium distribution, oxidation state, phase composition, crystallinity, defect density, surface composition, and microstructural differences may influence corrosion resistance and should be considered.

The reviewer highlights a very good point here. The modification of applying an ion-selective material within the secondary deposition is more detrimental to OER catalytic activity than applying it in a singular electrodeposit. It is hypothesised that the more catalytically active sites are covered with Cr ($Cr(OH)_3$ and $Cr_2O_3$). The impact of Cr modification varies with composition; some systems exhibit a non-linear correlation between Cr content and catalytic activity, suggesting that too much or

poorly integrated Cr can be detrimental to performance.[25] We have included figure 12 from a separate manuscript to highlight the impact of the secondary Cr depositions yielded higher Cr contents (Cr-NiFe@L (not used in this study) and Cr-NiFe@H which is used in this study), and of the two secondary Cr depositions, the one performed at higher current density deposition (Cr-NiFe@H) displayed a marginally worsened performance compared to the lower current density deposition (Cr-NiFe@L) for the same reason (higher Cr contents), as shown in Figure 12 a-b. It is estimated that, while the hard Lewis acid coating was intended to improve the corrosion resistance of the NiFe catalyst layers, the instability originates from poor adhesion of the Cr deposited onto the NiFe. While the presence of mixed $Cr_2O_3$ and $Cr(OH)_3$ phases offers corrosion resistance by forming locally passivating domains, it is hypothesised and supported in literature that the oxide/hydroxide-rich regions undergo progressive oxidation and dissolution under high anodic OER conditions, leading to partial surface reconstruction and exposing the underlying NiFe phase to chloride attack.[26,27]

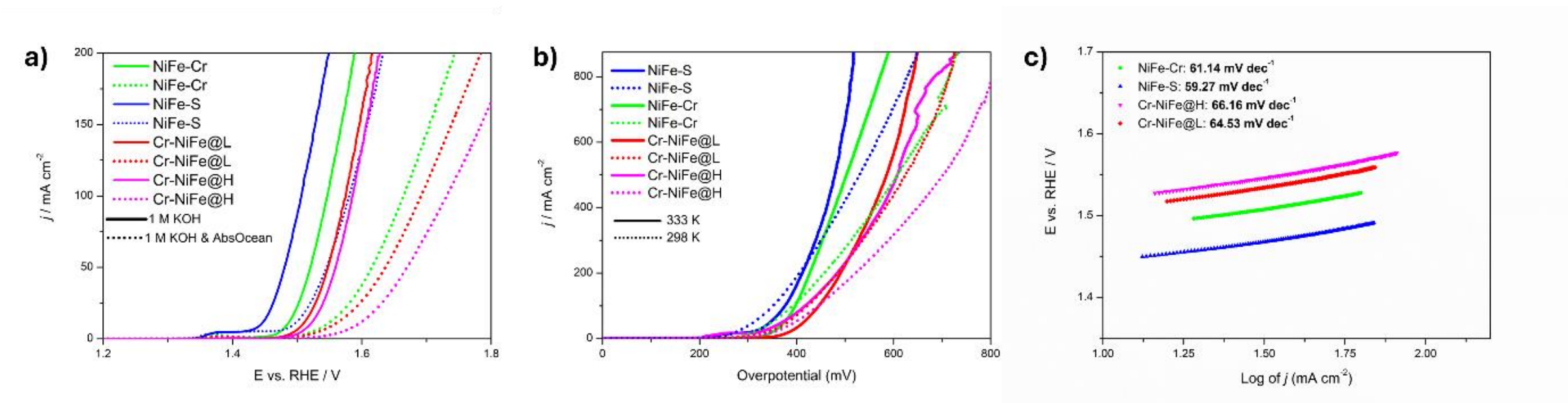


Figure 12 a) LSV curves of catalyst-coated mesh in 1 M KOH & 1 M KOH + AbsOcean at 298K at a scan rate of 1 $mVs^{-1}$ with an 80% iR compensation b) LSV curves of catalyst-coated foam in 1 M KOH & AbsOcean at 298K vs 333K at a scan rate of 1 $mVs^{-1}$ with an 80% iR compensation c) Tafel analysis of catalyst-coated mesh in 1 M KOH at 298K at a scan rate of 10 $mVs^{-1}$.

## References


1 J. Corbin, C. Lyu, D. Trudgeon, M. Jones, A. Loh, A. Graf, Z. Zhang, J. Cao, I. Nawrocka and X. Li, *RSC Sustainability*, DOI:10.1039/D5SU00650C.

2 F. Sun, J. Qin, Z. Wang, M. Yu, X. Wu, X. Sun and J. Qiu, *Nat. Commun.*, DOI:10.1038/s41467-021-24529-3.

3 C. Lyu, A. Loh, M. Jones, D. Trudgeon, J. Corbin, J. Cao, Z. Zhang, P. Connor and X. Li, *Chemistry – A European Journal*, DOI:10.1002/chem.202403030.

4 W. Cai, R. Chen, H. Yang, H. B. Tao, H.-Y. Wang, J. Gao, W. Liu, S. Liu, S.-F. Hung and B. Liu, *Nano Lett.*, 2020, **20**, 4278–4285.

5 S. Anantharaj and S. Noda, *Small*, DOI:10.1002/smll.201905779.

6 S. Akinkuade, J. Nel and W. Meyer, *Structural, optical and electrical characteristics of nickel oxide thin films synthesised through chemical processing method*, .

7 F. Davar, Z. Fereshteh and M. Salavati-Niasari, *J. Alloys Compd.*, 2009, **476**, 797–801.

8 Z. Wei, H. Qiao, H. Yang, C. Zhang and X. Yan, *J. Alloys Compd.*, 2009, **479**, 855–858.

9 J. Lee, Y. Choi, J. Kim, E. Park and K. Lee, *Materials Today Sustainability*, 2025, **31**, 101202.

10 H. An, W. Park, H. Shin and D. Y. Chung, *EcoMat*, DOI:10.1002/eom2.12486.

11 D. Y. Chung, P. P. Lopes, P. Farinazzo Bergamo Dias Martins, H. He, T. Kawaguchi, P. Zapol, H. You, D. Tripkovic, D. Strmcnik, Y. Zhu, S. Seifert, S. Lee, V. R. Stamenkovic and N. M. Markovic, *Nat. Energy*, 2020, **5**, 222–230.

12 I. S. Filimonenkov, S. Ya. Istomin, E. V. Antipov, G. A. Tsirlina and E. R. Savinova, *Electrochim. Acta*, 2018, **286**, 304–312.

13 C. Yang, M. Batuk, Q. Jacquet, G. Rousse, W. Yin, L. Zhang, J. Hadermann, A. M. Abakumov, G. Cibin, A. Chadwick, J.-M. Tarascon and A. Grimaud, *ACS Energy Lett.*, 2018, **3**, 2884–2890.

14 J. G. Vos and M. T. M. Koper, *Journal of Electroanalytical Chemistry*, 2019, **850**, 113363.

15 Y. Che, J. Shang, Y. Zhang, S. Bo, J. Zhang, X. Qin, X. Liu, H. Sun, W. Zhou, Y. Jiang, X. Chen, S. He, D. Ma, F. Pan and Q. Liu, *Adv. Funct. Mater.*, DOI:10.1002/adfm.202507544.

16 C. Hitz and A. Lasia, *Journal of Electroanalytical Chemistry*, 2002, **532**, 133–140.

17 N. K. Shrestha, S. A. Patil, J. Han, S. Cho, A. I. Inamdar, H. Kim and H. Im, *J. Mater. Chem. A Mater.*, 2022, **10**, 8989–9000.

18 X. An, Q. Hu, W. Zhu, L. Liu, Y. Zhang and J. Zhao, *Applied Physics A*, 2021, **127**, 865.

19 S. Jung, R. A. Senthil, A. Min, A. Kumar, C. J. Moon, G. H. Jeong, T. W. Kim and M. Y. Choi, *J. Mater. Chem. A Mater.*, 2024, **12**, 8694–8706.

20 F. Dionigi and P. Strasser, *Adv. Energy Mater.*, DOI:10.1002/aenm.201600621.

21 A. Seijas-Da Silva, A. Hartert, V. Oestreicher, J. Romero, C. Jaramillo-Hernández, L. J. J. Muris, G. Thorez, B. J. C. Vieira, G. Ducourthial, A. Fiocco, S. Legendre, C. Huck-Iriart, M. Mizrahi, D. López-Alcalá, A. T. S. Freiberg, K. J. J. Mayrhofer, J. C. Waerenborgh, J. J.

Baldoví, S. Cherevko, M. Varela, S. Thiele, V. Lloret and G. Abellán, *Nat. Commun.*, 2025, **16**, 6138.

22 S. Liu, H. Zhang, E. Hu, T. Zhu, C. Zhou, Y. Huang, M. Ling, X. Gao and Z. Lin, *J. Mater. Chem. A Mater.*, 2021, **9**, 23697–23702.

23 A. Zaffora, B. Megna, B. Seminara, F. Di Franco and M. Santamaria, *Nanomaterials*, 2024, **14**, 407.

24 Y. Xing, Y. Gao, D. Zhang, J. Huang, Q. Dong, Q. Tang, Y. Wang, Z. Ye and P. Liang, *J. Adv. Res.*, DOI:10.1016/j.jare.2025.11.026.

25 S. Liangliang, G. Qiqi, C. Zihao, L. Kangyi, J. Yifei, D. Xingbiao, F. Yuyang, S. Yunmei, L. Jie, L. Zezheng, X. Lirong and Y. Jipeng, *ChemCatChem*, DOI:10.1002/cctc.202501388.

26 C. Liu, Z. Teng, X. Liu, R. Zhang, J. Chi, J. Zhu, J. Qin, X. Liu, Z. Wu and L. Wang, *Chem. Sci.*, 2025, **16**, 16321–16330.

27 Z. Cai, J. Liang, Z. Li, T. Yan, C. Yang, S. Sun, M. Yue, X. Liu, T. Xie, Y. Wang, T. Li, Y. Luo, D. Zheng, Q. Liu, J. Zhao, X. Sun and B. Tang, *Nat. Commun.*, 2024, **15**, 6624.

**Decision on Nature Communications manuscript NCOMMS-25-97645 – Response to reviewer comments – round two**

**REVIEWER COMMENTS**

Reviewer #1 (Remarks to the Author):

The authors have addressed my concerns now, and this work can be published now.

We thank the reviewer for this comment, and we are thrilled that the manuscript meets your standards and has addressed your concerns. Thank you for your time.

Reviewer #2 (Remarks to the Author):

I have completed my review of this manuscript. Overall, I find it to be a well-written and scientifically sound piece of work. The research question is clear, the methodology is appropriate, the results are presented clearly, and the conclusions are supported by the data. The manuscript fits well within the scope of Nature Communications.
Therefore, I am pleased to recommend that this manuscript be accepted for publication in its current form.

We really thank the reviewer for these encouraging and kind words, and we are glad that the manuscript meets the reviewer's requirements.

Reviewer #3 (Remarks to the Author):

publish as is

We thank the reviewer for accepting the manuscript and are glad that it meets the requirements.
